# Photochemical Oxygen‐Atom‐Transfer Reactivity of Platinum(II) Peroxycarbonates

**DOI:** 10.1002/anie.5181266

**Published:** 2026-09-24

**Authors:** Meghan G. Sullivan, Jesse B. Gordon, Brandon M. Campbell, Shao‐Liang Zheng, Daniel G. Nocera

**Affiliations:** ^1^ Department of Chemistry and Chemical Biology Harvard University Cambridge Massachusetts USA; ^2^ Present address: Department of Chemistry Northwestern University Evanston Illinois USA

**Keywords:** oxygen atom transfer, peroxycarbonate, phosphine, photochemistry, Pt(II)

## Abstract

Peroxycarbonate (CO_4_^2–^) is implicated in various biological, catalytic, and energy storage processes – despite the capability of transition metals to guide the reactivity of this unstable intermediate toward productive ends, the coordination chemistry of metal–peroxycarbonate complexes, M(CO_4_), remains poorly understood. Herein, we describe the synthesis, isolation, and structural characterization of two platinum(II) peroxycarbonate complexes Pt(PPh_3_)_2_(CO_4_) (**1**) and Pt(dppe)(CO_4_) (**2**) (dppe = 1,2‐bis(diphenylphosphino)ethane). Whereas the complexes are thermally stable, they react by oxygen‐atom transfer (OAT) when irradiated with visible light. This photoreactivity was investigated using NMR and FT‐IR photolysis to identify structures of reaction intermediates and photoproducts, together with kinetic analyses, which establish unimolecular OAT from the CO_4_^2−^ ligand to the bound phosphine. In addition to providing rare examples of structurally characterized M(CO_4_) complexes, this work is the first demonstration of photochemical reactivity in a peroxycarbonate complex.

## Graphical abstract

The coordination chemistry of metal–peroxycarbonate complexes (M(CO_4_)) remains poorly understood, with no reported studies of the photochemical reactivity of M(CO_4_) complexes. The platinum(II) peroxycarbonate complexes Pt(PPh_3_)_2_(CO_4_) and Pt(dppe)(CO_4_) (dppe = 1,2‐bis(diphenylphosphino)ethane) have been structurally characterized and are shown to undergo photochemical, unimolecular OAT from the CO_4_^2−^ ligand to the bound phosphine. Kinetics studies establish a unimolecular OAT reaction.

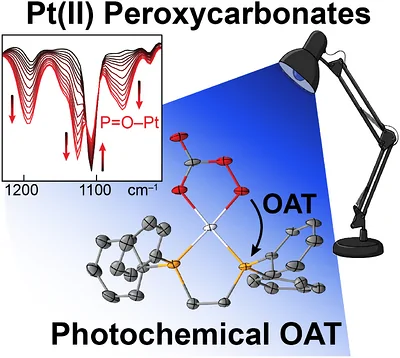

The reactivity of metal‐peroxides has long been appreciated in the context of biology, catalysis, and energy storage technologies [1, 2, 3, 4, 5]. The buildup and destruction of peroxide intermediates is influenced by CO_2_/HCO_3_^−^ speciation [6, 7, 8] in the function of metalloenzymes, such as Cu, Zn‐superoxide dismutase [9, 10], the oxidation of water to H_2_O_2_ [11, 12, 13], and the irreversibility of metal‐air batteries [14, 15]. The important intermediates for the reaction of CO_2_/HCO_3_^–^ with peroxide are peroxy(bi)carbonates, formed from the following reactions [16, 17].

(1) $$CO_{2}+H_{2}O_{2}\;⇌{\;\mathrm{CO}}_{4}^{2-}+2H^{+}$$

(2) $${\mathrm{HCO}}_{3}^{-}+H_{2}O_{2}\;⇌{\;\mathrm{HCO}}_{4}^{-}+H_{2}O$$

Though peroxy(bi)carbonate intermediates may influence the net reactivity of metal‐peroxides, the coordination chemistry of well‐defined metal–peroxycarbonate complexes (M(CO_4_)) remains largely unexamined. The first proposed M(CO_4_) complex was reported by Wilkinson as an impurity in the synthesis of Pt(PPh_3_)_2_(CO_3_) [18]. Avoiding reduction of this impurity led to the isolation of Pt(PPh_3_)_2_(CO_4_) [19]. Although not crystallographically characterized, the proposed peroxycarbonate was supported by a characteristic O─O stretch (778 cm^−1^). Since the first report of a crystallographically characterized M(CO_4_) complex [20, 21], a limited number of examples of well‐defined M(CO_4_) complexes have appeared [22, 23, 24, 25, 26, 27]. An even smaller subset of these complexes includes studies of reactivity, which ranges from oxygen‐atom transfer (OAT) [28, 29] to peroxycarbonate serving as a peroxide source [27]. There have been no studies describing photochemical reactivity of M(CO_4_) complexes.

Two methods to synthesize Pt(PPh_3_)_2_(CO_4_) (**1**) are shown in Scheme 1. Adapted from the original synthesis of **1** using the Pt(0) complex [18], bubbling O_2_ and CO_2_ through a solution of Pt(PPh_3_)_4_ yields a mixture of **1** and Pt(PPh_3_)_2_(CO_3_), the latter of which can be completely converted to **1** via treatment with H_2_O_2._ Alternatively, salt metathesis from the Pt(II) complex, Pt(PPh_3_)_2_Cl_2_, with Ag_2_CO_3_ yields Pt(PPh_3_)_2_(CO_3_), which has been structurally characterized (Figure S1, Table S1), and subsequent treatment with excess H_2_O_2_ then furnishes **1**. **1** is stable under ambient conditions for at least three months in the solid state and is thermally stable in acetonitrile (CH_3_CN) solution at 50 °C for up to 8 h monitored by ^31^P{^1^H} NMR spectroscopy (Figure S6). Light yellow crystals of **1** were isolated from a dichloromethane (CH_2_Cl_2_)/diethyl ether (Et_2_O) solution. The single‐crystal x‐ray structure reveals the peroxycarbonate ligand to be disordered (Figure S2, Table S2). Figure 1 shows the crystal structure of **1** with the two‐part disorder omitted for clarity. Whereas the O─Pt─O bite angle of Pt(PPh_3_)_2_(CO_3_) is 64.6(1)°, this bite angle expands to 79.9(2)° in **1**. The DFT geometry‐optimized structure of **1** (Table S6) reproduces the O─Pt─O bite angle (∠_calcd_(O─Pt─O)  = 80.7°) as well as the O─O bond distance (d_exp_(O─O)  = 1.48(1) Å, d_calcd_(O─O)  = 1.49  Å). The solid‐state FT‐IR spectrum of **1** exhibits a diagnostic signature for the ν_sym_(O─O) at 779 cm^−1^, together with ν_sym_(C═O) and ν_asym_(C═O) at 1681 and 1249 cm^−1^, respectively, consistent with comparison to ^13^C labeled spectra and DFT calculated frequencies (Figure S7).

**SCHEME 1:**
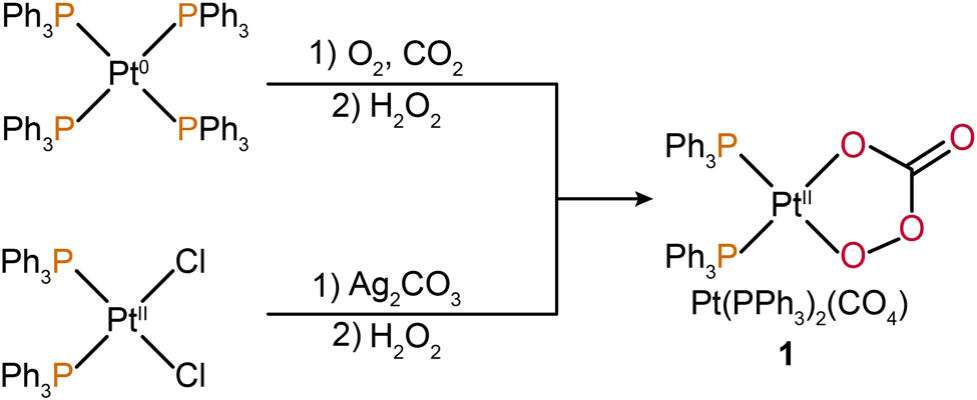
Synthetic routes to generate **1** from Pt^0^ or Pt^II^ precursors.

**FIGURE 1:**
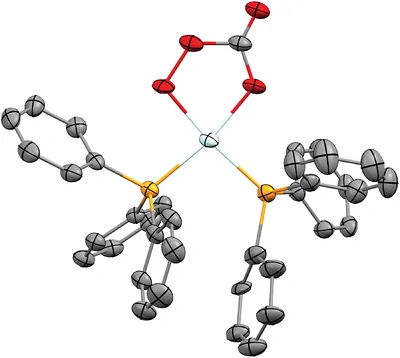
Crystal structure of **1**. Atomic displacement parameters set at 50% probability level. Hydrogen atoms, solvent molecules (CH_2_Cl_2_), and disorder are omitted for clarity. Color scheme: C, gray; O, red; P, orange; Pt, light blue.

Compared to the ^1^H, ^13^C, and ^31^P NMR spectra of the C_2_‐symmetric Pt(PPh_3_)_2_(CO_3_) complex, the NMR spectra of **1** reflect a loss in symmetry (Figure S8). The aromatic region of ^13^C{^1^H} NMR spectrum of Pt(PPh_3_)_2_(CO_3_) breaks into a series of doublets for the two inequivalent sets of phenyl‐groups on each PPh_3_ of **1**. Additionally, a characteristic doublet‐of‐doublets at 168.6 ppm (*J*_C–P_ = 5.8 Hz; 3.3 Hz), attributed to the carbon of the peroxycarbonate (CO_4_) of **1**, is distinct from the observed triplet at 167.6 ppm (*J*_C–P_ = 3.9 Hz) for the carbon (CO_3_) of Pt(PPh_3_)_2_(CO_3_). The ^31^P{^1^H} NMR spectrum of **1** shows two inequivalent ^31^P nuclei, with coupling to each other (^2^ *J*_P–P_ = 25 Hz) and to ^195^Pt (^1^ *J*_P–Pt_ = 3850 and 3287 Hz). The ^195^Pt NMR spectrum of **1** (Figure S9) shows one resonance centered at −3949 ppm, split into a doublet‐of‐doublets by two unique ^31^P nuclei, consistent with the coupling constants observed in the ^31^P NMR spectrum.

Blue LED excitation (λ_exc_ = 440 nm) of **1** in CH_3_CN (Figure S10) leads to generation of 1 equiv of OPPh_3_ and Pt(PPh_3_)(CH_3_CN)(CO_3_) as monitored by ^31^P NMR spectroscopy (Figure S11). This photoconversion is supported by the appearance of free OPPh_3_ at 26 ppm and the growth of a single resonance at 2.50 ppm with coupling to ^195^Pt (^1^ *J*_P–Pt_ = 3898 Hz), consistent with a single Pt‐bound PPh_3_ for Pt(PPh_3_)(CH_3_CN)(CO_3_). Additionally, the solution‐state IR spectrum for the photolytic conversion of **1** to Pt(PPh_3_)(CH_3_CN)(CO_3_) shows ν_sym_(C═O) increase in energy by 17 cm^−1^, and ν_asym_(C═O) decrease in energy by 62 cm^−1^ (Figure S12A); these results are in good agreement with the expected IR shifts (Δν_sym_(C═O)_calcd_ = 18 cm^−1^, Δν_asym_(C═O)_calcd_ = 66 cm^−1^) as determined by DFT geometry optimization and frequency calculations for both **1** and Pt(PPh_3_)(CH_3_CN)(CO_3_) (Figure S12B). No resonances are observed for Pt‐coordinated OPPh_3_ in the expected region of >50 ppm in the ^31^P{^1^H} NMR [30] spectrum (Figure S11), suggesting that the OPPh_3_ formed from OAT to PPh_3_ is rapidly exchanged by CH_3_CN to yield Pt(PPh_3_)(CH_3_CN)(CO_3_). This OAT photoproduct is in line with ^18^O labeling studies of Rh(CO_4_) complexes that undergo thermal OAT to phosphines, where the metal‐bound peroxo‐oxygen is susceptible to transfer [29]. While **1** showed excellent thermal stability (Figure S6), irradiation of **1** in the presence of excess PPh_3_ (3 equiv) leads to the quantitative generation of OPPh_3_ and Pt(PPh_3_)_2_(CO_3_) (Figure S13), which results from the displacement of CH_3_CN by PPh_3_ (Scheme 2). Complete photochemical conversion of **1** occurs within 15 min as monitored by ^31^P NMR spectroscopy. Conversely, the reaction of **1** with PPh_3_ in the dark, under the same conditions used in photolysis experiments, shows sluggish reactivity, with the OAT reaction occurring over 9 h (Figure S14).

**SCHEME 2:**
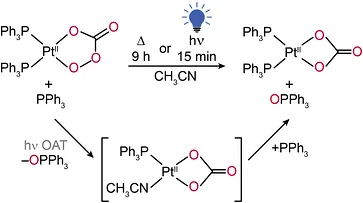
Thermal and photochemical (*λ*_exc_ = 440 nm LED) OAT reactivity of **1** in CH_3_CN with PPh_3_ (3 equiv). Photolysis leads to a 36x rate enhancement.

The kinetics for the photolysis of **1** was monitored by ^31^P{^1^H} NMR spectroscopy (Figure S15A). The initial rate for the disappearance of **1** is equivalent to the rate of formation of OPPh_3_ (Figure S15B) with a first‐order dependence on the concentration of **1** (Figure S15C). The kinetics scale linearly with light intensity, indicating that the photoreaction is a one‐photon process. These results suggest that the OAT from CO_4_^2−^ to PPh_3_ is unimolecular, implying a OPPh_3_‐coordinated intermediate, Pt(PPh_3_)(OPPh_3_)(CO_3_), precedes the appearance of Pt(PPh_3_)(CH_3_CN)(CO_3_).

To observe if Pt(PPh_3_)(OPPh_3_)(CO_3_) was the initial photoproduct, the photochemistry of **1** was re‐examined in non‐coordinating solvents such as CH_2_Cl_2_. Irradiation (*λ*_exc_ = 440 nm) of **1** in CH_2_Cl_2_ results in the appearance of three new peaks in the ^31^P{^1^H} NMR spectrum with concomitant decay of signals belonging to complex **1** (Figure S16). A singlet at 27 ppm is assigned to free OPPh_3_, and a doublet at 53 ppm (*J*_P–P_ = 3.8 Hz) with coupling to another doublet at 3.6 ppm (*J*_P–P_ = 3.8 Hz) is consistent with a coordinated phosphine‐oxide in Pt(PPh_3_)(OPPh_3_)(CO_3_). FT‐IR monitored photolysis in CH_2_Cl_2_ shows a new band at 1064 cm^−1^ (Figure S17) that is consistent with Pt(PPh_3_)(OPPh_3_)(CO_3_), containing a P═O band shifted to lower frequency upon coordination of the oxygen to a metal center [30]. As in CH_3_CN, photolysis of **1** in CH_2_Cl_2_ obeys first‐order kinetics, with unimolecular reaction rates comparable to the reaction in CH_3_CN (Figure S18).

Although evidence of a Pt‐bound phosphine‐oxide was observed upon photolysis of **1** in CH_2_Cl_2_, free OPPh_3_ remained the major photoproduct. Therefore, the complementary bidentate phosphine complex, Pt(dppe)(CO_4_) (dppe = 1,2‐bis(diphenylphosphino)ethane) (**2**) was prepared by treating Pt(dppe)(CO_3_) (x‐ray structure, Figure S3, Table S3; NMR, Figure S19) with H_2_O_2_ to generate **2**, which was crystallographically (Figure S4, Table S4) and spectroscopically (Figures S19, S20) characterized. **2** exhibits a similar O─Pt─O bite angle (79.9(6)°) and O─O bond length (d_exp_(O─O)  = 1.46(2)  Å) to that of **1**, and the crystal metrics agree well with the DFT geometry‐optimized structure (∠_calcd_(O─Pt─O)  = 80.7°, d_calcd_(O─O) = 1.49 Å) (Table S9). The solid‐state FT‐IR spectrum of **2** (Figure S20) exhibits the same diagnostic ν_sym_(O─O) and ν_asym_(C═O) as **1** at 779 and 1249 cm^−1^, respectively, along with a ν_sym_(C═O) at 1668 cm^−1^ shifted to slightly lower energy than observed for **1**. We hypothesized that the photochemical OAT reactivity may still be present for **2**, but that the bidentate phosphine ligand could slow dissociation of the phosphine oxide and thus allow the initial photoproduct to be generated quantitatively.

Blue LED excitation of **2** in CH_2_Cl_2_ (Figure S21) results in clean photoreactivity. ^31^P NMR spectroscopy shows conversion of **2** to a single species upon irradiation (Figure 2), with the complete time course of the reaction shown in Figure S22. The peak observed at 54.3 ppm (^3^ *J*_P–P_ = 7.3 Hz) is assigned to a phosphine oxide coordinated to Pt, as evidenced by the significant downfield shift relative to free dppeO (+22.5 ppm) (dppeO = 1,2‐bis(diphenylphosphino) ethane monoxide). The coupled signal at −4.0 ppm (^3^ *J*_P–P_  = 7.3 Hz; ^1^ *J*_P–Pt_ = 4332 Hz) is assigned to a Pt‐bound phosphine, consistent with the quantitative conversion of **2** to a Pt(dppeO)(CO_3_) photoproduct. FT‐IR monitored photolysis in CH_2_Cl_2_ (Figure 3) corroborates the ^31^P NMR results; the FT‐IR features an isosbestic point for the decay of **2** (at 1674, 1257 cm^−1^) and growth of Pt(dppeO)(CO_3_) (at 1689, 1196, 1126, 1064 cm^−1^) with no features of unbound dppeO present. During photolysis, **2** is consumed at the same rate as Pt(dppeO)(CO_3_) is generated (Figure 4). First‐order kinetics with respect to Pt are observed, suggesting a photochemical unimolecular OAT reaction forming Pt(dppeO)(CO_3_) from **2**, similar to that seen for **1**. TD‐DFT calculations of the S_n_ excited state manifold (Table S13) show the lowest energy excited state to exhibit both O─O antibonding (h^+^) character within the peroxycarbonate ligand and d_x2–y2_ Pt─O antibonding (e^−^) character (Figure S27), suggesting the photochemistry to be derived from ligand(peroxy)‐to‐metal charge transfer. A quantum yield measurement for the conversion of **2** to Pt(dppeO)(CO_3_) was carried out with 440 nm laser excitation. Even at low conversion yields (4%), there is an inner‐filter effect due to photoproduct absorption, and thus we are only able to place a lower bound on the quantum yield. With this caveat, we measure a quantum yield of at least 94% ± 4% for the transformation of **2** into Pt(dppeO)(CO_3_), establishing a high efficiency for the OAT photochemistry of peroxycarbonate.

**FIGURE 2:**
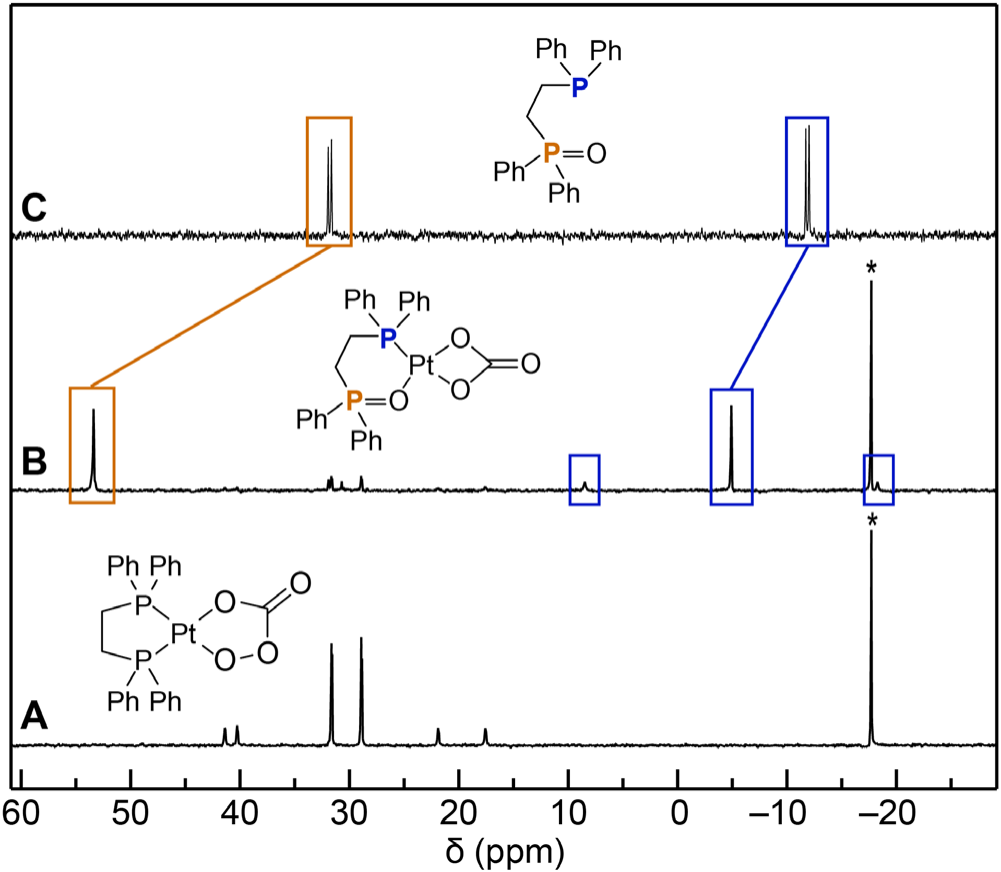
^31^P{^1^H} NMR spectra of CH_2_Cl_2_ solutions of **2** (20 mM) (A) before and (B) after 720 s of photolysis (*λ*_exc_ = 440 nm LED). Peaks of the photolysis product, Pt(dppeO)(CO_3_), are assigned (orange and blue boxes) and compared with (C) free dppeO. Asterisks (*) correspond to an OP(OPh)_3_ internal standard.

**FIGURE 3:**
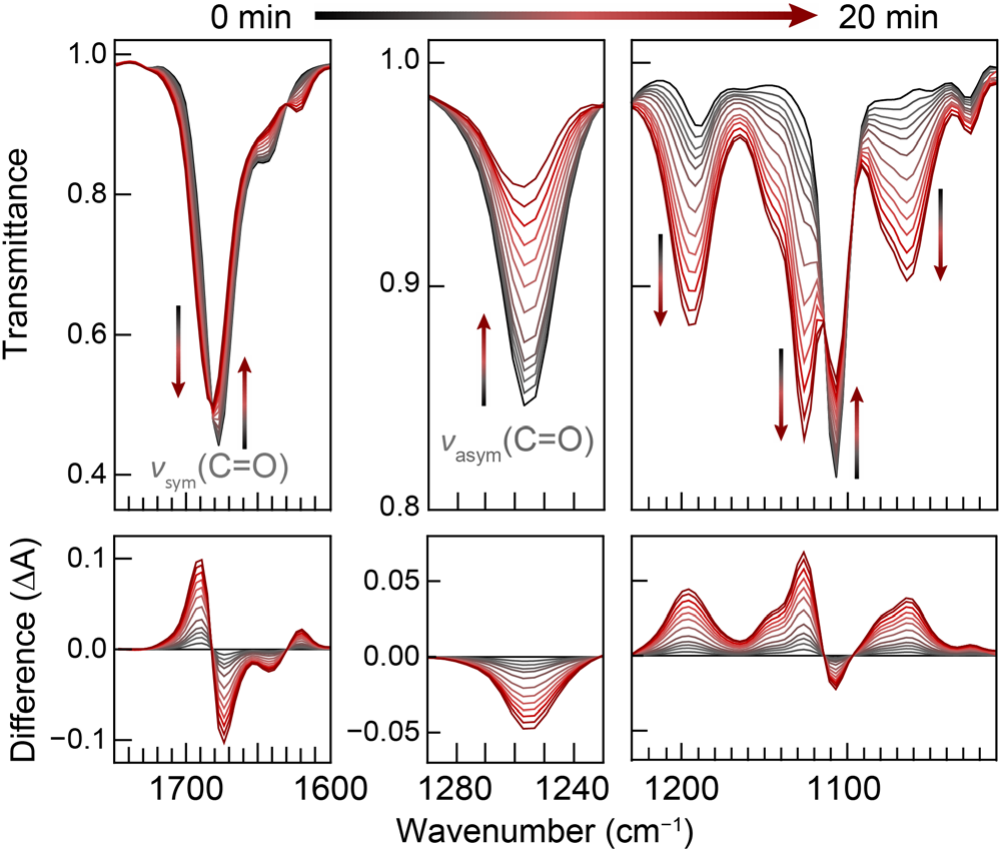
Solution‐state FT‐IR and difference spectra of **2** (5 mM) in CH_2_Cl_2_ over the course of a 20 min photolysis (*λ*_exc_ = 440 nm LED).

**FIGURE 4:**
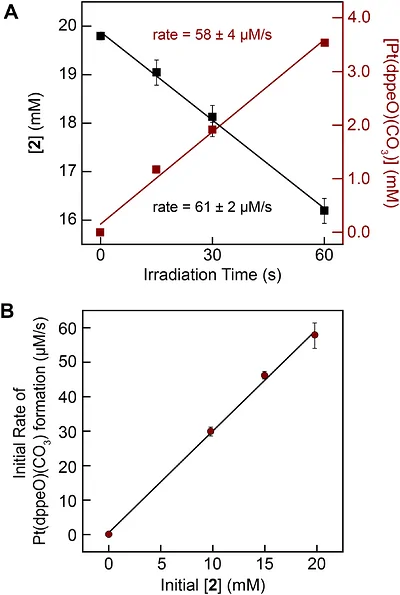
(A) Initial rates of **2** consumption (black) and Pt(dppeO)(CO_3_) formation (red). (B) Initial rate analysis showing a first‐order relationship between rate and initial [**2**]. Each data point represents the average of three measurements on three different samples. Where error limits are not indicated, they fall within the symbol.

The unimolecular OAT photoreaction is distinguished from the thermal reactivity of Pd(CO_4_) peroxycarbonate complexes, which undergo dissociation to generate a CO_4_^2−^ salt [28]. In this case, the addition of halide promotes the liberation of CO_4_^2−^, thus enhancing the reaction rate. For a unimolecular OAT, such a Cl^−^ dependence should not be observed. To explore whether Cl^−^ perturbs the observed OAT photochemistry, **2** was mixed with an excess of PPN[Cl] (6 equiv) in CH_2_Cl_2_. No ground‐state reaction was observed between **2** and PPN[Cl] over the course of two days at room temperature, as monitored by ^31^P{^1^H} NMR spectroscopy. Upon photolysis (*λ*_exc_ = 440 nm), the initial rate of **2** (21 mM) consumption was 66 ± 2 µM/s (Figure S23A), which is in line with the initial rates measured for **2** in the absence of halide (Figure S23B). As the presence of excess Cl^−^ does not affect the reaction kinetics, a dissociative mechanism where free CO_4_^2−^/HCO_4_^−^ is the reactive species is unlikely to contribute to the photochemical OAT reaction.

The P═O of the dppeO ligand on the initial OAT photoproduct, Pt(dppeO)(CO_3_), is cleanly substituted by CH_3_CN to furnish Pt(dppeO)(CH_3_CN)(CO_3_) as observed by ^31^P NMR (Figure S24) and FT‐IR (Figure S25) spectroscopies. The ^31^P{^1^H} shift of the P═O on Pt(dppeO)(CH_3_CN)(CO_3_) is within 1 ppm of the P═O of free dppeO, suggesting the P═O is solvent‐exposed, as expected. To obtain a crystalline OAT photoproduct for additional structural validation, **2** was photolyzed in CH_2_Cl_2_ to furnish Pt(dppeO)(CO_3_) followed by the addition of five equiv of thioanisole (PhSMe), which again cleanly displaces the P═O of the dppeO ligand, in this case to furnish Pt(dppeO)(PhSMe)(CO_3_) (Figure S26). The crystal structure of Pt(dppeO)(PhSMe)(CO_3_), shown in Figure 5, establishes unimolecular OAT resulting in the phosphine oxide, with the dppeO ligand remaining anchored to Pt via the unoxidized phosphine. These products provide conclusive evidence for the reaction sequence comprising a photochemical OAT about a single Pt center, followed by P═O photoproduct dissociation (Figure 5).

**FIGURE 5:**
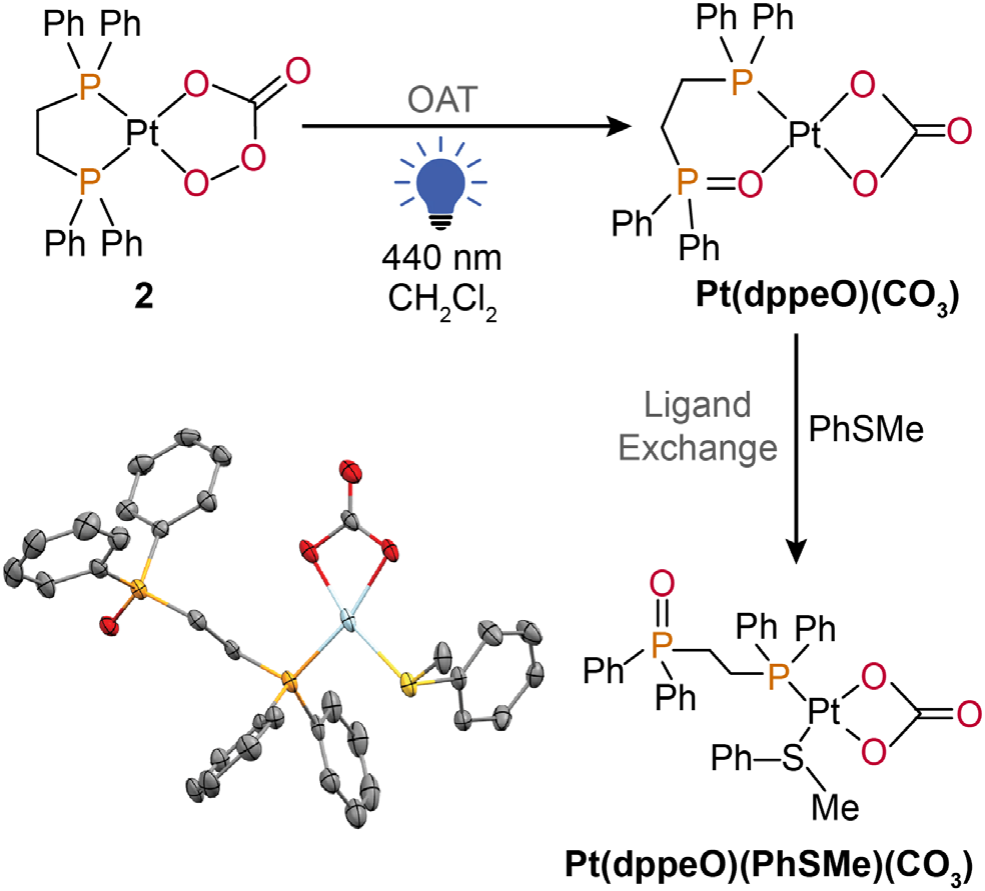
Unimolecular OAT photolysis scheme from **2** to Pt(dppeO)(CO_3_), followed by ligand exchange to yield Pt(dppeO)(PhSMe)(CO_3_). Crystal structure of Pt(dppeO)(PhSMe)(CO_3_) shown to the left. Atomic displacement parameters set at 50% probability level. Hydrogen atoms are omitted for clarity. Color scheme: C, gray; O, red; P, orange; S, yellow; Pt, light blue.

Investigations of peroxycarbonate reactivity remain underexplored, but provide avenues to understand complex systems comprising carbonates, oxygen, and peroxide. In this work, the small library of crystallographically characterized M(CO_4_) complexes has been expanded to include **1** and **2**, and we show unique unimolecular OAT reactivity for peroxycarbonate that is induced by visible irradiation. The two facile synthetic routes to M(CO_4_) complexes demonstrated herein set a precedent for the synthesis of more diverse libraries of M(CO_4_) complexes and introduce a potentially vibrant photochemistry of peroxycarbonate.

## Author Contributions

**Meghan G. Sullivan**: conceptualization, investigation, methodology, validation, formal analysis, data curation, visualization, writing – original draft, writing – review and editing. **Jesse B. Gordon**: conceptualization, writing – review and editing, formal analysis, validation. **Brandon M. Campbell**: conceptualization, validation, formal analysis, and writing – review and editing. **Shao‐Liang Zheng**: formal analysis and visualization. **Daniel G. Nocera**: writing – review and editing, writing – original draft, conceptualization, funding acquisition, validation, formal analysis, project administration, supervision, resources.

## Funding

This work was supported by the National Science Foundation CHE‐2243724. We acknowledge support of the X‐ray core facility in the Department of Chemistry and Chemical Biology at Harvard University from the Major Research Instrumentation (MRI) Program of the National Science Foundation (NSF) under Award Number CHE‐2216066. MGS and BMC acknowledge support from the National Science Foundation Graduate Research Fellowship Program under Grant DGE‐2140743 and from the Herchel Smith Fellowship at Harvard University. JBG acknowledges support from an NIH Postdoctoral Fellowship under Grant Number F32GM147975.

## Conflicts of Interest

The authors declare no conflicts of interest.

## Supporting information



Materials and methods, synthetic procedures, crystallographic data, DFT calculations, UV–vis, FT‐IR, and NMR spectra. The authors have cited additional references within the Supporting Information [31–46]. **Supporting File**: anie74574‐sup‐0001‐SuppMat.pdf.

## Data Availability

Metrical data and supplementary crystallographic data are available from the Cambridge Crystallographic Data Centre under reference numbers CCDC  2556243, 2556244, 2556245, 2556246, and 2556247. These data can be obtained free of charge via www.ccdc.cam.ac.uk/data_request/cif or by emailing data_request@ccdc.cam.ac.uk or by contacting the Cambridge Crystallographic Data Centre, 12 Union Road, Cambridge CB2 1EZ, UK; fax: +44 1223 336033.
