# Supplementary material for: Photochemical Oxygen‐Atom‐Transfer Reactivity of Platinum(II) Peroxycarbonates
Source: Angew Chem Int Ed Engl. 2026 Sep 24;65(45):e5181266. doi: 10.1002/anie.5181266 (PMC13725566; doi:10.1002/anie.5181266)
Supplement: Supplementary file 1 [file ANIE-65-e5181266-s001.pdf]

Supporting Information

# Photochemical Oxygen-Atom-Transfer Reactivity of Platinum(II) Peroxycarbonates

Meghan G. Sullivan, Jesse B. Gordon, Brandon M. Campbell, Shao-Liang Zheng, and Daniel G. Nocera*

*Department of Chemistry and Chemical Biology, Harvard University, 12 Oxford Street, Cambridge, MA 02138, United States*

*Corresponding author email: dnocera@fas.harvard.edu

# Table of Contents


A. Materials and Methods S4

A.1. General Considerations S4

A.2. Synthesis of Compounds S4

A.3. Instrumentation S8

A.4. Crystallography S8

A.5. Computational Methods S9

A.6. Photochemical Methods S9

B. X-Ray Crystallography S11

B.1. $Pt(PPh_3)_2(CO_3)$ and **1** S11

**Figure S1**. Crystal structure of $Pt(PPh_3)_2(CO_3)$ S11

**Table S1**. Crystallographic data for $Pt(PPh_3)_2(CO_3)$ S12

**Figure S2**. Crystal structure of **1** S13

**Table S2**. Crystallographic data for **1** S14

B.2. $Pt(dppe)(CO_3)$ and **2** S15

**Figure S3**. Crystal structure of $Pt(dppe)(CO_3)$ S15

**Table S3**. Crystallographic data for $Pt(dppe)(CO_3)$ S16

**Figure S4**. Crystal structure of **2** S17

**Table S4**. Crystallographic data for **2** S18

B.3. $Pt(dppeO)(PhSMe)(CO_3)$ Photoproduct S19

**Figure S5**. Crystal structure of $Pt(dppeO)(PhSMe)(CO_3)$ S19

**Table S5**. Crystallographic data for $Pt(dppeO)(PhSMe)(CO_3)$ S20

C. Compound 1 S21

C.1. Spectroscopic Characterization S21

**Figure S6.** $^{31}P\{^1H\}$ NMR spectra of **1** at 50 °C S21

**Figure S7.** FT-IR spectra of **1** S21

**Figure S8.** $^1H$, $^{13}C\{^1H\}$ and $^{31}P\{^1H\}$ NMR spectra of $Pt(PPh_3)_2(CO_3)$ and **1** S22

**Figure S9.** $^{195}Pt$ NMR spectrum of **1** S23

**Figure S10.** UV-vis spectrum of **1** in $CH_3CN$ S23

C.2. Reactivity S24

**Figure S11.** $^{31}P\{^1H\}$ NMR monitored photolysis of **1** in $CH_3CN$ S24

**Figure S12.** FT-IT monitored photolysis of **1** in $CD_3CN$ S25

**Figure S13.** $^{31}P\{^1H\}$ NMR of **1** + $PPh_3$ + hν in $CH_3CN$ S26

**Figure S14.** $^{31}P\{^1H\}$ NMR monitored reaction of **1** + $PPh_3$ in $CH_3CN$ S26

**Figure S15.** Kinetics of **1** photolysis in $CH_3CN$ S27

**Figure S16.** $^{31}P\{^1H\}$ NMR monitored photolysis of **1** in $CH_2Cl_2$ S28

**Figure S17.** FT-IT monitored photolysis of **1** in $CH_2Cl_2/CDCl_3$ S28

**Figure S18.** Kinetics of **1** photolysis in $CH_2Cl_2$ S29

D. Compound 2 S30

D.1. Spectroscopic Characterization S30

**Figure S19.** $^1H$, $^{13}C\{^1H\}$, and $^{31}P\{^1H\}$ NMR spectra of Pt(dppe)($CO_3$) and **2** S30

**Figure S20.** FT-IR spectra of **2** S31

**Figure S21.** UV-vis spectrum of **2** in $CH_2Cl_2$ S31

D.2. Reactivity S32

**Figure S22.** $^{31}P\{^1H\}$ NMR monitored photolysis of **2** in $CH_2Cl_2$ S32

**Figure S23.** Kinetics of **2** photolysis in $CH_2Cl_2$ in the presence of PPN[Cl] S32

**Figure S24.** $^{31}P\{^1H\}$ NMR showing Pt(dppeO)($CH_3CN$)($CO_3$) formation S33

**Figure S25.** FT-IR photolysis showing Pt(dppeO)($CH_3CN$)($CO_3$) formation S34

**Figure S26.** $^{31}P\{^1H\}$ NMR showing Pt(dppeO)($Me_2SO$)($CO_3$) formation S35

E. Density Functional Theory Calculations S36

E.1. Compound **1** and Related Calculations S36

**Table S6.** Optimized coordinates of **1** with $CH_2Cl_2$ implicit solvation S36

**Table S7.** Optimized coordinates of **1** with $CH_3CN$ implicit solvation S37

**Table S8.** Optimized coordinates of Pt($PPh_3$)($CH_3CN$)($CO_3$) with $CH_3CN$ implicit solvation S38

E.2. Compound **2** and Related Calculations S39

**Table S9.** Optimized coordinates of **2** with $CH_2Cl_2$ implicit solvation S39

**Table S10.** Optimized coordinates of **2** with $CH_3CN$ implicit solvation S40

**Table S11.** Optimized coordinates of Pt(dppeO)($CH_3CN$)($CO_3$) with $CH_3CN$ implicit solvation S41

**Table S12.** Optimized coordinates of Pt(dmpe)($CO_4$) with $CH_3CN$ implicit solvation S42

**Table S13.** $S_n$ from TD-DFT of Pt(dmpe)($CO_4$) S42

**Figure S27.** Visualization of TD-DFT for $S_1$ state of Pt(dmpe)($CO_4$) S43

F. References S44

## A. Materials and Methods

### A.1. General Considerations

Reactions were carried out in an $N_2$-filled glovebox unless otherwise noted. Benzene, pentane, and acetonitrile ($CH_3CN$) were purchased from Sigma Aldrich (HPLC grade). Dichloromethane ($CH_2Cl_2$) was purchased from Fisher Scientific (not stabilized/HPLC). Benzene, pentane, $CH_3CN$, and $CH_2Cl_2$ were purified using an argon-purged Pure Process Technologies solvent purification system, then dried over activated 3Å molecular sieves in an $N_2$-filled glovebox for at least 24 h prior to use. Deuterated solvents were degassed by a minimum of three freeze-pump-thaw cycles, then dried over activated 3Å molecular sieves in an $N_2$-filled glovebox for at least 24 h prior to use. Vials, pipettes, and other glassware were oven-dried at 140 °C overnight before being brought into the $N_2$-filled glovebox. $^{13}C$ labeled sodium carbonate and deuterated solvents were purchased from Cambridge Isotope Laboratories. Silver carbonate, urea hydrogen peroxide, and triphenyl phosphate were purchased from Sigma Aldrich. Tetrakis(triphenylphosphine)platinum(0), cis-bis(triphenylphosphine)platinum(II) chloride, dichloro(1,5-cyclooctadiene)platinum(II), and 1,2-bis(diphenylphosphino)ethane were purchased from AmBeed. Silver nitrate was purchased from Oakwood Chemical. Bis(triphenylphosphine)iminium chloride (PPN[Cl]) was purchased from Strem and recrystallized from $CH_2Cl_2$ before use. Ultra-high purity oxygen and industrial grade carbon dioxide gases were purchased from AirGas.

**CAUTION:** Although we encountered no safety incidents or violent reactivity with peroxide-containing compounds, caution should be exercised when working with peroxides. Urea hydrogen peroxide ($H_2O_2$-urea) is a strong oxidizer that can spontaneously combust when heated or in contact with organic/combustible materials. Reactions with $H_2O_2$-urea should be conducted with adequate venting and away from ignition sources. Additionally, the isolated products (**1** and **2**) are inorganic peroxides and should be handled with caution. As with other inorganic peroxo-species, exposure to heat, light, friction, mechanical shock, or contact with combustible, organic, or reducing materials should be avoided and manipulations should be carried out with caution, appropriate PPE, and engineering controls.

### A.2. Synthesis of Compounds

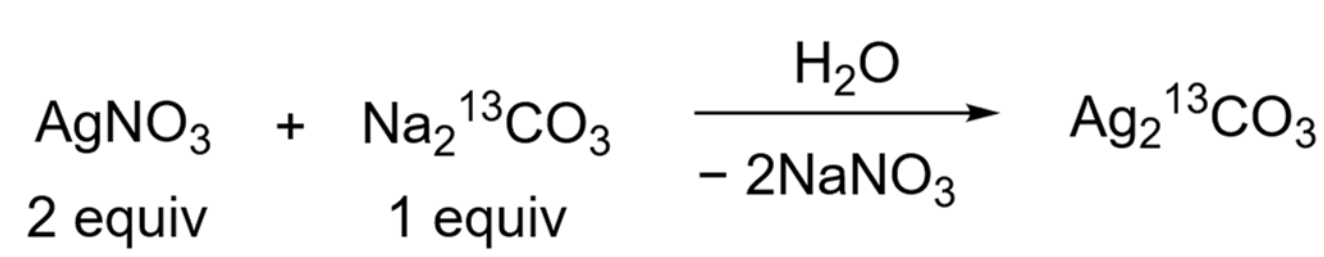


**$Ag_2{}^{13}CO_3$.** Reaction was carried out in ambient conditions and protected from light. $AgNO_3$ (500 mg, 2.94 mmol, 2.00 equiv) was dissolved in distilled water (5 mL) in a 20 mL scintillation vial. $Na_2{}^{13}CO_3$ (157 mg, 1.47 mmol, 1.00 equiv) was dissolved in distilled water (2 mL) in a separate 1-dram vial. The $Na_2{}^{13}CO_3$ solution was added to the $AgNO_3$ solution with stirring. The suspension was stirred for 5 min, after which it was filtered, and the solid washed with distilled water (1×10 mL) and diethyl ether (4×10 mL) before drying under vacuum. The final yellow solid (327 mg, 80%) was stored in darkness until use.

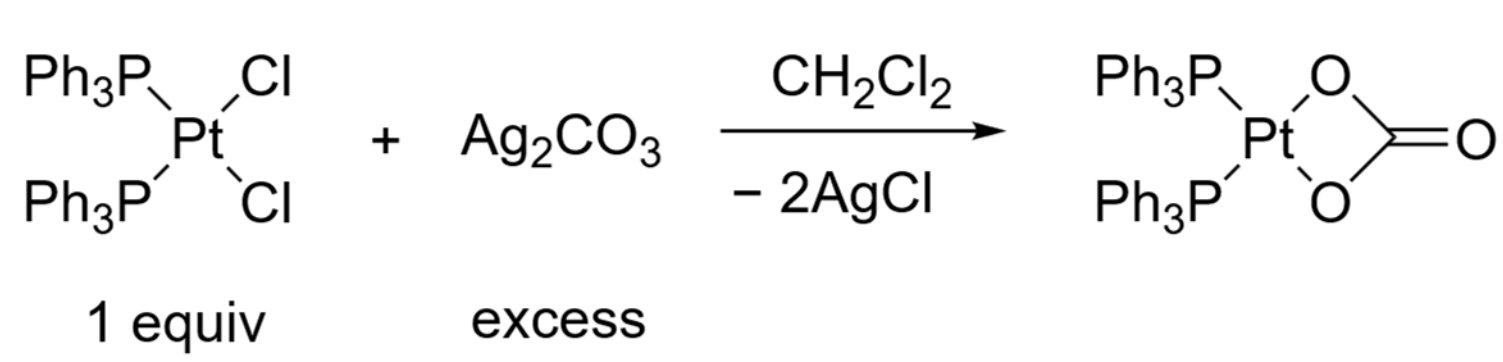

**$Pt(PPh_3)_2(CO_3)$ and $Pt(PPh_3)_2(^{13}CO_3)$**. Reaction was carried out in ambient conditions and protected from light. $Pt(PPh_3)_2Cl_2$ (300 mg, 0.38 mmol, 1 equiv) was dissolved in $CH_2Cl_2$ (10 mL) in a 20 mL scintillation vial. $Ag_2CO_3$ or $Ag_2{}^{13}CO_3$ (520 mg, 1.90 mmol, 5 equiv) was added. The vial was wrapped with aluminum foil, and the suspension was stirred for 48 h. After 48 h, an aliquot was removed for $^{31}P$ NMR analysis to confirm full conversion. The suspension was filtered through celite to remove AgCl and unreacted $Ag_2CO_3$. Solvent was removed from the filtrate to yield a white solid. The solid was washed with benzene (1×10 mL) and diethyl ether (2×10 mL) before drying under vacuum (162 mg, 58%). Colorless cubic crystals for X-ray diffraction were grown by vapor diffusion of diethyl ether into a $CH_2Cl_2$ solution of $(PPh_3)_2Pt(CO_3)$ at 4 °C.

$^1H$ NMR (400 MHz, $CDCl_3$) δ (ppm): 7.38 (m, 3H), 7.21 (m, 2H).

$^{13}C\{^1H\}$ NMR (151 MHz, $CDCl_3$) δ (ppm): 167.6 (t, $J_{C-P}$ = 3.9 Hz), 134.3 (t, $J_{C-P}$ = 5.5 Hz), 131.3 (s), 128.6 (t, $J_{C-P}$ = 5.5 Hz).

$^{31}P\{^1H\}$ NMR (162 MHz, $CDCl_3$) δ (ppm): 6.79 (s w/ $^{195}Pt$ satellites, $J_{P-Pt}$ = 3707 Hz).

FT-IR (solid, $cm^{-1}$): 1673, 1629, 1480, 1435, 1200, 1093, 986, 817, 754, 744, 727, 687, 557, 527.

FT-IR ($^{13}C$ labeled) (solid, $cm^{-1}$): 1636, 1479, 1435, 1142, 1096, 979, 790, 755, 744, 683, 555, 524.

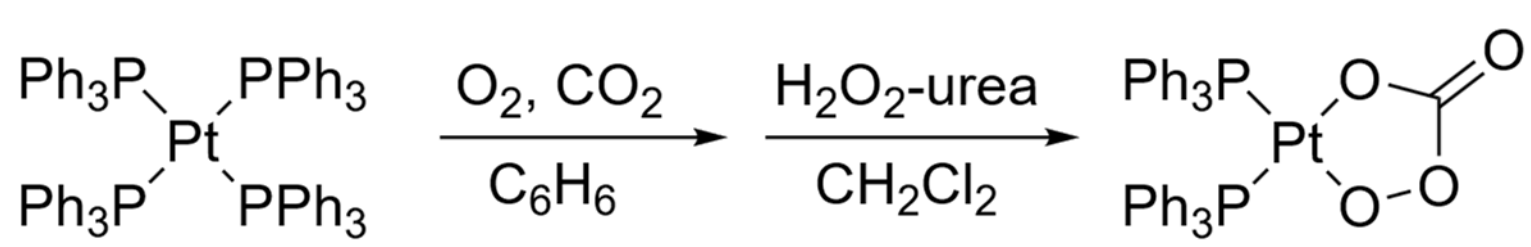


**$Pt(PPh_3)_2(CO_4)$ (1) from Pt(0)**. In a $N_2$-filled glovebox, $Pt(PPh_3)_4$ (2.50 g, 2.00 mmol) was dissolved in benzene (100 mL) within a 250 mL round-bottom-flask. The flask was sealed with a rubber septum and removed from the glovebox. The yellow solution was stirred while steady streams of $O_2$ and $CO_2$ were bubbled through, with a vent needle to keep the flask at 1 atm. After 8 h, the white precipitate was collected by filtration and dried under vacuum. This material, (1.36 g, 1.00 equiv), which is a mixture of 1 and $Pt(PPh_3)_2(CO_3)$, was then dissolved in $CH_2Cl_2$ (65 mL) and $H_2O_2$-urea (1.63 g, 17.3 mmol, 10.0 equiv) was added. The suspension was stirred vigorously in darkness for 6 h, after which an aliquot was removed to confirm complete consumption of $(PPh_3)_2Pt(CO_3)$ via $^{31}P\{^1H\}$ NMR. The suspension was filtered, and the solvent was removed to yield a yellow solid. The yellow solid was washed with benzene (2×10 mL), distilled water (2×10 mL), THF (−78 °C, 1×10 mL), and diethyl ether (2×10 mL). The remaining solid was dried under dynamic vacuum to yield a very slightly yellow powder (1.10 g, 69%). Yellow cubic crystals for X-ray diffraction were grown by vapor diffusion of diethyl ether into a $CH_2Cl_2$ solution of 1 at 4 °C.

Elemental Analysis: Calcd. for $C_{37}H_{30}O_4P_2Pt$+0.25($CH_2Cl_2$): C 54.77, H 3.76. Found: C 54.78, H 3.75.

$^1H$ NMR (400 MHz, $CDCl_3$) δ (ppm): 7.46 (m, 1H), 7.36 (m, 2H), 7.20 (m, 2H).

$^{13}C\{^1H\}$ NMR (151 MHz, $CDCl_3$) δ (ppm): 168.6 (dd, $J_{C-P}$ = 5.8 Hz; 3.3 Hz), 134.6 (d, $J_{C-P}$ = 11 Hz), 134.4 (d, $J_{C-P}$ = 11 Hz), 131.6 (d, $J_{C-P}$ = 2.7 Hz), 131.3 (d, $J_{C-P}$ = 2.7 Hz), 128.5 (d, $J_{C-P}$ = 7.7 Hz), 128.4 (d, $J_{C-P}$ = 8.1 Hz), 128.2 ($J_{C-P}$ = 62 Hz), 126.4 (d, $J_{C-P}$ = 65 Hz).

$^{31}P\{^1H\}$ NMR (162 MHz, $CDCl_3$) δ (ppm): 11.35 (d w/ $^{195}Pt$ satellites, $J_{P-P}$ = 25 Hz; $J_{P-Pt}$ = 3851 Hz, 1P), 5.94 (d w/ $^{195}Pt$ satellites, $J_{P-P}$ = 25 Hz; $J_{P-Pt}$ = 3282 Hz, 1P).

$^{195}$Pt NMR (86 MHz, $CDCl_3$) δ (ppm): −3949 (dd, $J_{Pt-P}$ = 3837 Hz; 3281 Hz).

FT-IR (solid, cm$^{-1}$): 1681, 1481, 1435, 1249, 1096, 980, 918, 779, 744, 729, 688, 554, 527.

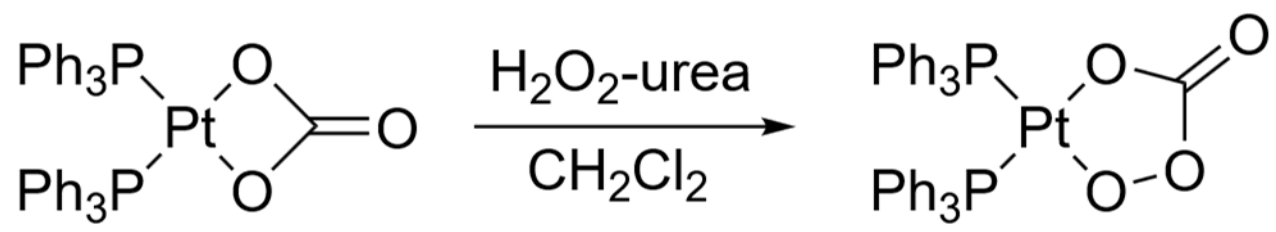


**$Pt(PPh_3)_2(CO_4)$ (1) or $Pt(PPh_3)_2(^{13}CO_4)$ from Pt(II)**. Reaction was carried out in ambient conditions and protected from light. $Pt(PPh_3)_2(CO_3)$ (80 mg, 0.10 mmol, 1.0 equiv) was dissolved in $CH_2Cl_2$ (4 mL). $H_2O_2$-urea (97 mg, 1.0 mmol, 10 equiv) was added and the suspension was stirred vigorously in darkness for 6 h, after which an aliquot was removed to confirm complete consumption of $(PPh_3)_2Pt(CO_3)$ via $^{31}P\{^1H\}$ NMR. The suspension was filtered through celite, and the solvent was removed to yield a yellow solid. The yellow solid was washed with benzene (1×5 mL), distilled water (1×5 mL), THF (−78°C, 1×5 mL), and diethyl ether (2×5 mL). The remaining solid was dried under dynamic vacuum to yield a very slightly yellow powder (48 mg, 59%).

Characterization of $Pt(PPh_3)_2(CO_4)$ (1) is consistent with preparation from Pt(0) starting material.

FT-IR ($^{13}$C labeled) (solid, cm$^{-1}$): 1637, 1481, 1435, 1221, 1096, 976, 746, 744, 698, 690, 555, 527.

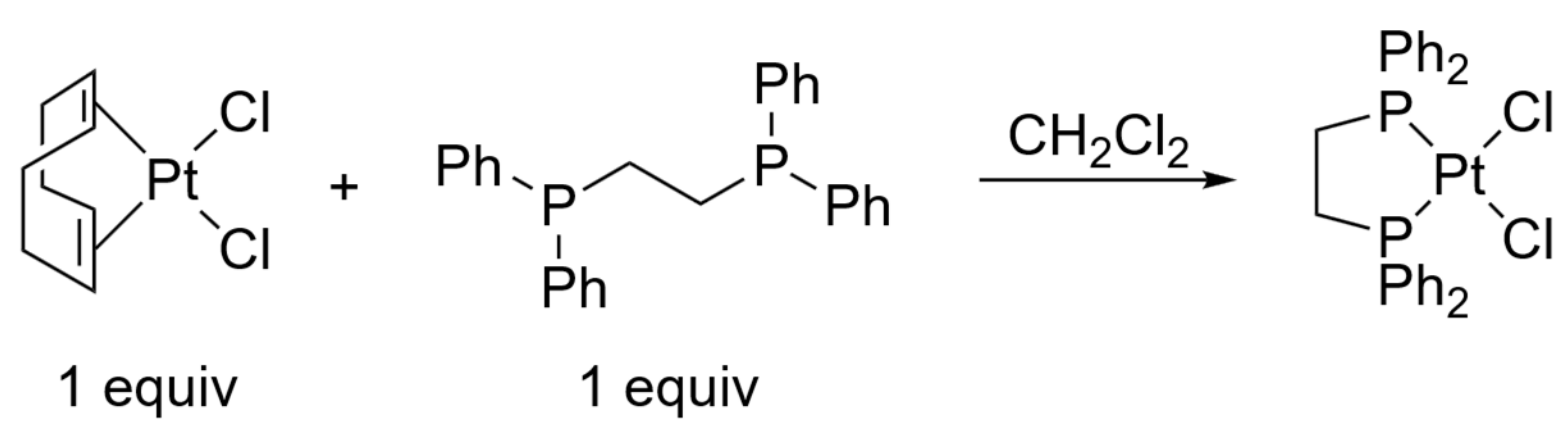


**Pt(dppe)$Cl_2$**. Reaction was carried out in ambient conditions following a literature reported procedure.[31] Pt(cod)$Cl_2$ (600 mg, 1.60 mmol, 1.00 equiv) was dissolved in $CH_2Cl_2$ (9.6 mL) in a 20 mL scintillation vial, followed by the addition of 1,2-bis(diphenylphosphino)ethane (640 mg, 1.60 mmol, 1.00 equiv). The resultant suspension was stirred for 15 min, after which hexanes (23 mL) was added. The reaction was transferred to a −20 °C freezer for ~4 h, after which the yellow solution was decanted from the white precipitate. The precipitate was washed with pentane (2×20 mL) before drying under vacuum. The product was collected as a white powder (1.03 g, 97%).

Characterization is consistent with previous literature report.[31]

$^1$H NMR (400 MHz, $CDCl_3$) δ (ppm): 7.86 (m, 2H), 7.49 (m, 3H), 2.34 (d, $J_{H-P}$ = 18 Hz, 1H).

$^{31}$P NMR{$^1$H} (162 MHz, $CDCl_3$) δ (ppm): 41.79 (s w/ $^{195}$Pt satellites, $J_{P-Pt}$ = 3623 Hz).

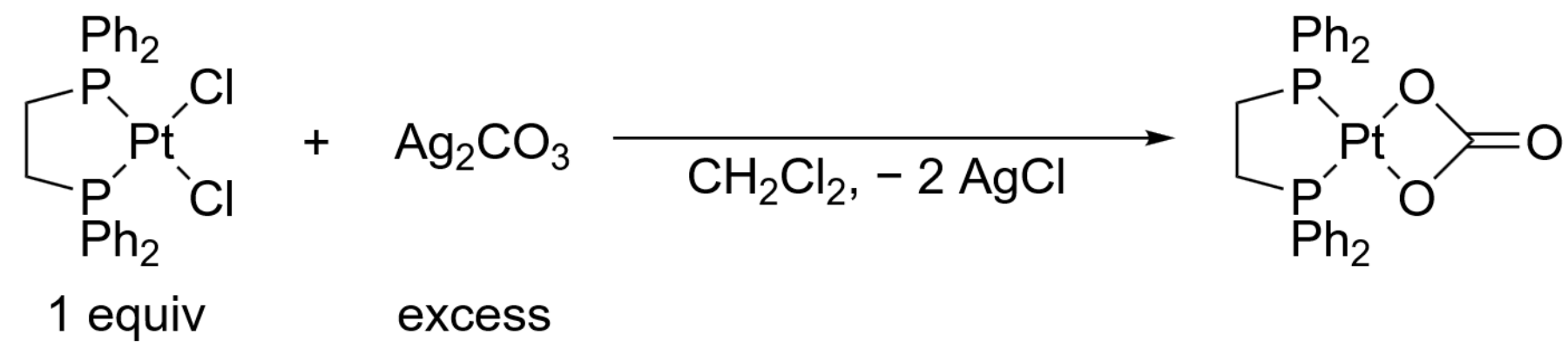


**Pt(dppe)(CO$_3$).** Reaction was carried out in ambient conditions and protected from light, following a modified literature reported procedure.[32] Pt(dppe)Cl$_2$ (1.03 g, 1.55 mmol, 1.00 equiv) was dissolved in CH$_2$Cl$_2$ (200 mL) in a 250 mL round-bottom-flask. Ag$_2$CO$_3$ (1.28 g, 4.64 mmol, 3.00 equiv) was added, followed by distilled water (0.5 mL). The suspension was stirred for 5 h, after which an aliquot was removed for $^{31}$P{$^1$H} NMR analysis to confirm full conversion. The suspension was filtered through celite to remove AgCl and unreacted Ag$_2$CO$_3$. Solvent was removed from the filtrate until ~10 mL remained, out of which the product had crystallized as a white solid. The suspension was filtered, and the light-yellow filtrate was discarded. The solid was washed with hexanes (3×10 mL) before drying under vacuum, yielding a fluffy white solid (972 mg, 96%). Long, colorless needles for SC-XRD were grown by vapor diffusion of diethyl ether into a CH$_2$Cl$_2$ solution of Pt(dppe)(CO$_3$) at 4 °C.

Characterization is consistent with previous literature report.[32]

$^1$H NMR (400 MHz, CDCl$_3$) δ (ppm): 7.86 (m, 2H), 7.45 (m, 3H), 2.48 (d, $J_{\mathrm{H-P}}$ = 16 Hz, 1H).

$^{13}$C{$^1$H} NMR (151 MHz, CDCl$_3$) δ (ppm): 168.9 (t, $J_{\mathrm{C-P}}$ = 3.9 Hz), 132.6 (t, $J_{\mathrm{C-P}}$ = 5.6 Hz), 132.3 (s), 129.6 (t, $J_{\mathrm{C-P}}$ = 5.7 Hz), 128.0 (dd, $J_{\mathrm{C-P}}$ = 64 Hz; 2.8 Hz), 26.2 (dd, $J_{\mathrm{C-P}}$ = 53 Hz; 3.8 Hz).

$^{31}$P{$^1$H} NMR (162 MHz, CDCl$_3$) δ (ppm): 32.59 (s w/ $^{195}$Pt satellites, $J_{\mathrm{P-Pt}}$ = 3542 Hz).

FT-IR (solid, cm$^{-1}$): 1675, 1625, 1481, 1436, 1183, 1102, 980, 829, 814, 751, 717, 707, 687, 533.

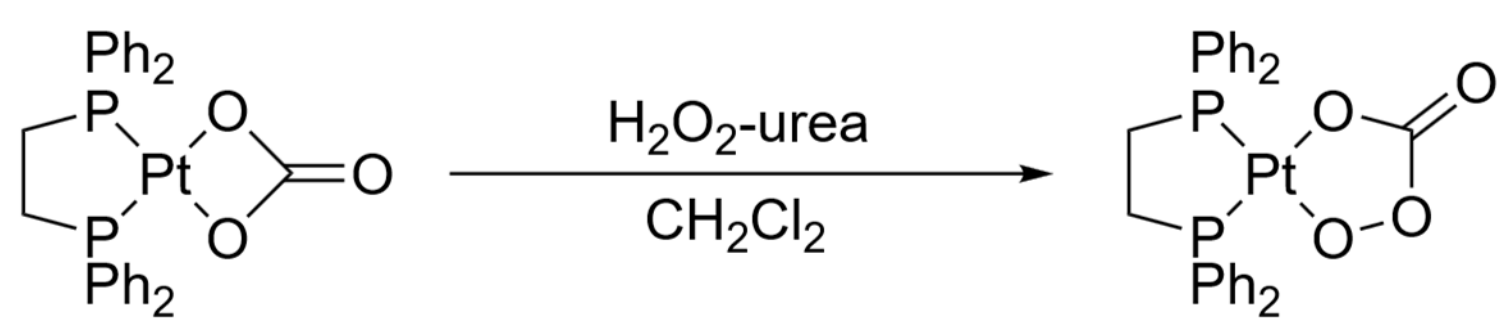


**Pt(dppe)(CO$_4$) (2)**. Reaction was carried out in ambient conditions and protected from light. Pt(dppe)(CO$_3$) (972 mg, 1.49 mmol, 1.00 equiv) was dissolved in CH$_2$Cl$_2$ (82 mL). H$_2$O$_2$-urea (210 mg, 2.23 mmol, 1.50 equiv) was added and the suspension was stirred vigorously in darkness for 2 h, after which an aliquot was removed to confirm complete consumption of Pt(dppe)(CO$_3$) via $^{31}$P{$^1$H} NMR. The suspension was filtered through celite, and the solvent was removed until ca. 5 mL of orange solution remained. The solution placed in a refrigerator overnight for crystallization. The following day, the orange supernatant was decanted leaving the product as yellow crystals which were washed with cold DCM (−78 °C, 1×2 mL), water (1×5 mL), THF (2×10 mL), and diethyl ether (1×10 mL). The crystals were dried under dynamic vacuum to yield a yellow solid (547 mg, 55%). Yellow cubic crystals for X-ray diffraction were grown by vapor diffusion of diethyl ether into a CH$_2$Cl$_2$ solution of **2** at 4 °C.

Elemental Analysis: Calcd. for C$_{27}$H$_{24}$O$_4$P$_2$Pt: C 48.88, H 3.61. Found: C 48.07, H 3.62.

$^1$H NMR (400 MHz, CDCl$_3$) δ (ppm): 7.83 (m, 2H), 7.48 (m, 3H), 2.44 (m, 1H).

$^{13}C\{^1H\}$ NMR (151 MHz, $CD_2Cl_2$) δ (ppm): 168.3 (dd, $J_{C-P}$ = 4.1 Hz; 3.2 Hz), 133.3 (d, $J_{C-P}$ = 11 Hz), 133.2 (d, $J_{C-P}$ = 11 Hz), 132.9 (d, $J_{C-P}$ = 2.8 Hz), 132.8 (d, $J_{C-P}$ = 2.8 Hz), 129.94 (s), 129.86 (s), 127.99 (d, $J_{C-P}$ = 61 Hz), 127.57 ($J_{C-P}$ = 64 Hz), 27.13 (dd, $J_{C-P}$ = 45 Hz; 8.8 Hz), 26.58 (dd, $J_{C-P}$ = 43 Hz; 7.4 Hz).

$^{31}P\{^1H\}$ NMR (162 MHz, $CDCl_3$) δ (ppm): 31.86 (d w/ $^{195}Pt$ satellites, $J_{P-P}$ = 12 Hz; $J_{P-Pt}$ = 3165 Hz, 1P), 29.27 (d w/ $^{195}Pt$ satellites, $J_{P-P}$ = 12 Hz; $J_{P-Pt}$ = 3676 Hz, 1P).

FT-IR (solid, $cm^{-1}$): 1668, 1640, 1479, 1435, 1249, 1105, 982, 823, 779, 707, 687, 531.

## A.3. Instrumentation

Nuclear magnetic resonance (NMR) spectra were acquired in the Harvard University Department of Chemistry and Chemical Biology Laukien-Purcell Instrumentation Center (LPIC). Either a Bruker AVANCE NEO 400 MHz spectrometer, a JEOL ECZ400s 400 MHz spectrometer equipped with a broadband probe, or a Bruker AVANCE NEO 600 MHz spectrometer was used to acquire NMR spectra. Chemical shifts (δ) are reported in parts per million (ppm). $^1H$ and $^{13}C$ spectra referenced using the residual protio solvent peaks as internal standards, $^1H$ NMR ($CHCl_3$: $\delta$ 7.26; $CHDCl_2$: $\delta$ 5.32) and $^{13}C$ NMR ($CDCl_3$: $\delta$ 77.16; $CD_2Cl_2$: $\delta$ 54.00). $^{31}P$ NMR were collected in either deuterated or protic (unlocked) solvents and were referenced to 85% $H_3PO_4$ ($\delta$ 0.00). Multiplicities are indicated as singlets (s), doublets (d), triplets (t), multiplets (m), or combinations thereof. *MestreNova 12.0.3* software was used for NMR data processing and analysis.

Solid-state Fourier-transform infrared spectroscopy (FTIR) was collected in the LPIC on powdered samples in ambient conditions using a Bruker ALPHA FT-IR spectrometer. Solution-state FTIR measurements were performed with a Bruker INVENIO FT-IR spectrometer in continuous scan mode using a liquid $N_2$ cooled MCT detector. Samples were prepared in an $N_2$-filled glovebox and were injected into an IR cell (BaF2 windows, 500 μm spacing) that was sealed before removal from the glovebox. The FTIR spectrum for solvent background was subtracted from each recorded spectrum. Frequencies above 3900 $cm^{-1}$ were filtered out with a Ge filter placed after the sample stage. Bruker *OPUS 8.5* software was used for FTIR data processing and analysis.

Ultraviolet-visible-near infrared (UV-vis-NIR) absorption spectroscopy was performed using a Varian Cary 5000 UV-vis-NIR spectrophotometer. Unless otherwise noted, samples were prepared under anhydrous conditions in an $N_2$-filled glovebox and added to quartz cuvettes and capped with silicone rubber lined caps prior to removal from the glovebox. The appropriate solvent background was subtracted from each recorded spectrum.

Elemental analyses were obtained from Robertson Microlit Laboratories.

## A.4. Crystallography

Single-crystal X-ray diffraction (SCXRD) measurements were performed on single crystals coated with Paratone-N oil and mounted on MiTeGen loops. All SCXRD data were collected at the Harvard University Department of Chemistry and Chemical Biology X-ray Laboratory (X-ray Core) using Mo Kα radiation on a Bruker D8 Venture single crystal diffractometer equipped with a PHOTON III–C14 detector. Data were collected on either a fixed-chi or kappa goniometer. Crystals were frozen at 100 K during data collection using an Oxford Cryosystems Cryostream. During data collection, no significant crystal decay was observed. Pt(dppe)($CO_4$) was refined as a two-component merohedral twin based on the diffraction pattern. The twin law was found to be [1.0, –0.019, –0.001, 0.034, 0.999, –0.025, 0.002, 0.03, 1.0] with a minor fraction occupancy of 0.078(10). Raw data for all compounds were integrated and corrected for Lorentz and polarization effects using Bruker AXS SAINT software and corrected for absorption using SADABS.[33] For all structures, space

group assignments were determined by examination of systematic absences, *E*-statistics, and successive refinement of the structures. Structures were solved by intrinsic phasing using SHELXT and refined using SHELXL operated in the OLEX2 interface.[34–36] Thermal parameters were refined anisotropically for all non-hydrogen atoms. Hydrogen atoms were placed in ideal positions and refined using a riding model. Outlier reflections were omitted from the refinement when appropriate. Disorder refinement to model disorder on solvent molecules or ligands were required for $Pt(PPh_3)_2(CO_3)$, $Pt(PPh_3)_2(CO_4)$, $Pt(dppe)(CO_3)$, $Pt(dppe)(CO_4)$ and $Pt(dppeO)(Me_2SO)(CO_3)$. Disorder was refined using displacement parameter restraints and constraints (SIMU, RIGU, EADP) and bond length restraints (SADI) when necessary. Further information on refinement strategies is provided in the crystallographic information files. Additional crystallographic data are provided in **Tables S1–S5** and additional atomic displacement parameter plots are shown in **Figures S1–S5**.

## A.5. Computational Methods

Density functional theory (DFT) geometry optimizations and time-dependent DFT (TD-DFT) were performed using the *ORCA 6.0* software package.[37–39] Initial coordinates were generated from crystal-structures or modifications thereof. Optimized geometries were calculated using a BP86 functional,[40–42] with a def2-SVP basis set for C/H atoms and def2-TZVP basis set for all other atoms.[43] A zeroth-order regular approximation (ZORA) was applied to Pt.[44] TD-DFT for **2** was carried out using a BP86 functional with a def2-TZVP basis set on a truncated structure, $Pt(dmpe)(CO_4)$, where methyl groups replace phenyl rings of the dppe ligand. Acetonitrile or dichloromethane implicit solvation models were used.[45] Frequency calculations confirmed that all optimizations had converged to true minima on the potential energy surface (i.e., no imaginary frequencies). Geometry-optimized coordinates are listed in **Tables S6-S12**. TD-DFT results of the $S_n$ excited states are shown in **Table S13** and **Figure S27**. Geometry-optimized structures, calculated IR frequencies, and natural transition orbitals (NTOs) were visualized in *ChemCraft 1.8* Software. [46]

## A.6. Photochemical Methods

**NMR Photolyses.** In a nitrogen-filled glovebox, solutions at several concentrations of **1** or **2** in the specified solvent ($CH_2Cl_2$ or $CH_3CN$) were prepared with ca. 1 equiv of triphenyl phosphate as an internal standard. The exact amount of triphenyl phosphate used was noted and utilized in analyses. Solutions were used within two days of preparation and stored in a −40 °C freezer when not in use. 0.5 mL of each solution was loaded into an NMR tube, sealed under nitrogen, and subsequently the samples were removed from the glovebox for analysis. Irradiation was carried out using two 440 nm Kessil lamps placed 180° from each other, and each 1 inch from the sample. A fan was used to keep the sample at room temperature. $^{31}P\{^1H\}$ NMR spectra were collected after irradiation across several timepoints. The decay in integrations of **1** or **2** peaks, relative to the known concentration of internal standard, were used to track the decrease in [**1**] or [**2**] as a function of irradiation time. Power dependencies were carried out by adjusting the intensity setting of the Kessil lamps (100%, 75%, 50%). Control photolyses were conducted with $Pt(PPh_3)_2(CO_3)$ and $Pt(dppe)(CO_3)$ for which no reaction was observed.

**FTIR Photolyses.** In a nitrogen-filled glovebox, 5 mM solutions of **1** or **2** in the specified solvent ($CD_2Cl_2$, $CDCl_3$, or $CD_3CN$) were prepared. The solutions were filtered through 0.2 μM syringe filters directly into the air-tight IR cell ($BaF_2$ windows, 500 μm spacing). The cell was sealed and removed from the glovebox. Irradiation was carried out using a single 440 nm Kessil lamp with a collimating lens. IR spectra were collected at various timepoints.

**Quantum Yield Measurements.** Photochemical quantum yield measurements were performed by using a nanosecond pulsed laser system as the excitation light source ($\lambda_{exc}$ = 440 nm). Excitation pulses were furnished by a 50 Hz diode-pumped Nd:YAG laser system with an integrated OPO (NT230-50, Ekspla) achieving an average pulse width of ca. 4 ns and per pulse energies of ca. 13 mJ at 440 nm. The generated vertically polarized OPO output was sent through a 70(R)/30(T) beam-splitter (BST10, Thorlabs), re-sized using an off-axis parabolic mirror (f = 75 mm, MPD149-F01, Thorlabs), and re-collimated with a UV-fused silica plano-convex lens (f = 50 mm, LA4148-UV-ML, Thorlabs) to achieve a beam diameter of ca. 3 mm. The re-sized laser pulse was attenuated by absorptive neutral density filters to achieve an energy of ca. 900 mJ per pulse at the sample position. Average per-pulse energies were measured with an energy sensor (J-25MB-LE, Coherent).

The average per-pulse energy was measured across 5000 pulses through a $CH_2Cl_2$ blank, and then through samples of **2** (19 mM, $CH_2Cl_2$). The difference in photons absorbed between the blank and each sample was taken as photons absorbed by the sample. The loss in absorption as [**2**] decreased and competitive absorption by the photoproduct at the excitation wavelength are sources of error, though they are minimized at low conversion. This error leads to an underestimation of the intrinsic quantum yield. $^{31}P\{^1H\}$ spectra were collected before and after the photolysis to quantify conversion (4%) and obtain a quantum yield. This experiment was carried out on three different samples to quantify error.

## B. X-Ray Crystallography

### B.1. $Pt(PPh_3)_2(CO_3)$ and 1

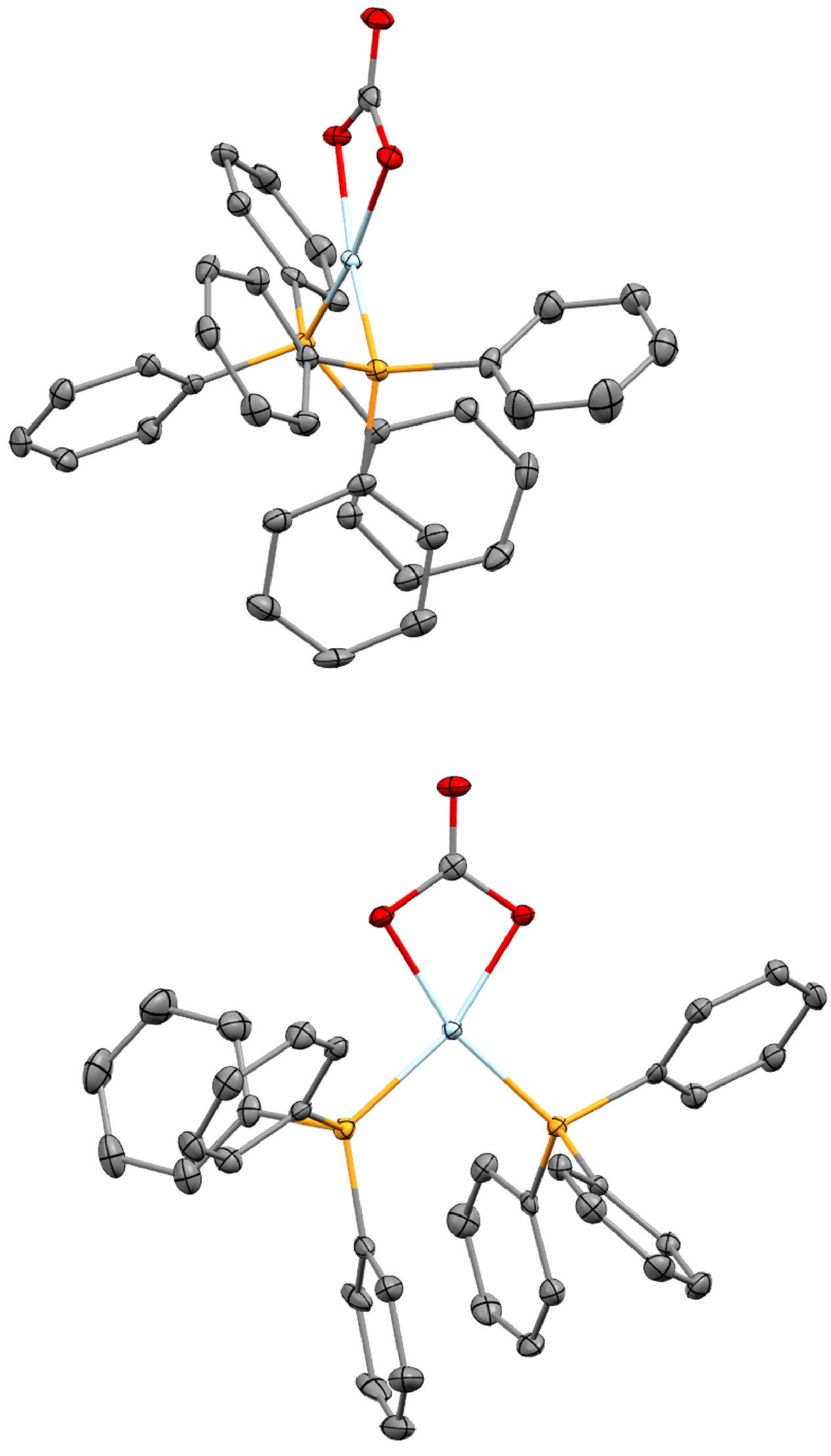

**Figure S1.** Crystal structure of $Pt(PPh_3)_2(CO_3)$. Two disorder-free molecules of $Pt(PPh_3)_2(CO_3)$ are present in the asymmetric unit. Hydrogen atoms and two solvent molecules ($CH_2Cl_2$) are omitted for clarity. Atomic displacement parameters set at 50% probability level. Color scheme: C, gray; O, red; P, orange; Pt, light blue.

**Table S1**. Crystallographic data for $Pt(PPh_3)_2(CO_3)$.

| | |
|---|---|
| Compound | $Pt(PPh_3)_2(CO_3)$ |
| Formula | $C_{38}H_{32}Cl_2O_3P_2Pt$ |
| *T* (K) | 100.00 |
| Crystal System | Monoclinic |
| Space Group | $P2_1/c$ |
| *a* (Å)<br>*b* (Å)<br>*c* (Å) | 23.5247(9)<br>15.2998(6)<br>19.7375(8) |
| *α* (°)<br>*β* (°)<br>*γ* (°) | 90<br>105.3640(10)<br>90 |
| *V* (Å$^3$) | 6850.1(5) |
| Z | 8 |
| Radiation<br>*λ* (Å) | Mo Kα<br>0.71073 |
| 2*Θ* Range for Data Collection (°) | 4.114 to 50.086 |
| Completeness to 2*Θ* | 99.8%<br>(2*Θ* = 50.086°) |
| Data / Restraints / Parameters | 12106 / 6 / 849 |
| Goodness of Fit on $F^2$ | 1.014 |
| $R_1$*<br>$wR_2$† ($I>2\sigma(I)$) | 0.0204<br>0.0342 |
| $R_1$* (all data)<br>$wR_2$† (all data) | 0.0367<br>0.0404 |
| Largest Diff. Peak and Hole (e Å$^{-3}$) | 0.786 and –0.508 |

*$R_1 = \sum||F_o| - |F_c||/\sum|F_o|$. †$wR_2 = \{\sum[w(F_o^2 - F_c^2)^2]/\sum[w(F_o^2)^2]\}^{1/2}$.

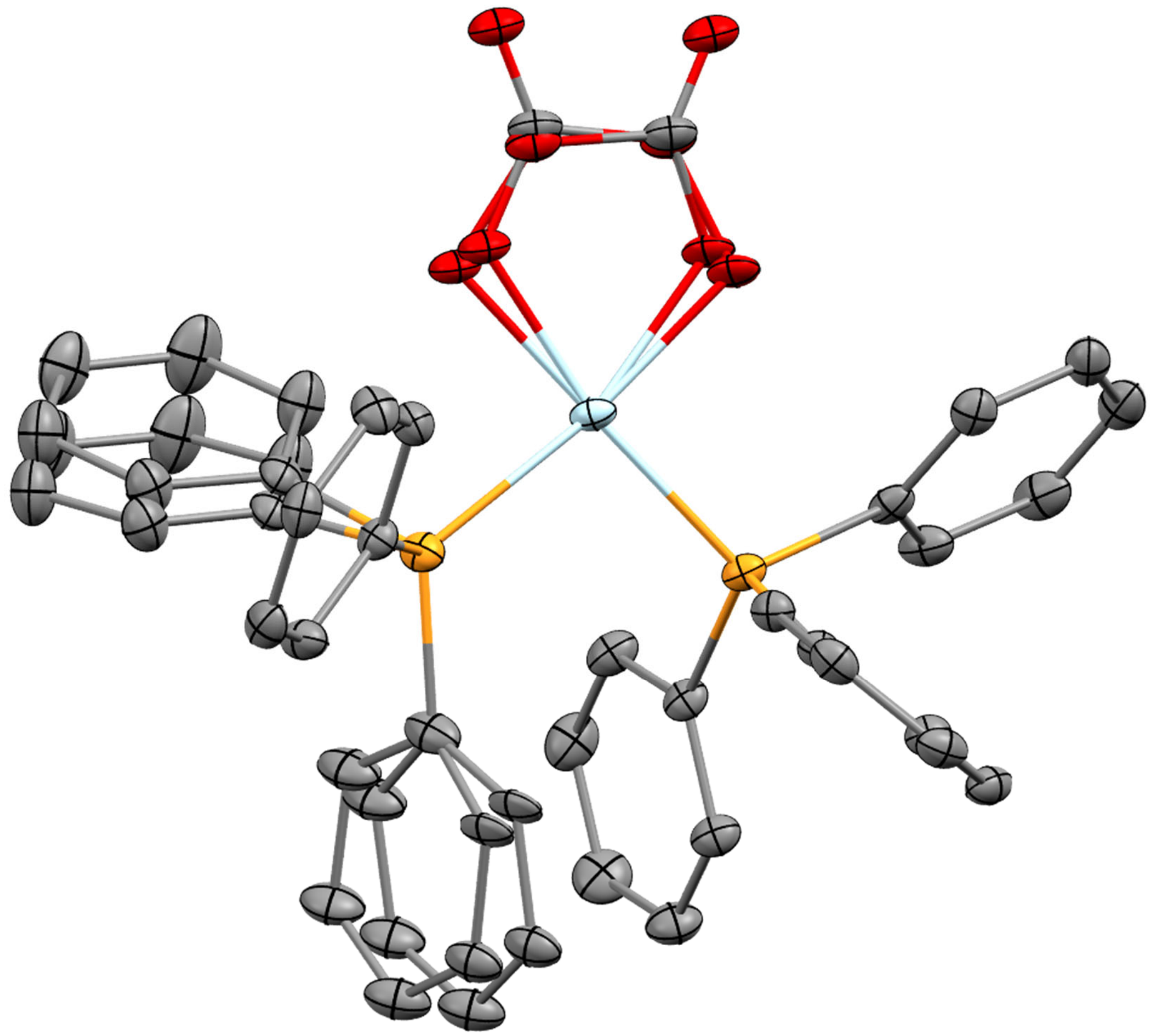

**Figure S2**. Crystal structure of **1**. Hydrogen atoms and one solvent molecule ($CH_2Cl_2$) are omitted for clarity. Disorder modeling is shown, including two-part disorder of the $CO_4$ ligand and two-part disorder of two phenyl rings of $PPh_3$. Atomic displacement parameters set at 30% probability level. Color scheme: C, gray; O, red; P, orange; Pt, light blue.

**Table S2**. Crystallographic data for **1**.

| Compound | $Pt(PPh_3)_2(CO_4)$ |
|---|---|
| Formula | $C_{38}H_{32}Cl_2O_4P_2Pt$ |
| $T$ (K) | 100.00 |
| Crystal System | Triclinic |
| Space Group | $P\bar{1}$ |
| $a$ (Å)<br>$b$ (Å)<br>$c$ (Å) | 10.7823(4)<br>12.3567(4)<br>15.5957(5) |
| $\alpha$ (°)<br>$\beta$ (°)<br>$\gamma$ (°) | 80.8120(10)<br>70.6240(10)<br>68.7220(10) |
| $V$ (Å$^3$) | 1824.74(11) |
| Z | 2 |
| Radiation<br>$\lambda$ (Å) | Mo Kα<br>0.71073 |
| $2\Theta$ Range for Data Collection (°) | 4.246 to 50.088 |
| Completeness to $2\Theta$ | 99.9%<br>($2\Theta$ = 50.088°) |
| Data / Restraints / Parameters | 6452 / 527 / 560 |
| Goodness of Fit on $F^2$ | 1.155 |
| $R_1$*<br>w$R_2$† ($I$>2$\sigma$($I$)) | 0.0251<br>0.0575 |
| $R_1$* (all data)<br>w$R_2$† (all data) | 0.0271<br>0.0586 |
| Largest Diff. Peak and Hole (e Å$^{-3}$) | 0.824 and −0.877 |

*$R_1 = \sum||F_o| - |F_c||/\sum|F_o|$. †$wR_2 = \{\sum[w(F_o^2 - F_c^2)^2]/\sum[w(F_o^2)^2]\}^{1/2}$.

## B.2. Pt(dppe)($CO_3$) and 2

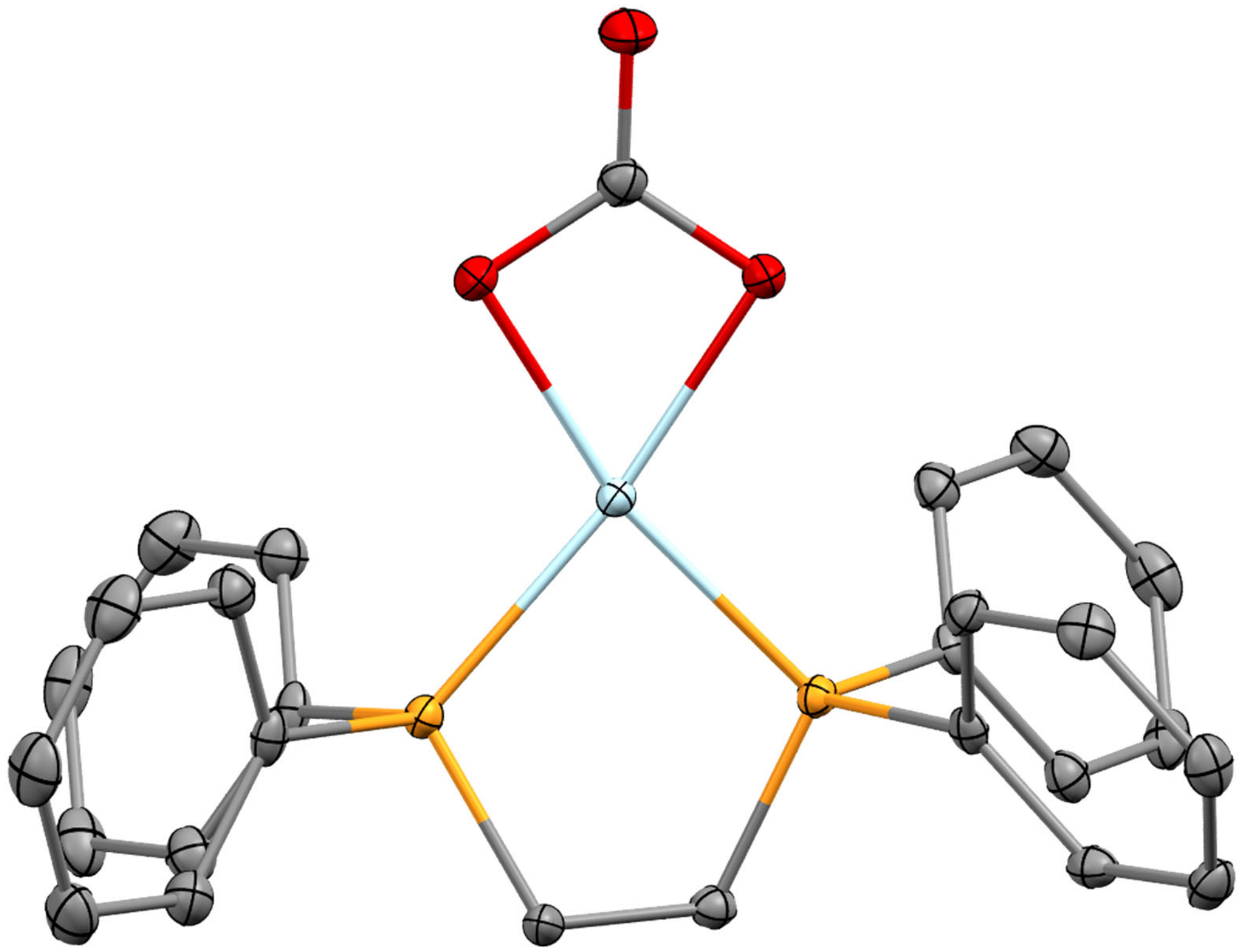

**Figure S3.** Crystal structure of Pt(dppe)($CO_3$). Hydrogen atoms and one solvent molecule ($CH_2Cl_2$) are omitted for clarity. Atomic displacement parameters set at 50% probability level. Color scheme: C, gray; O, red; P, orange; Pt, light blue.

**Table S3**. Crystallographic data for Pt(dppe)($CO_3$).

| Compound | Pt(dppe)($CO_3$) |
|---|---|
| Formula | $C_{28}H_{26}Cl_2O_3P_2Pt$ |
| $T$ (K) | 100.00 |
| Crystal System | Monoclinic |
| Space Group | $C2/c$ |
| $a$ (Å)<br>$b$ (Å)<br>$c$ (Å) | 28.3784(6)<br>11.1623(2)<br>17.2398(4) |
| $\alpha$ (°)<br>$\beta$ (°)<br>$\gamma$ (°) | 90<br>98.9305(7)<br>90 |
| $V$ (Å$^3$) | 5394.8(2) |
| Z | 8 |
| Radiation<br>$\lambda$ (Å) | Mo Kα<br>0.71073 |
| $2\Theta$ Range for Data Collection (°) | 4.480 to 50.080 |
| Completeness to $2\Theta$ | 99.9%<br>($2\Theta$ = 50.080°) |
| Data / Restraints / Parameters | 4775 / 3 / 335 |
| Goodness of Fit on $F^2$ | 1.065 |
| $R_1$*<br>w$R_2$† ($I$>2$\sigma$($I$)) | 0.0143<br>0.0277 |
| $R_1$* (all data)<br>w$R_2$† (all data) | 0.0170<br>0.0287 |
| Largest Diff. Peak and Hole (e Å$^{-3}$) | 0.372 and −0.401 |

*$R_1 = \sum||F_o| - |F_c||/\sum|F_o|$. †$wR_2 = \{\sum[w(F_o^2 - F_c^2)^2]/\sum[w(F_o^2)^2]\}^{1/2}$.

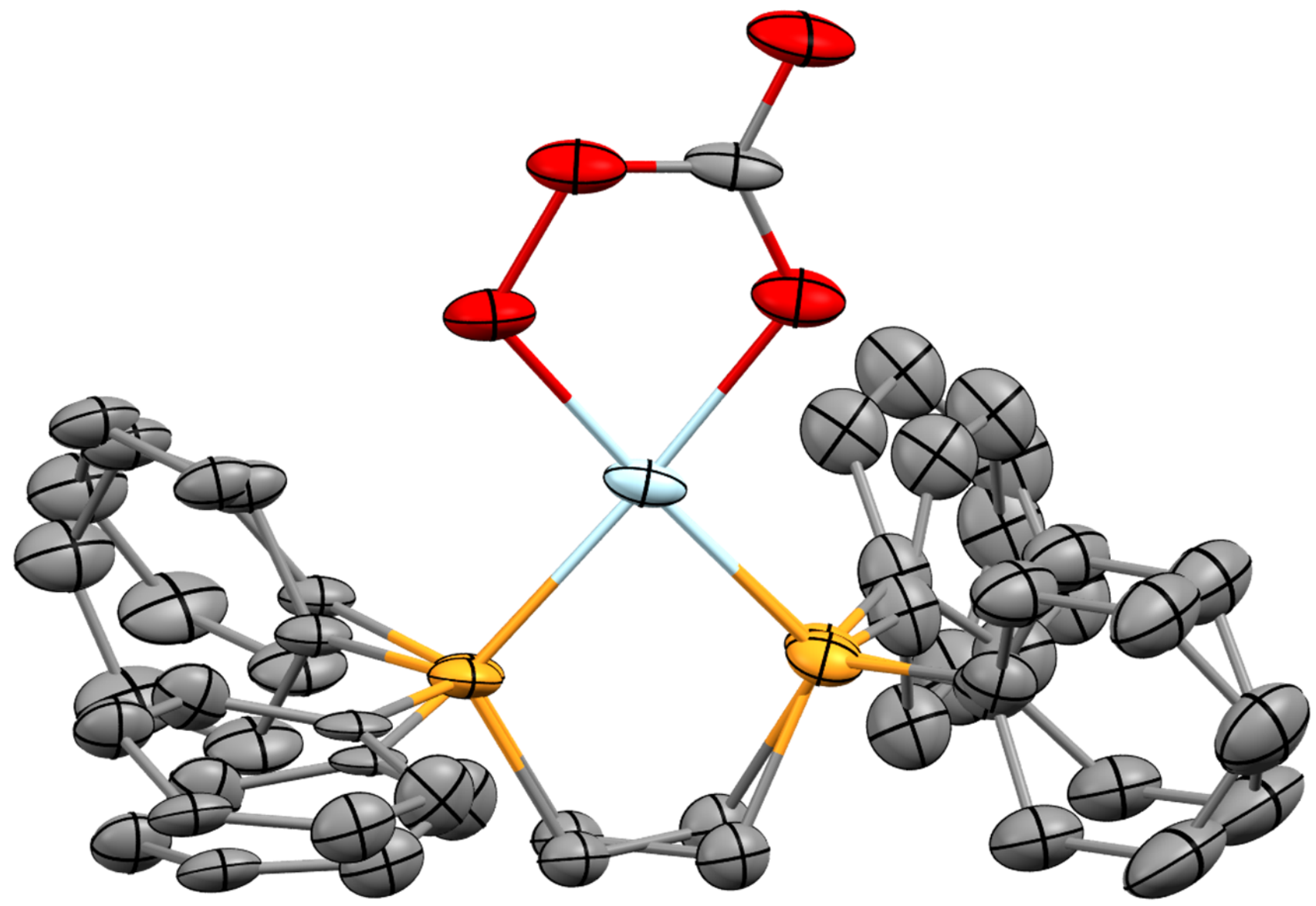

**Figure S4.** Crystal structure of **2**. Hydrogen atoms are omitted for clarity. Disorder modeling is shown, including two-part disorder of the dppe ligand (ethyl backbone and all phenyl rings). Atomic displacement parameters set at 30% probability level. Color scheme: C, gray; O, red; P, orange; Pt, light blue.

**Table S4**. Crystallographic data for **2**.

| Compound | Pt(dppe)($CO_4$) |
|---|---|
| Formula | $C_{27}H_{24}O_4P_2Pt$ |
| $T$ (K) | 100.00 |
| Crystal System | Monoclinic |
| Space Group | $P2_1/n$ |
| $a$ (Å)<br>$b$ (Å)<br>$c$ (Å) | 10.3467(7)<br>15.4999(9)<br>15.3983(10) |
| $\alpha$ (°)<br>$\beta$ (°)<br>$\gamma$ (°) | 90<br>102.512(2)<br>90 |
| $V$ (Å$^3$) | 2410.8(3) |
| Z | 4 |
| Radiation<br>$\lambda$ (Å) | Mo Kα<br>0.71073 |
| 2$\Theta$ Range for Data Collection (°) | 2.628 to 50.340 |
| Completeness to 2$\Theta$ | 99.4%<br>(2$\Theta$ = 50.340°) |
| Data / Restraints / Parameters | 4338 / 836 / 393 |
| Goodness of Fit on $F^2$ | 1.006 |
| $R_1$*<br>w$R_2$† ($I$>2$\sigma$($I$)) | 0.0951<br>0.1883 |
| $R_1$* (all data)<br>w$R_2$† (all data) | 0.0999<br>0.1911 |
| Largest Diff. Peak and Hole (e Å$^{-3}$) | 5.035 and −3.106 |

*$R_1 = \sum||F_o| - |F_c||/\sum|F_o|$. †$wR_2 = \{\sum[w(F_o^2 - F_c^2)^2]/\sum[w(F_o^2)^2]\}^{1/2}$.

### B.3. Pt(dppeO)(PhSMe)($CO_3$) Photoproducts

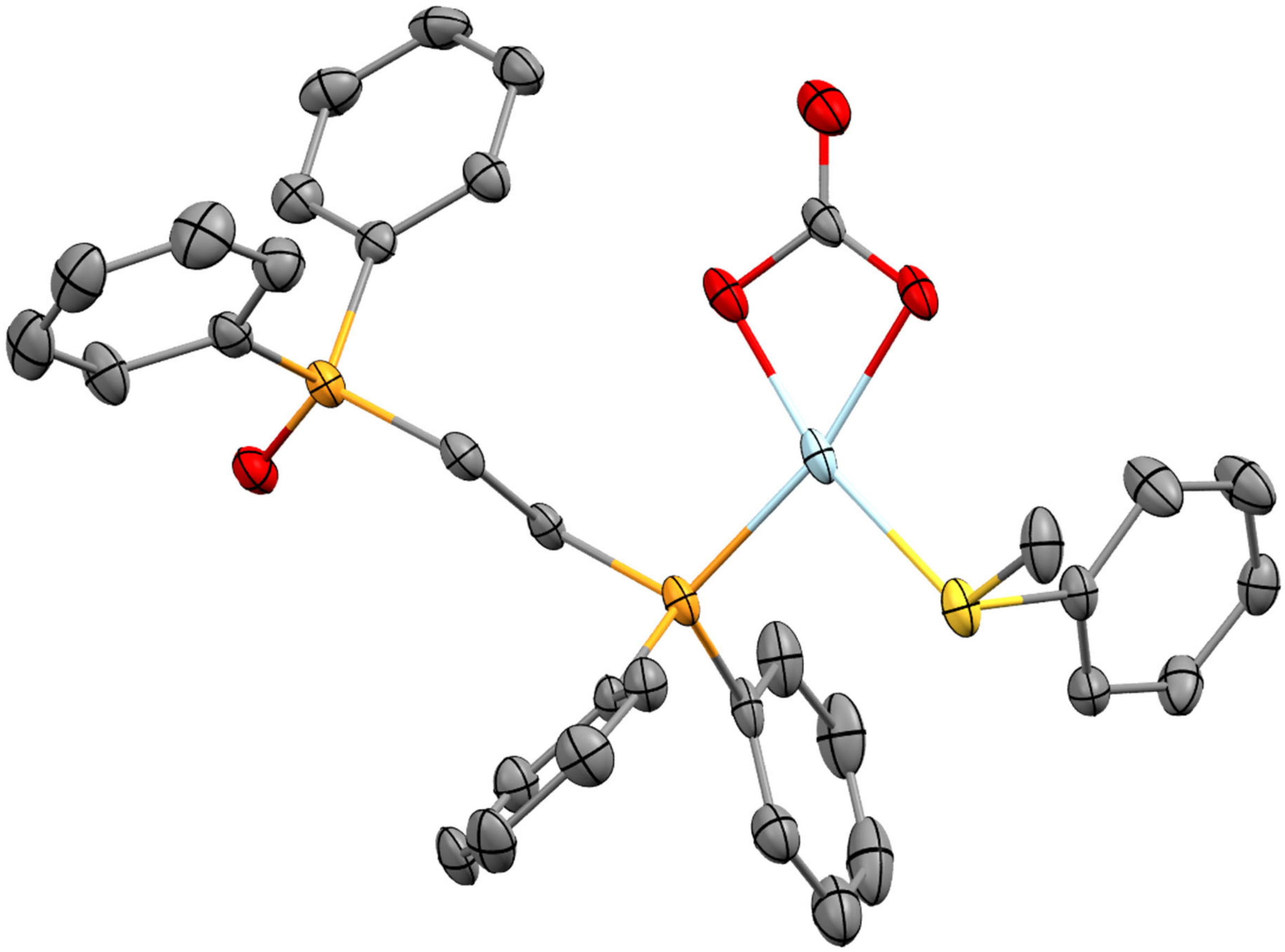

**Figure S5.** Crystal structure of Pt(dppeO)(PhSMe)($CO_3$). Hydrogen atoms are omitted for clarity. Atomic displacement parameters set at 50% probability level. Color scheme: C, gray; O, red; P, orange; S, yellow; Pt, light blue.

**Table S5**. Crystallographic data for Pt(dppeO)(PhSMe)($CO_3$).

| Compound | Pt(dppeO)(PhSMe)($CO_3$) |
|---|---|
| Formula | $C_{34}H_{32}O_4P_2PtS$ |
| $T$ (K) | 100.00 |
| Crystal System | Triclinic |
| Space Group | $P\bar{1}$ |
| $a$ (Å)<br>$b$ (Å)<br>$c$ (Å) | 12.1555(6)<br>12.1565(6)<br>12.5314(7) |
| $\alpha$ (°)<br>$\beta$ (°)<br>$\gamma$ (°) | 61.490(2)<br>73.761(2)<br>76.714(2) |
| $V$ (Å$^3$) | 1552.05(14) |
| Z | 2 |
| Radiation<br>$\lambda$ (Å) | Mo Kα<br>0.71073 |
| 2$\Theta$ Range for Data Collection (°) | 3.838 to 50.244 |
| Completeness to 2$\Theta$ | 99.6 %<br>(2$\Theta$ = 50.244°) |
| Data / Restraints / Parameters | 5530 / 0 / 380 |
| Goodness of Fit on $F^2$ | 1.041 |
| $R_1$* <br>w$R_2$† ($I>2\sigma(I)$) | 0.0340<br>0.0768 |
| $R_1$*(all data)<br>w$R_2$† (all data) | 0.0401<br>0.0809 |
| Largest Diff. Peak and Hole (e Å$^{-3}$) | 3.214 and −2.154 |

*$R_1 = \sum||F_o| - |F_c||/\sum|F_o|$. †$wR_2 = \{\sum[w(F_o^2 - F_c^2)^2]/\sum[w(F_o^2)^2]\}^{1/2}$.

## C. Compound 1

### C.1. Spectroscopic Characterization

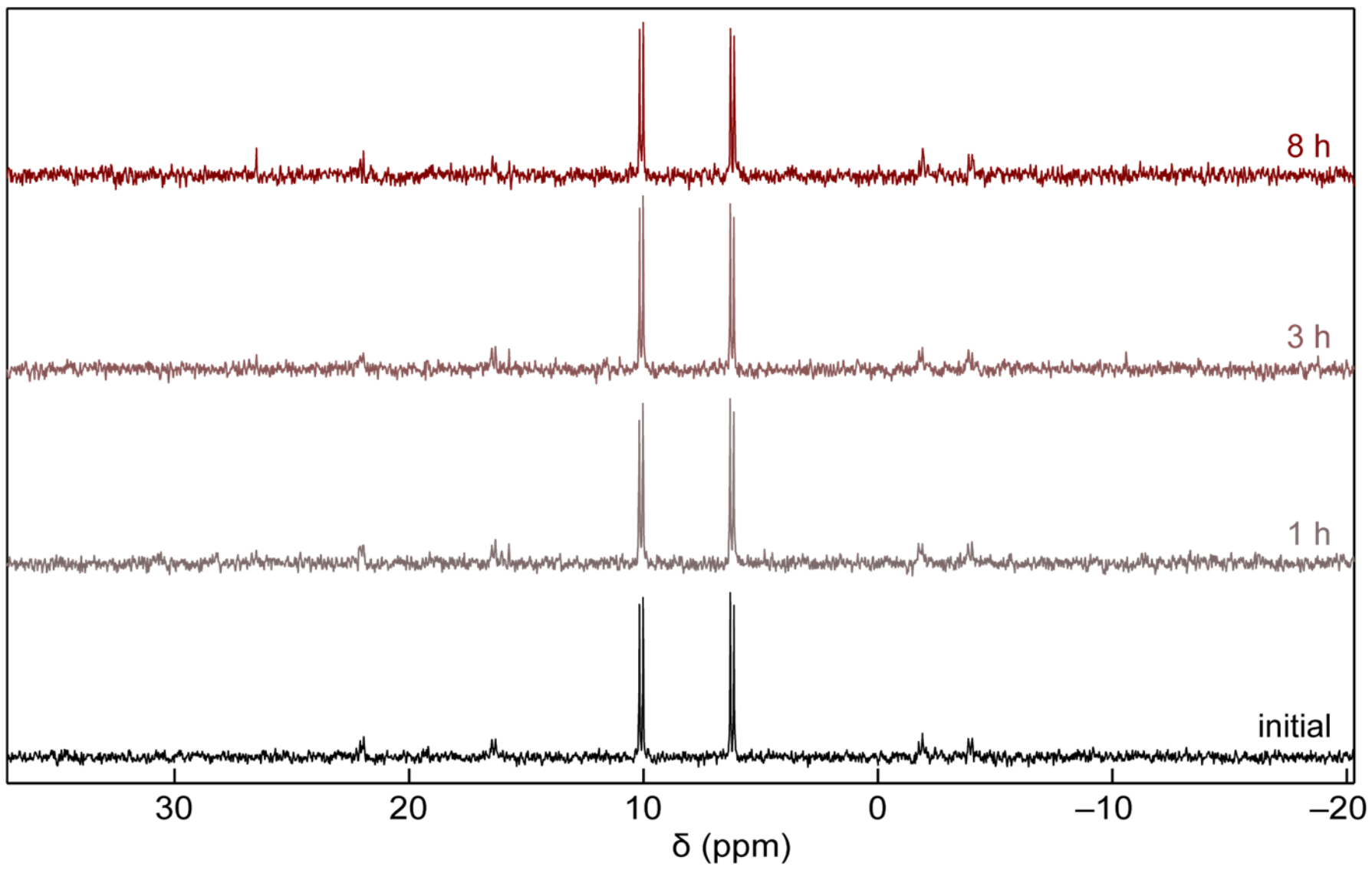


**Figure S6.** $^{31}P\{^1H\}$ NMR spectra of **1** (5 mM in $CH_3CN$) initially (bottom, black), and after 1, 3, and 8 h (top, red) at 50 °C.

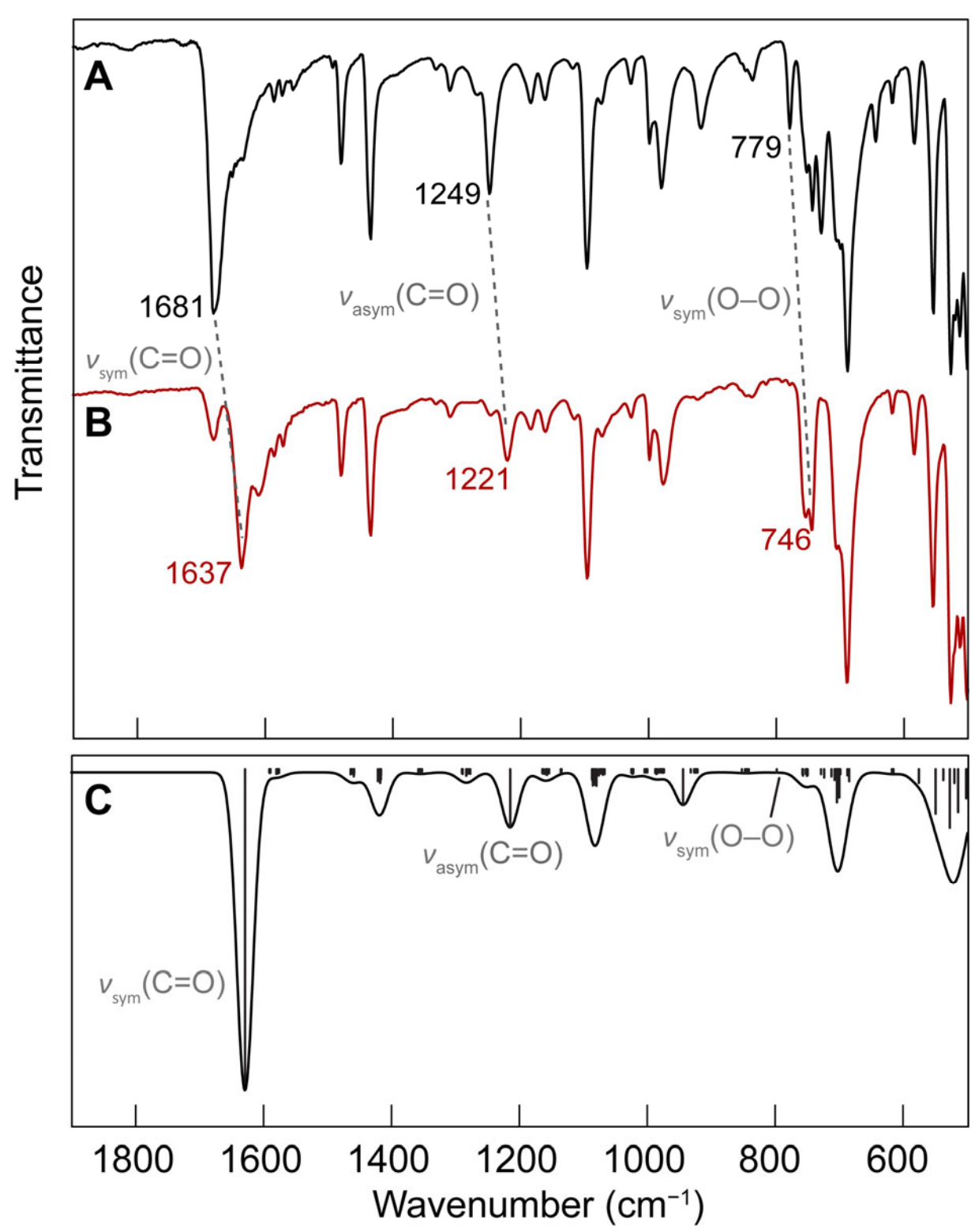


**Figure S7.** FT-IR spectra (solid-state) of (**A**) **1** (black) and (**B**) $^{13}C$-labeled **1** (red). Selected $^{13}C$ isotope shifts labeled (gray). (**C**) Calculated IR frequencies and resultant spectrum (black) for DFT-optimized geometry of **1** (**Table S6**) with $CH_2Cl_2$ implicit solvation.

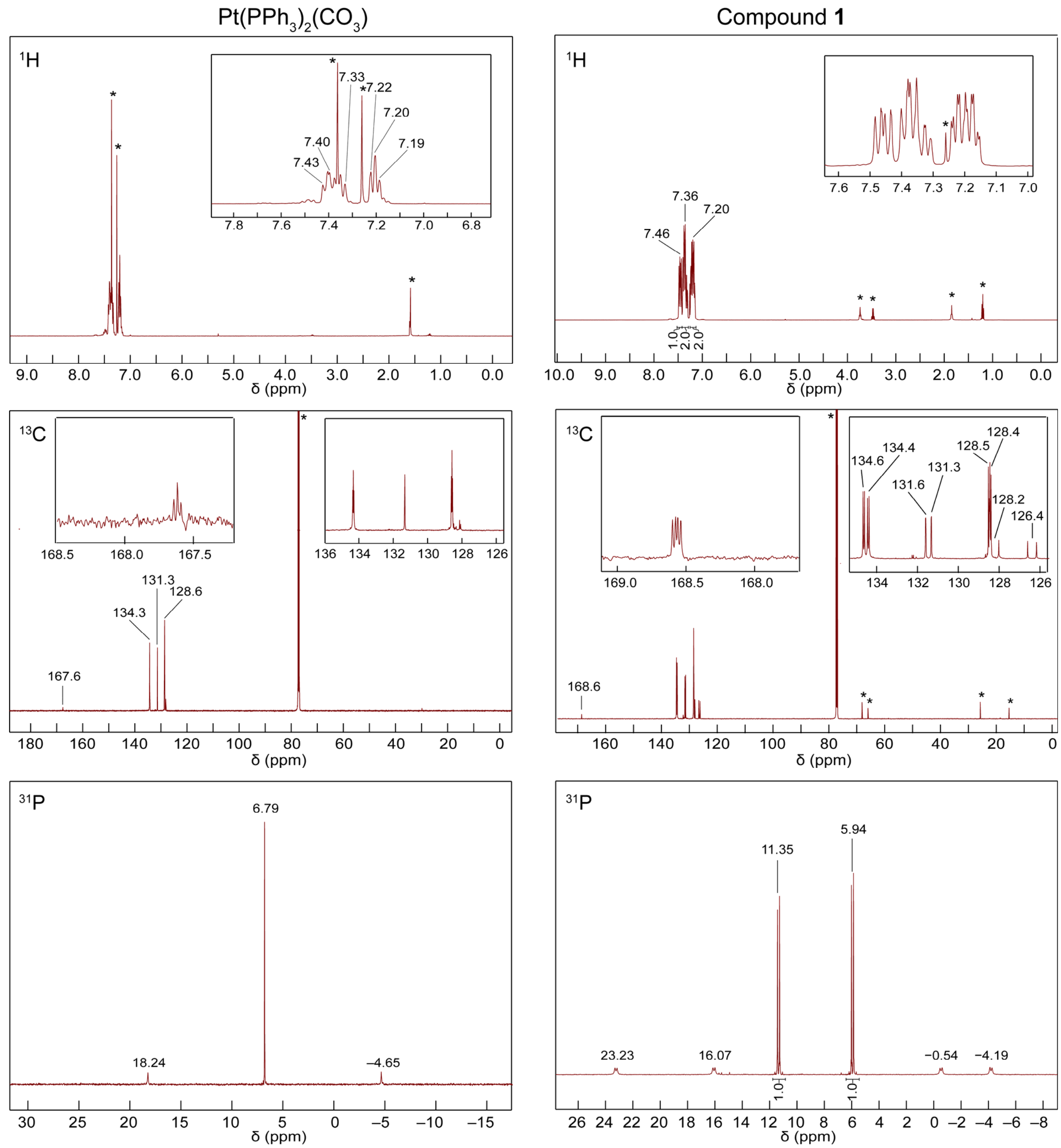


**Figure S8**. (Top) $^{1}H$ NMR (400 MHz, $CDCl_3$) spectrum of $Pt(PPh_3)_2(CO_3)$ (asterisks correspond to $C_6H_6$ (7.36 ppm), residual $CHCl_3$ (7.26 ppm), and $H_2O$ (1.56 ppm)) and **1** (asterisks correspond to residual $CHCl_3$ (7.26 ppm), THF (7.34, 1.85 ppm), and diethyl ether (3.47, 1.20 ppm)). (Middle) $^{13}C\{^{1}H\}$ NMR (151 MHz, $CDCl_3$) spectrum of $Pt(PPh_3)_2(CO_3)$ (asterisk corresponds to $CDCl_3$ (77.16 ppm) and **1** (asterisks correspond to $CDCl_3$ (77.16 ppm), residual THF (68.1, 25.8 ppm), and residual diethyl ether (66.0, 15.4 ppm)). Insets show peroxy/carbonate (left) and the aromatic (right) regions. (Bottom) $^{31}P\{^{1}H\}$ NMR (162 MHz, $CDCl_3$) spectrum of $Pt(PPh_3)_2(CO_3)$ and **1**.

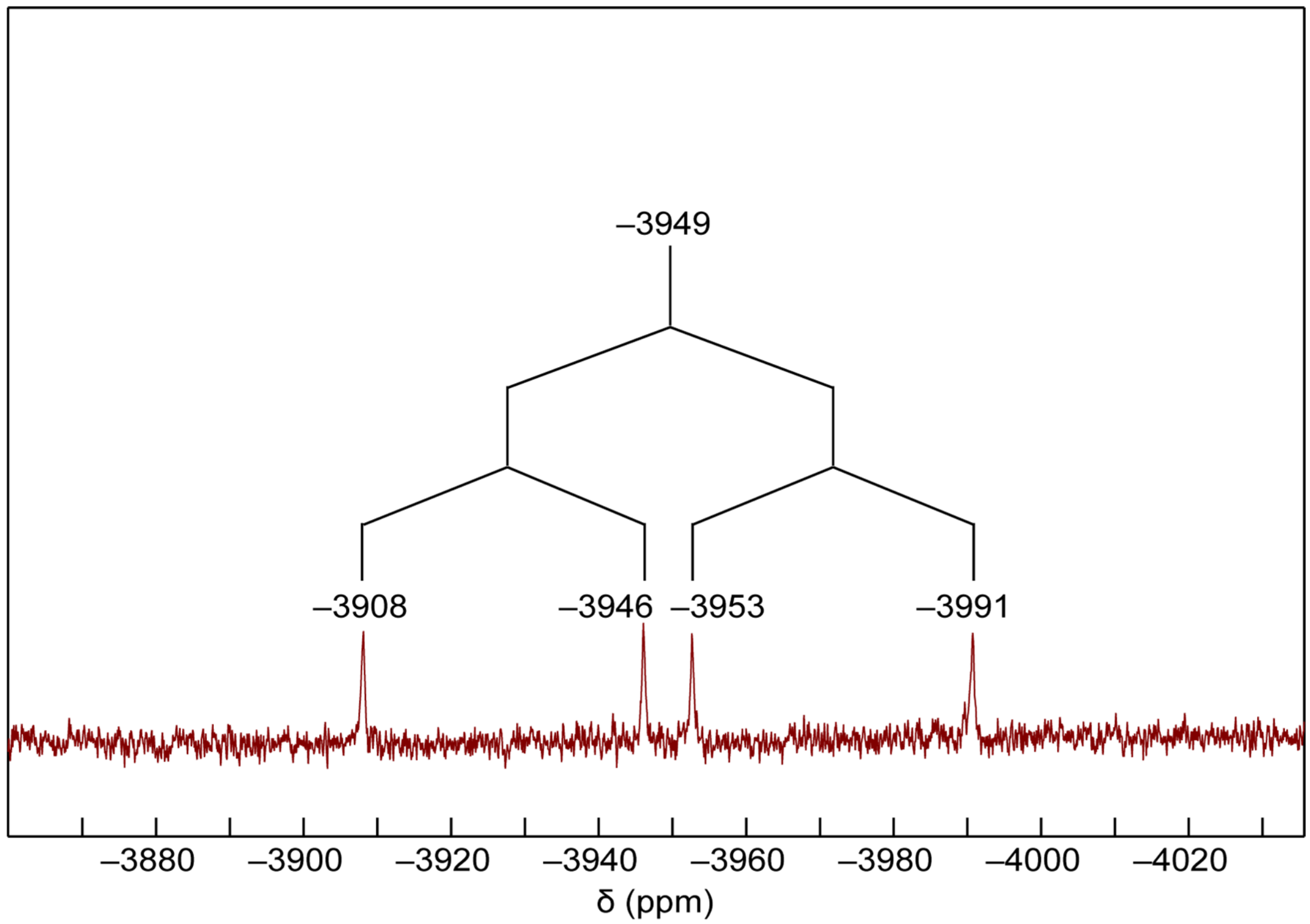


**Figure S9.** $^{195}Pt$ NMR (86 MHz, $CDCl_3$) spectrum of **1**.

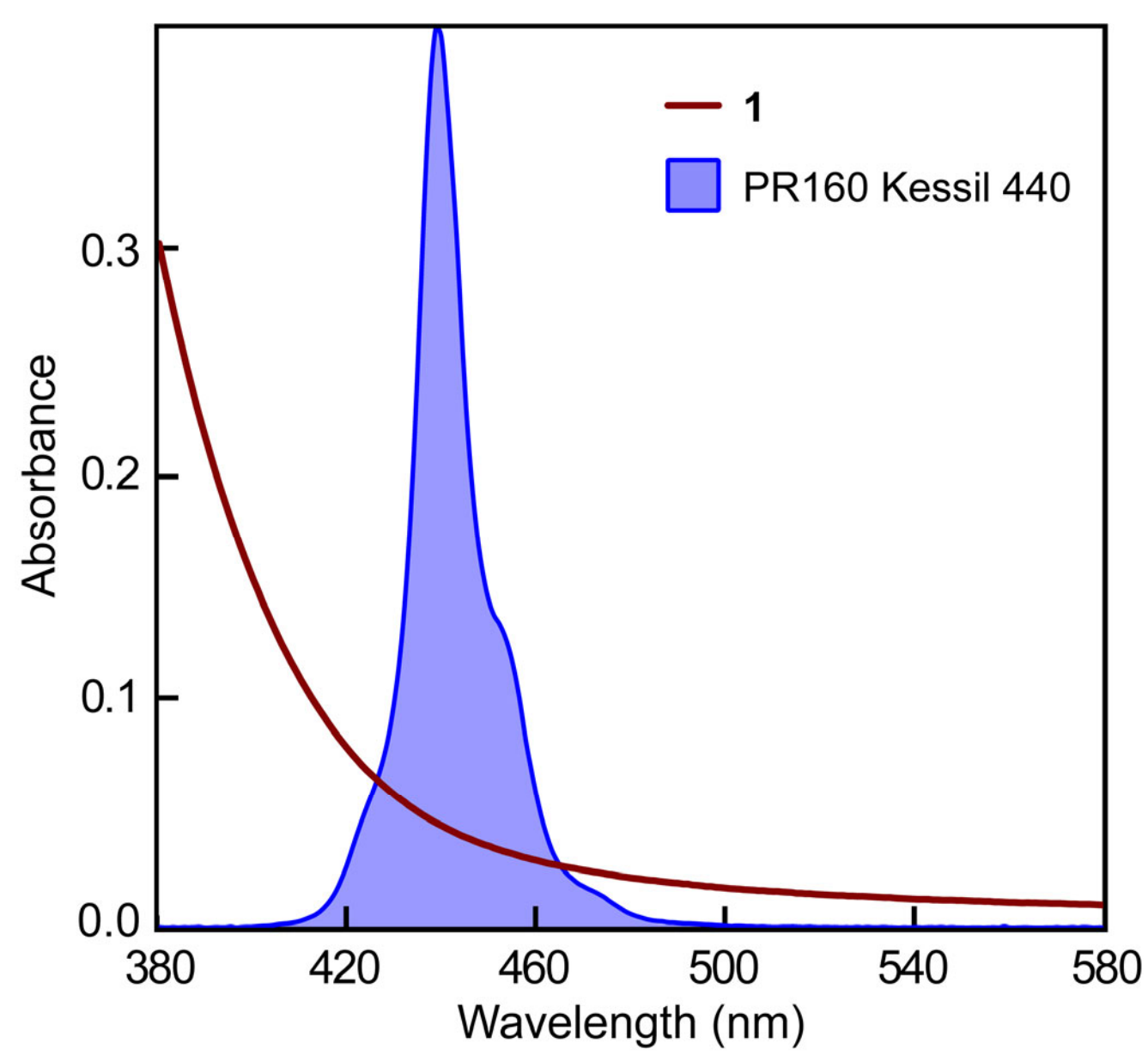


**Figure S10.** UV-vis spectrum of **1** (5 mM) in $CH_3CN$ (red trace). Normalized intensity profile (blue trace) of a 440 nm LED excitation source used for photochemical reactions.

## C.2. Reactivity

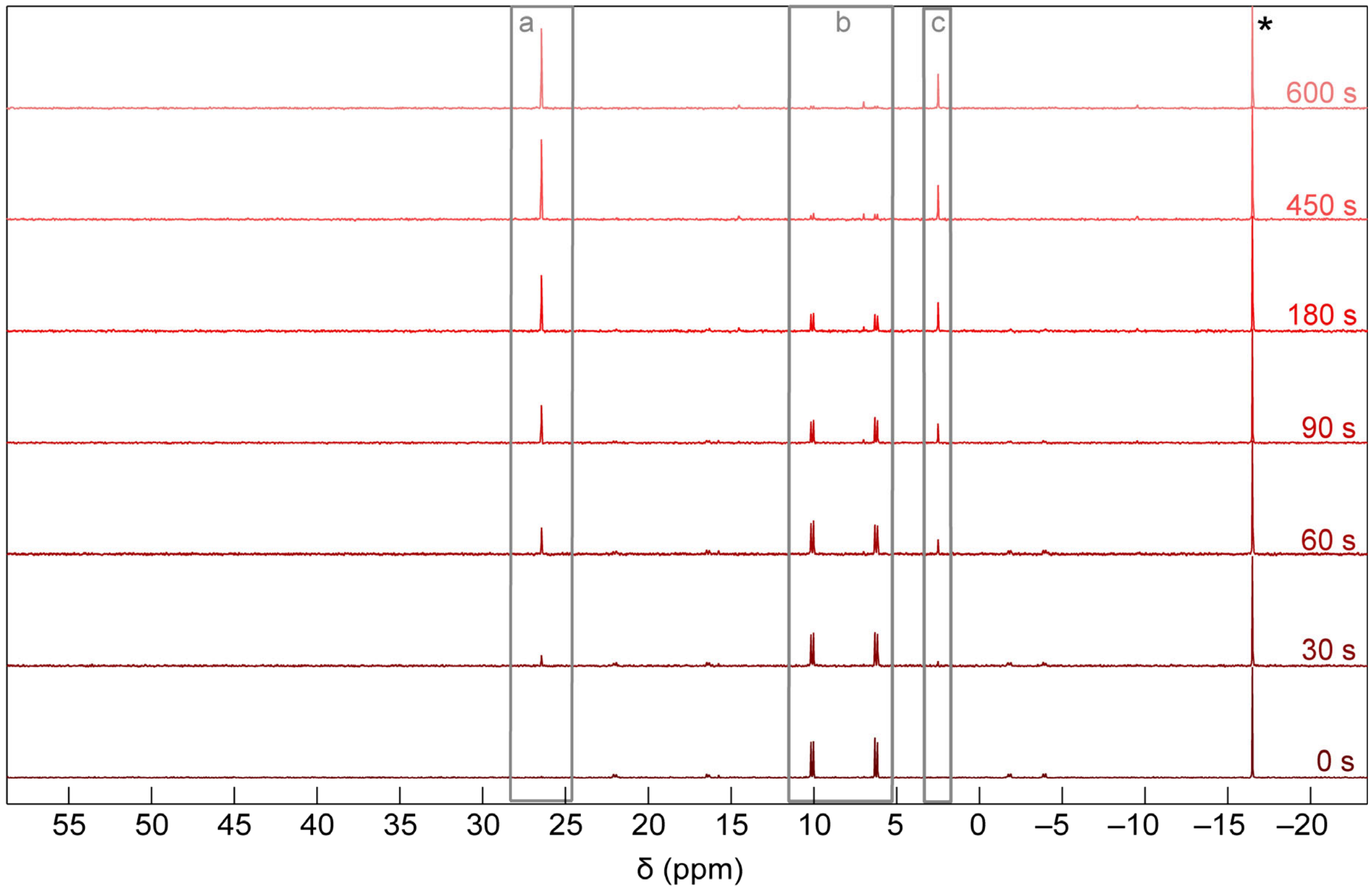


**Figure S11.** $^{31}P\{^1H\}$ NMR monitored photolysis ($\lambda_{exc}$ = 440 nm LED) of **1** (5 mM) in $CH_3CN$ from 0 to 600 s. Region "a" shows the growth of $OPPh_3$. Region "b" shows the decay of **1**. Region "c" shows the appearance of $Pt(PPh_3)(CH_3CN)(CO_3)$. No peaks at δ > 50 ppm, attributable to $Pt(PPh_3)(OPPh_3)(CO_3)$ are observed. Asterisk (*) corresponds to triphenyl phosphate internal standard.

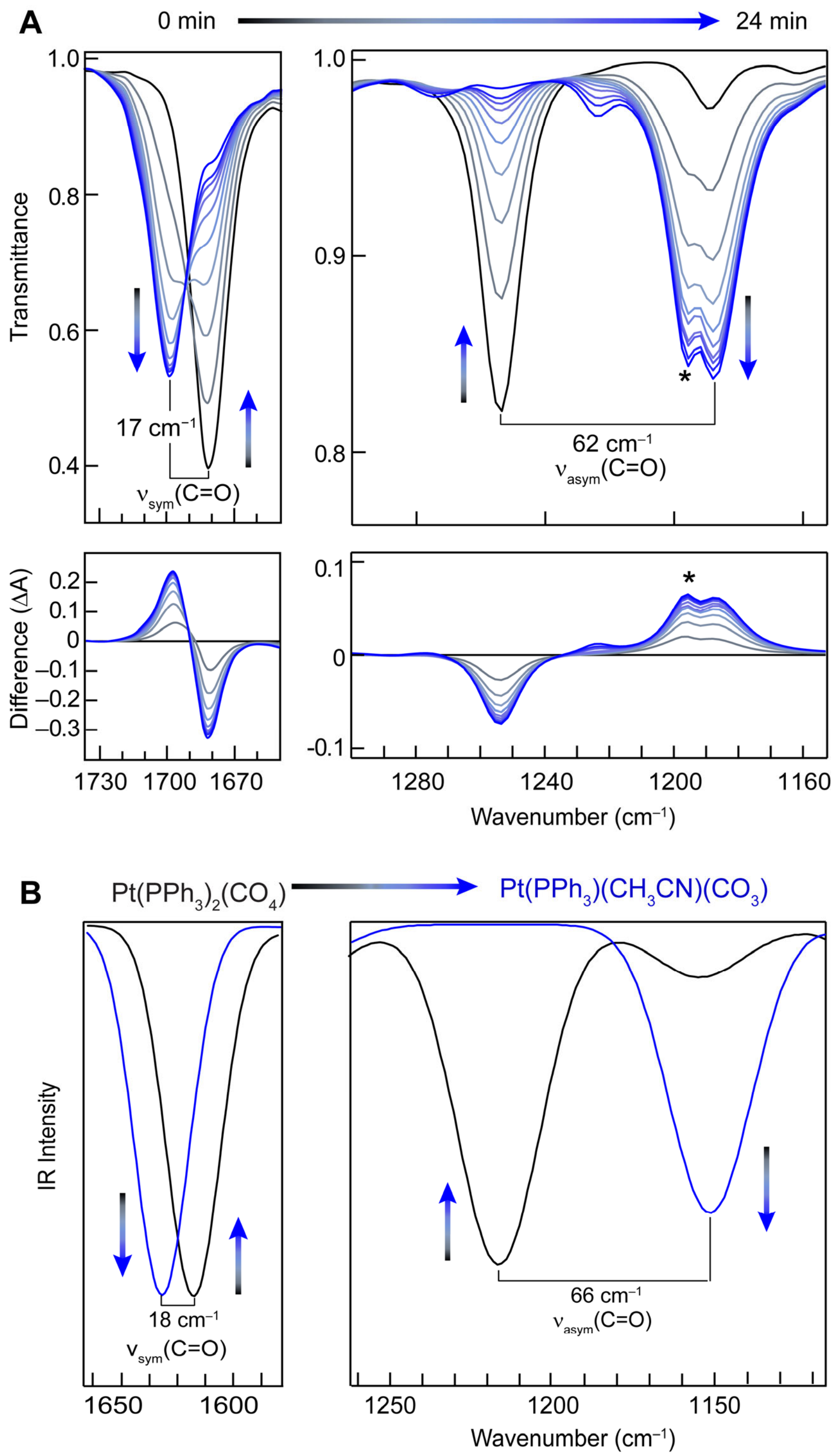


**Figure S12.** (**A**) FT-IR monitored photolysis ($\lambda_{exc}$ = 440 nm LED) of **1** (5 mM) in $CD_3CN$ (top) and difference spectra (bottom) from 0 to 24 min. The asterisk (*) corresponds to the IR stretch of $OPPh_3$ produced upon irradiation. (**B**) Calculated IR spectrum for DFT-optimized geometry of **1** (black, **Table S7**) and $Pt(PPh_3)(CH_3CN)(CO_3)$ photoproduct (blue, **Table S8**) with $CH_3CN$ implicit solvation. Spectra are normalized to the most intense peak.

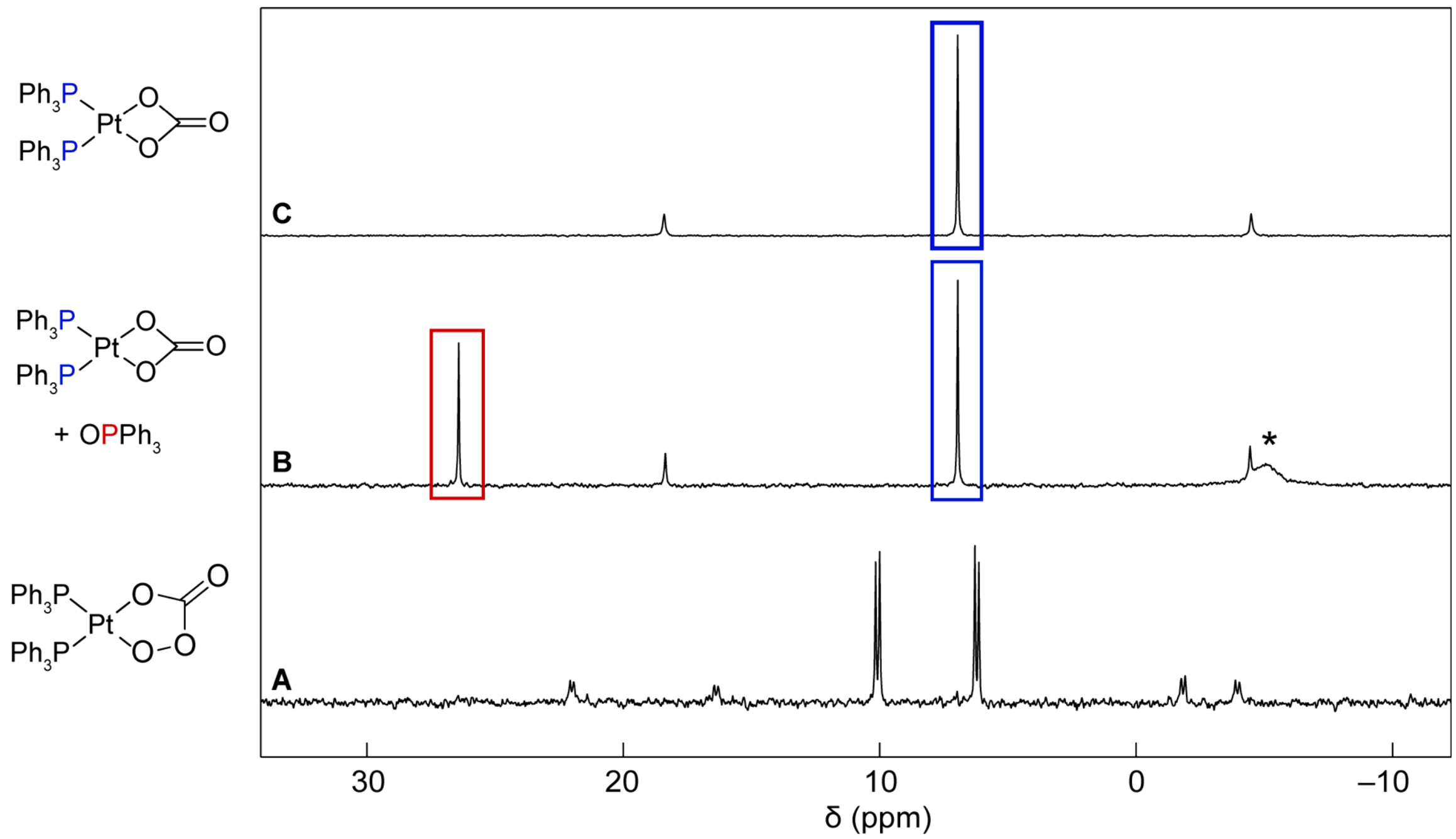


**Figure S13.** ${}^{31}P\{{}^{1}H\}$ NMR spectra in $CH_3CN$. Corresponding structures are shown to the left of each spectrum with unique ${}^{31}P$ nuclei denoted in red (P=O) and blue (P): (**A**) a 5 mM solution of **1**, (**B**) a 5 mM solution of **1** with 3 equiv $PPh_3$ and 15 min of irradiation ($\lambda_{exc}$ = 440 nm LED), and (**C**) a 5 mM solution of $Pt(PPh_3)_2(CO_3)$ for comparison with spectrum "B". Photochemical generation of 1 equiv of $OPPh_3$ and $Pt(PPh_3)_2(CO_3)$ is evident in spectrum "B". Asterisk (*) corresponds to unreacted $PPh_3$.

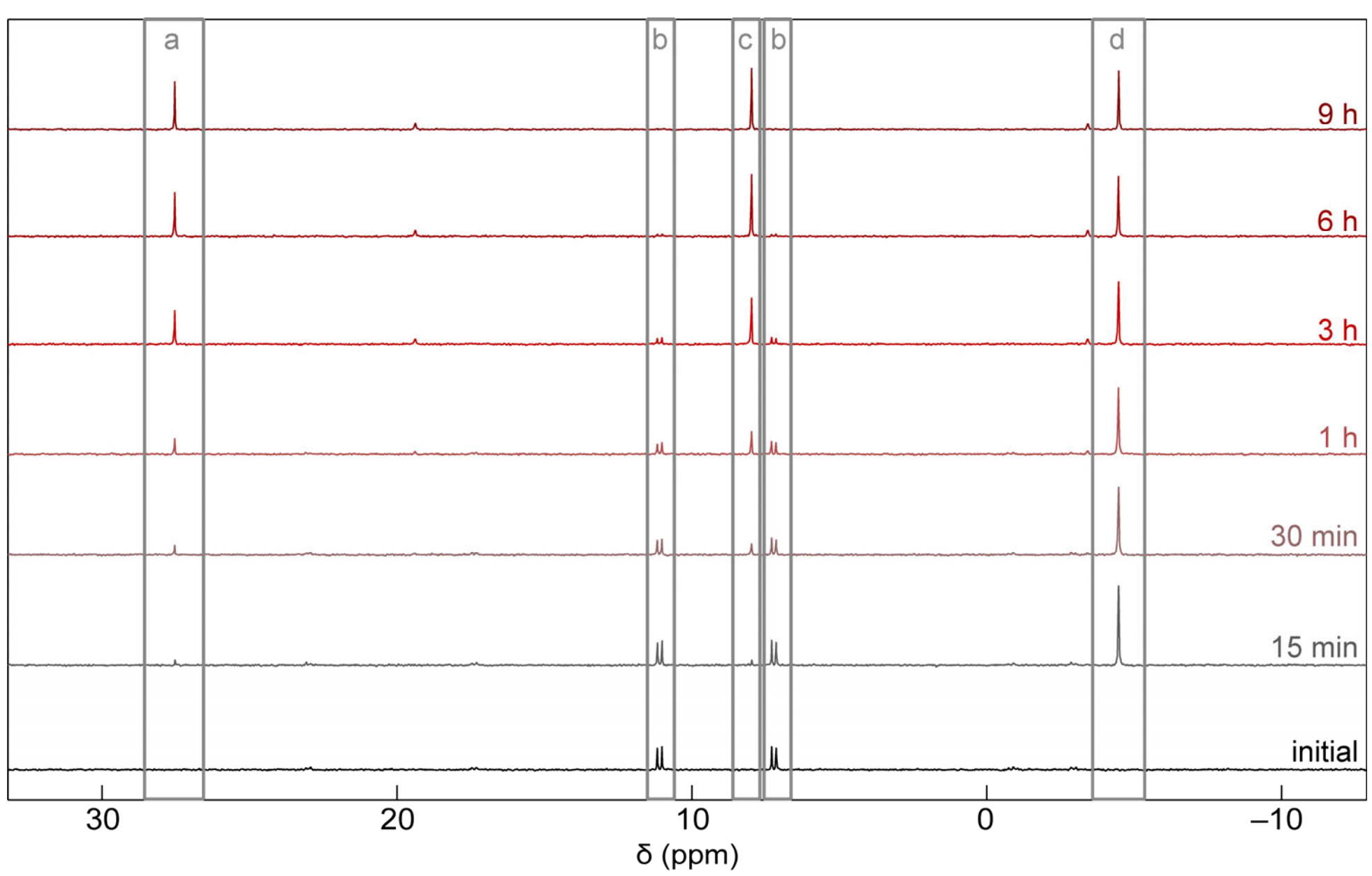


**Figure S14.** ${}^{31}P\{{}^{1}H\}$ NMR spectra of **1** (5 mM) in $CH_3CN$ before (bottom, black) and after (15 min, 30 min, 1 h, 3 h, 6 h, and 9 h) addition of 3 equiv $PPh_3$. The sample was kept in darkness, at room temperature. Complete reaction of **1** is observed within 9 hours. Region "a" shows the growth of $OPPh_3$. Region "b" shows the decay of **1**. Region "c" shows the growth of $Pt(PPh_3)_2(CO_3)$. Region "d" shows excess $PPh_3$.

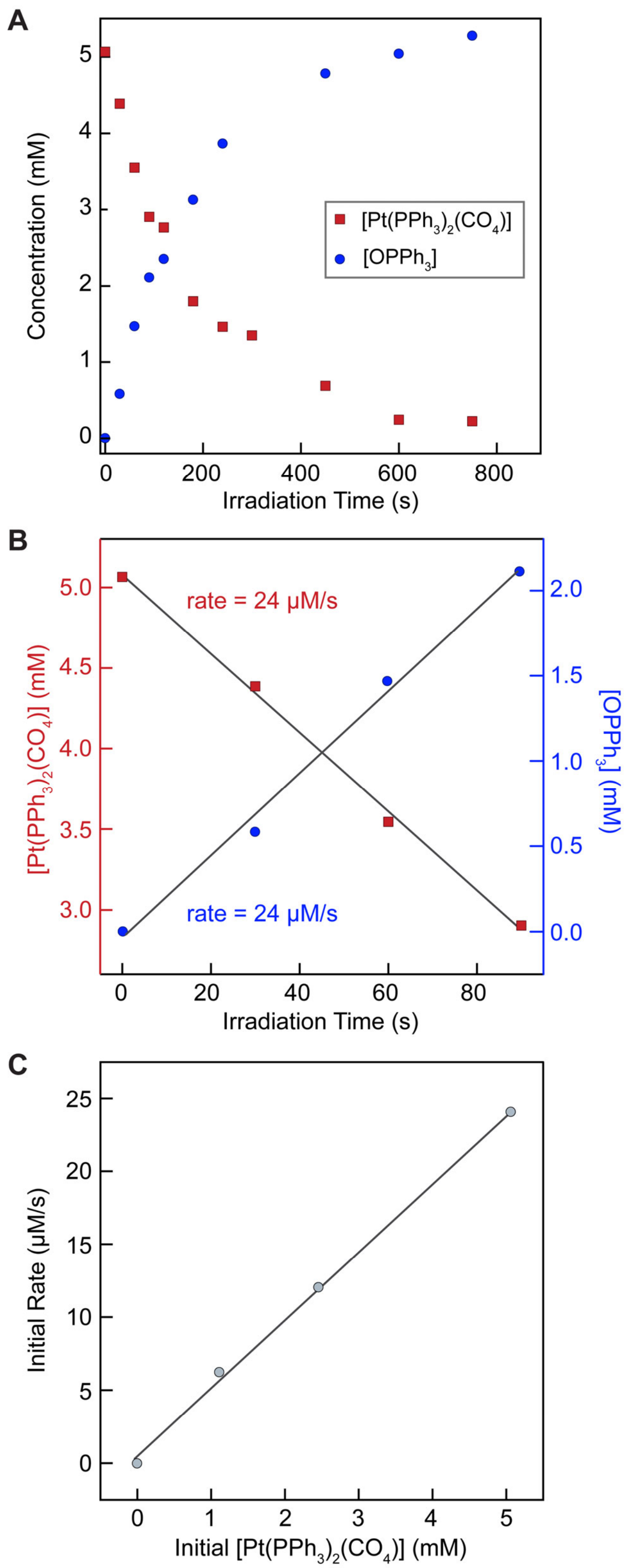


**Figure S15**. Photolysis ($\lambda_{exc}$ = 440 nm LED) of **1** in $CH_3CN$. (**A**) Plot of concentration vs time for the disappearance of **1** and appearance of $OPPh_3$. (**B**) Initial rates of for the disappearance of **1** and appearance of $OPPh_3$. (**C**) Plot of initial rates of **1** consumption as a function of initial [**1**].

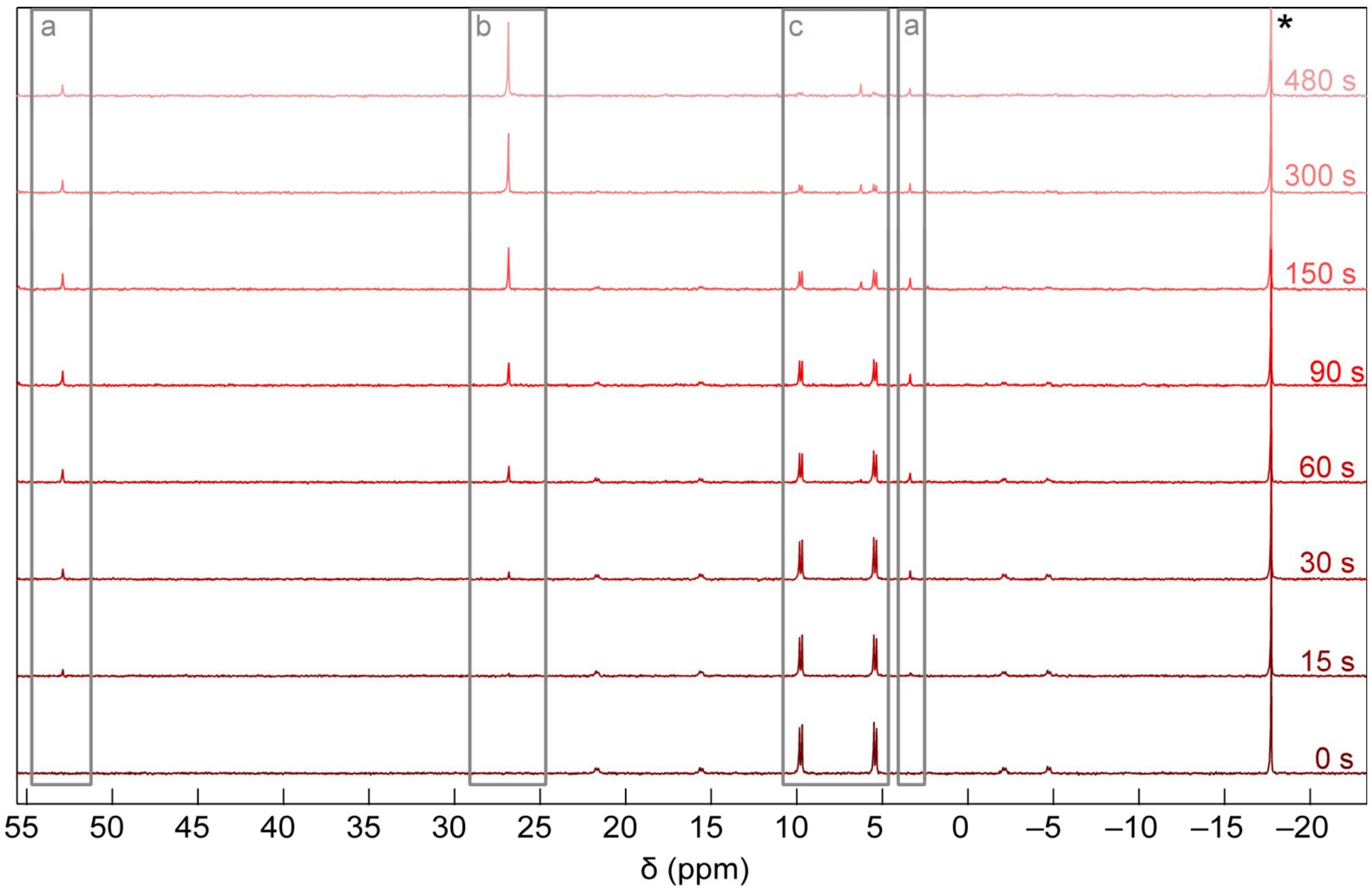


**Figure S16.** $^{31}P\{^{1}H\}$ NMR monitored photolysis ($\lambda_{exc}$ = 440 nm LED) of **1** (15 mM) in $CH_2Cl_2$ from 0 to 480 s. Region "a" shows the growth of $Pt(PPh_3)(OPPh_3)(CO_3)$. Region "b" shows the growth of $OPPh_3$. Region "c" shows the decay of **1**. Asterisk (*) corresponds to triphenyl phosphate internal standard.

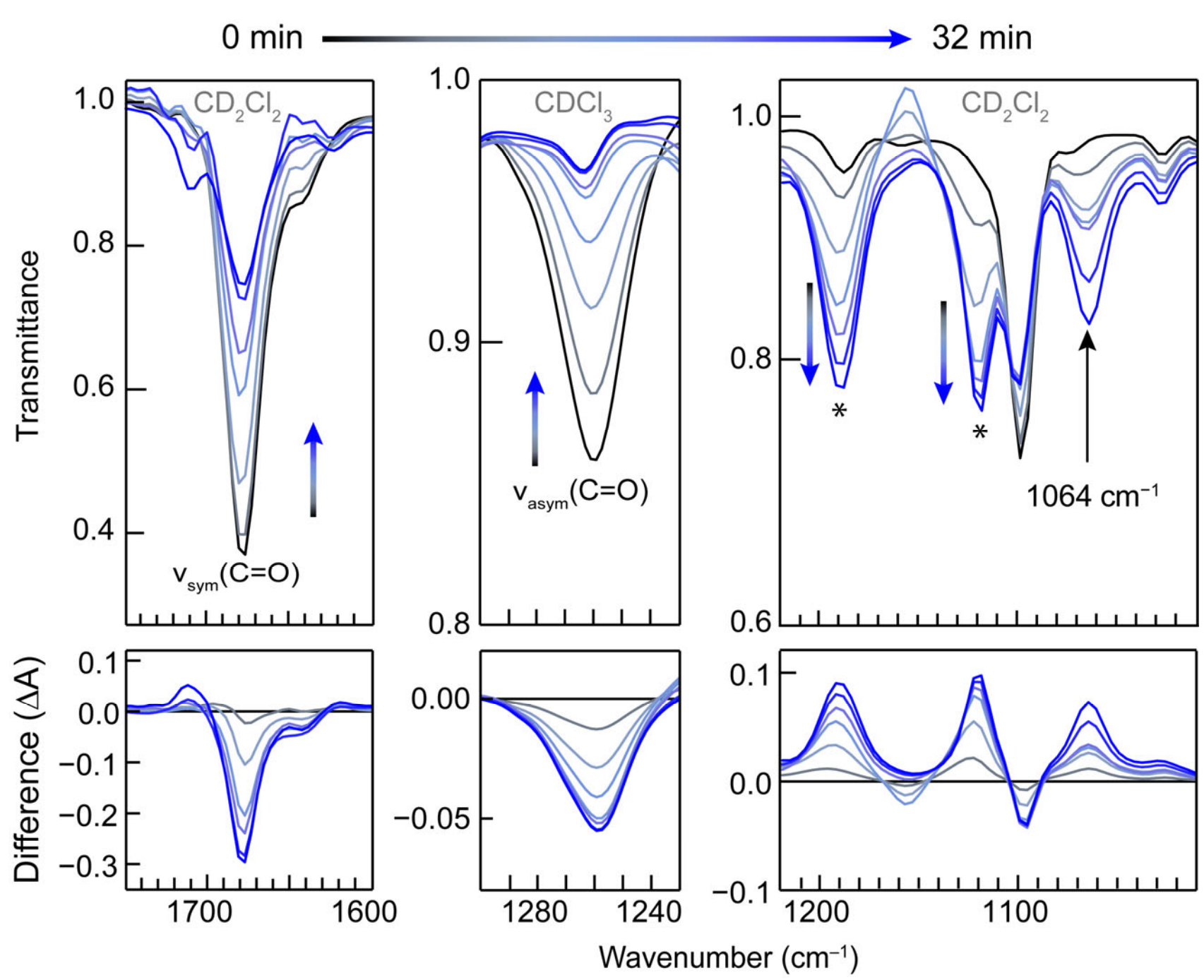


**Figure S17.** FT-IR monitored photolysis ($\lambda_{exc}$ = 440 nm LED) of **1** (5 mM) from 0 to 32 min. The solvent used is denoted at top of each spectrum in grey; the use of $CD_2Cl_2$ and $CDCl_3$ is necessary to view all relevant stretches for both decay of **1** and growth of $OPPh_3$. $OPPh_3$ peaks are marked with an asterisk (*). The peak at 1064 $cm^{-1}$, assigned to a P=O—Pt stretch of $Pt(PPh_3)(OPPh_3)(CO_3)$, is highlighted in the rightmost pane.

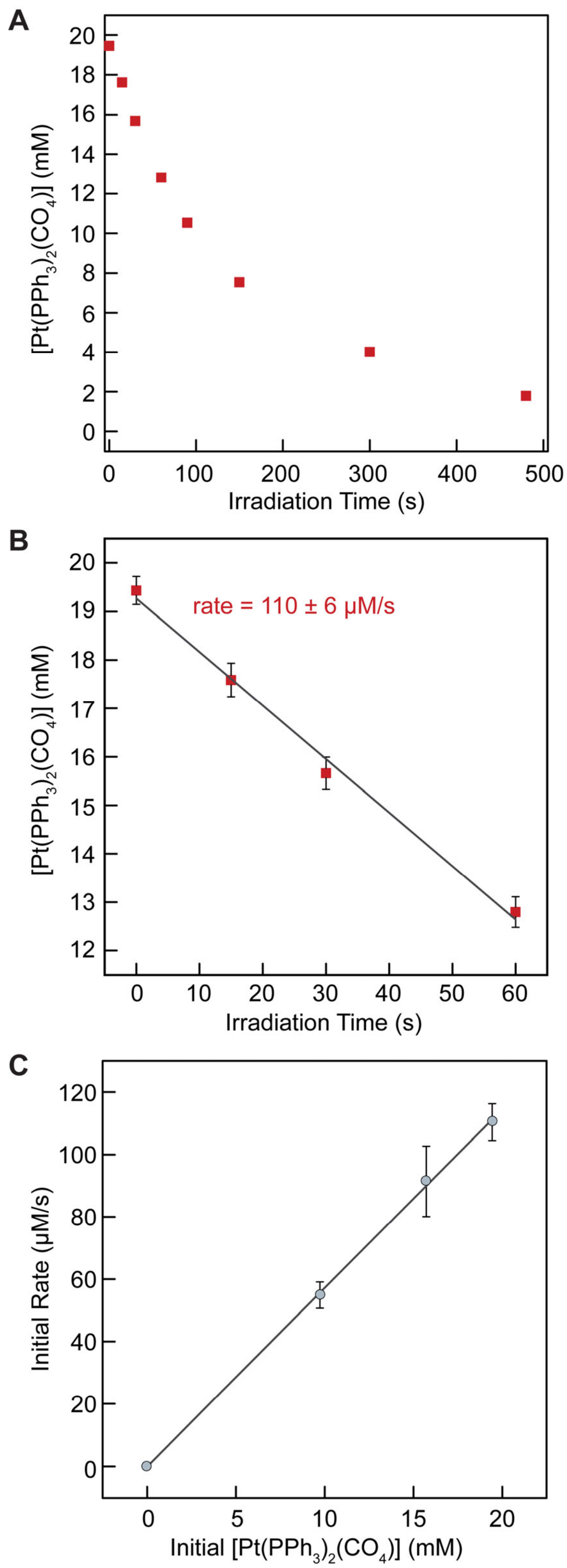


**Figure S18**. Photolysis ($\lambda_{exc}$ = 440 nm LED) of **1** in $CH_2Cl_2$. (**A**) Plot of concentration vs time for the disappearance of **1** (19 mM). (**B**) Initial rate for the disappearance of **1**. (**C**) Plot of initial rates of **1** consumption as a function of initial [**1**]. For (**B**) and (**C**), each data point represents the average of three measurements on three different samples.

## D. Compound 2

### D.1. Spectroscopic Characterization

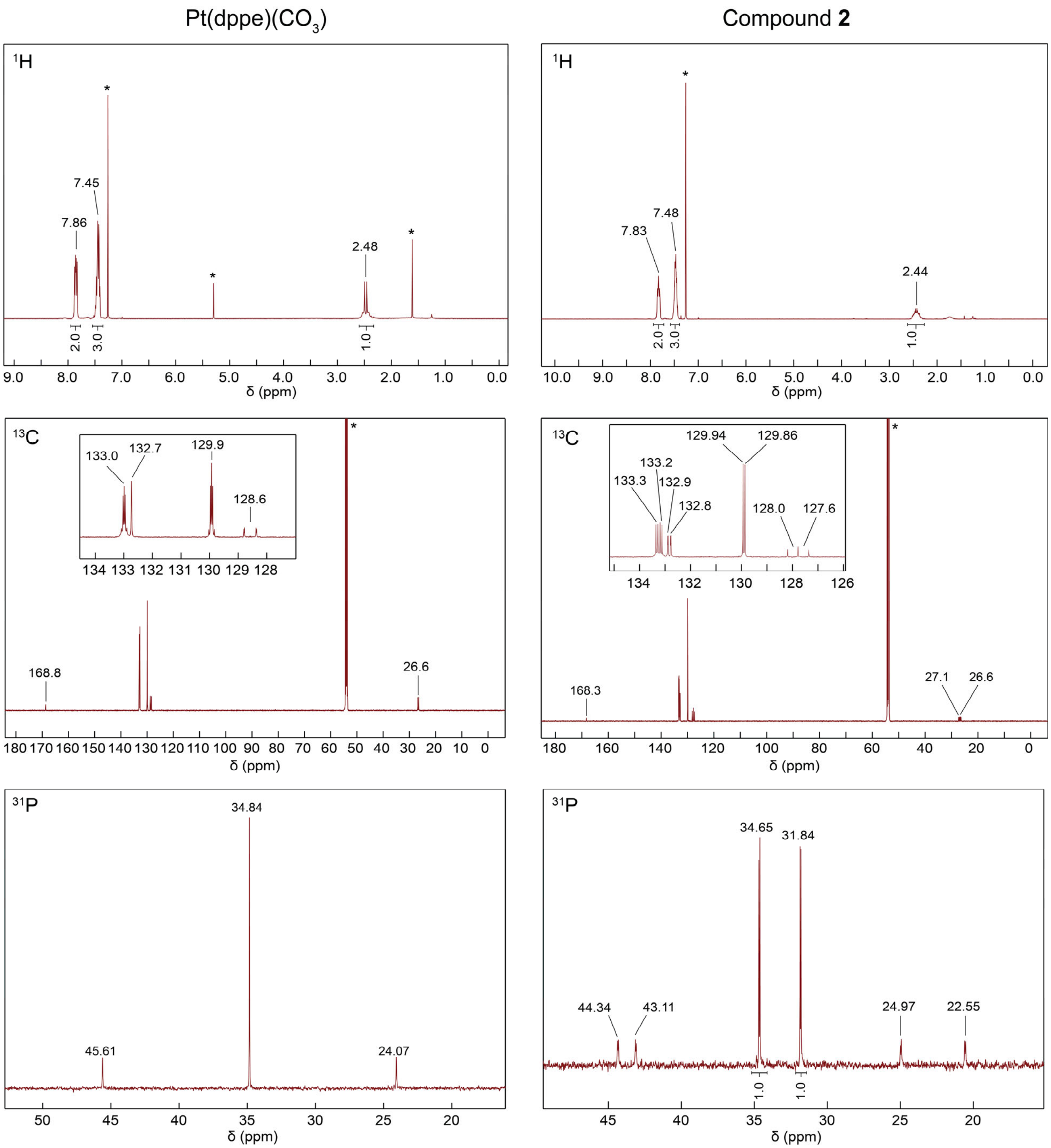


**Figure S19.** (Top) $^{1}H$ NMR (400 MHz, $CDCl_3$) spectrum of Pt(dppe)($CO_3$) (asterisks correspond to residual $CHCl_3$ (7.26 ppm), $CH_2Cl_2$ (5.30 ppm), and $H_2O$ (1.61 ppm)) and **2** (asterisk corresponds to $CDCl_3$ (7.26 ppm)). (Middle) $^{13}C\{^{1}H\}$ NMR (151 MHz, $CDCl_3$) spectrum of Pt(dppe)($CO_3$) (asterisk corresponds to $CD_2Cl_2$ (54.00 ppm) and **2** (asterisk corresponds to $CD_2Cl_2$ (54.00 ppm)) Insets show the aromatic (region). (Bottom) $^{31}P\{^{1}H\}$ NMR (162 MHz, $CD_3CN$) spectrum of Pt(dppe)($CO_3$) and **2**.

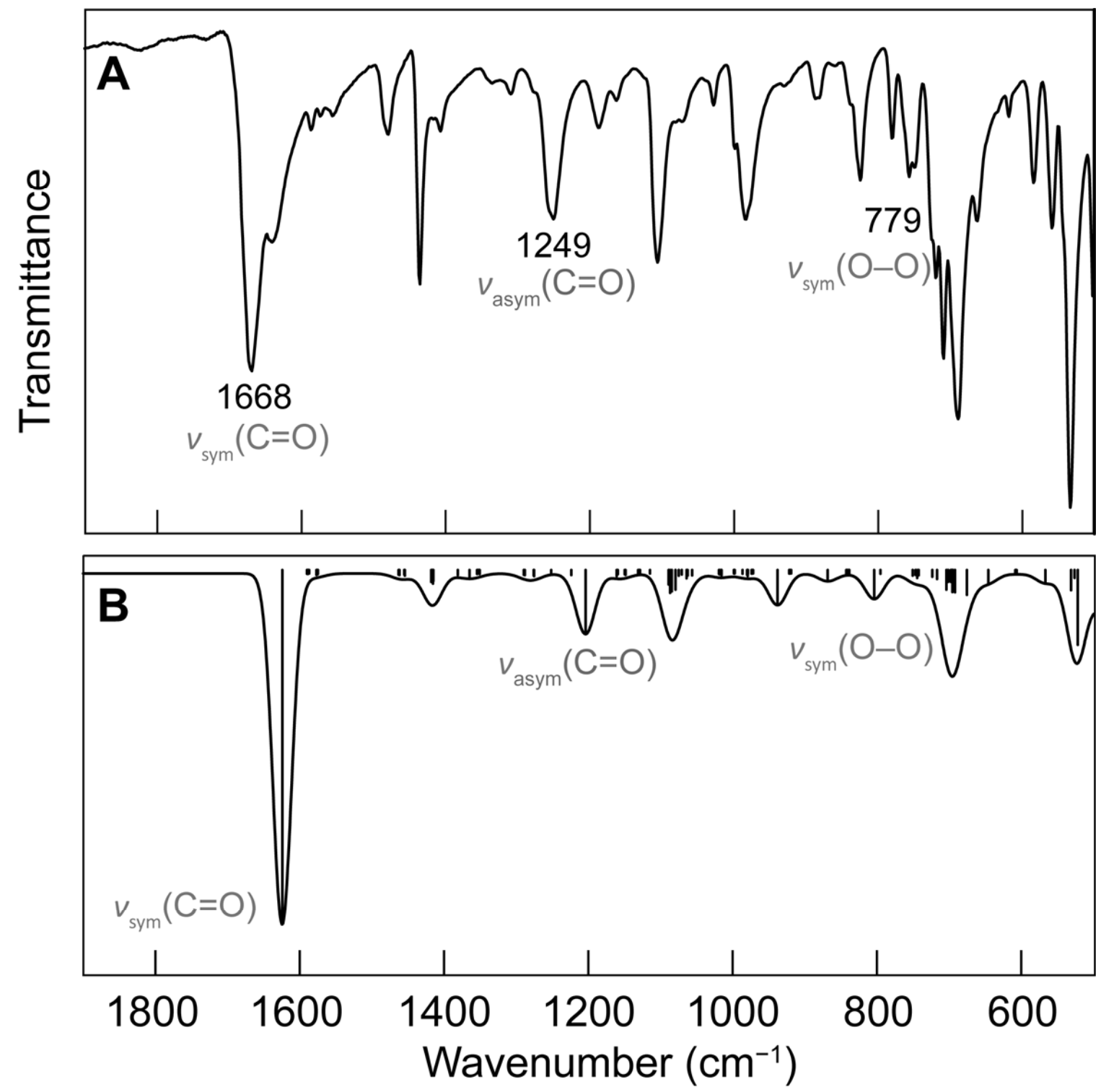


**Figure S20.** (**A**) FT-IR spectra (solid-state) of **2**. (**B**) Calculated IR frequencies and resultant spectrum for DFT-optimized geometry of **2** (**Table S9**) with $CH_2Cl_2$ implicit solvation. Selected IR stretches are labeled (gray).

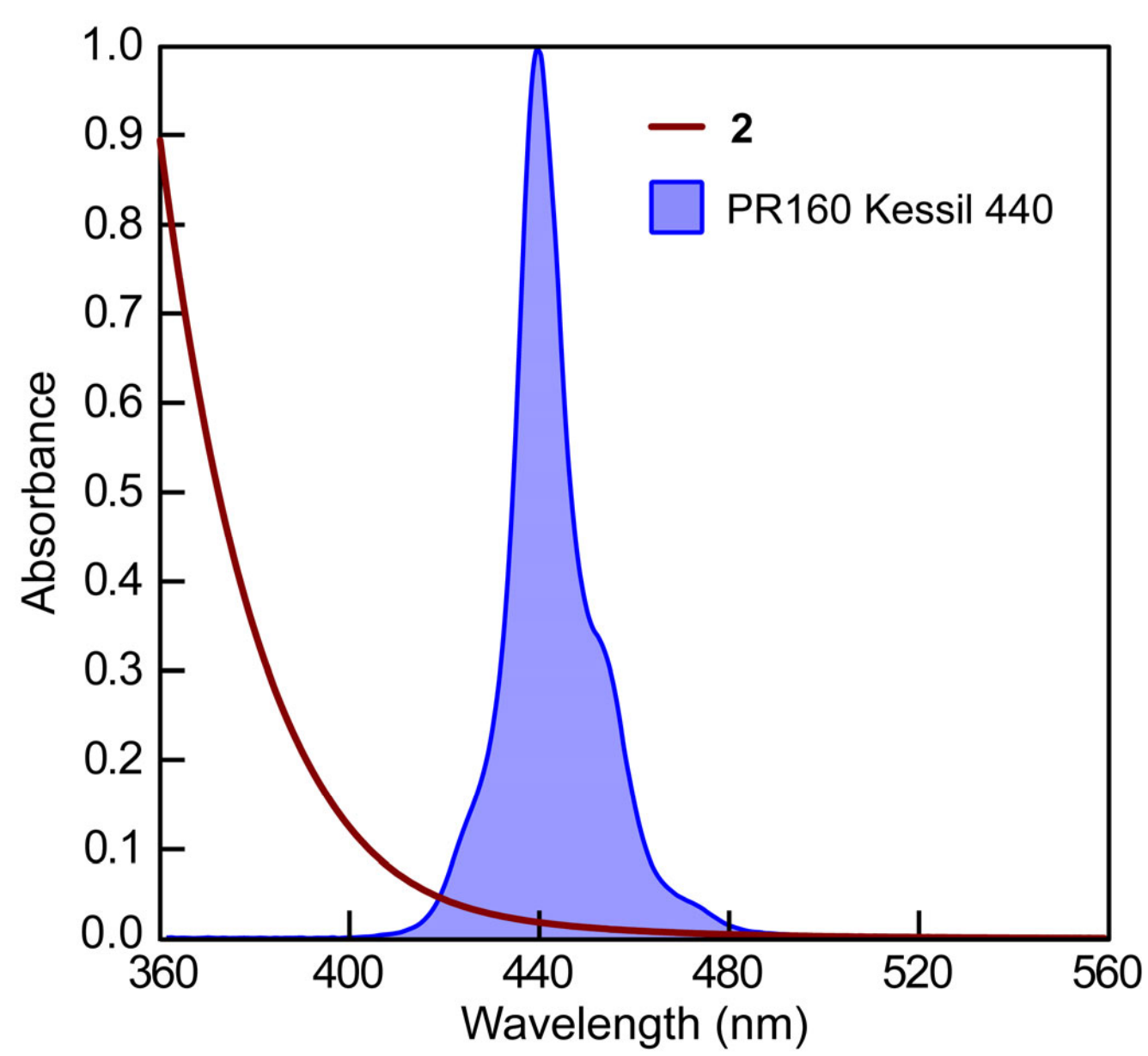


**Figure S21.** UV-vis spectrum of **2** (15 mM) in $CH_2Cl_2$ (red trace). Normalized intensity profile (blue trace) of a 440 nm LED excitation source used for photochemical reactions.

## D.2. Reactivity

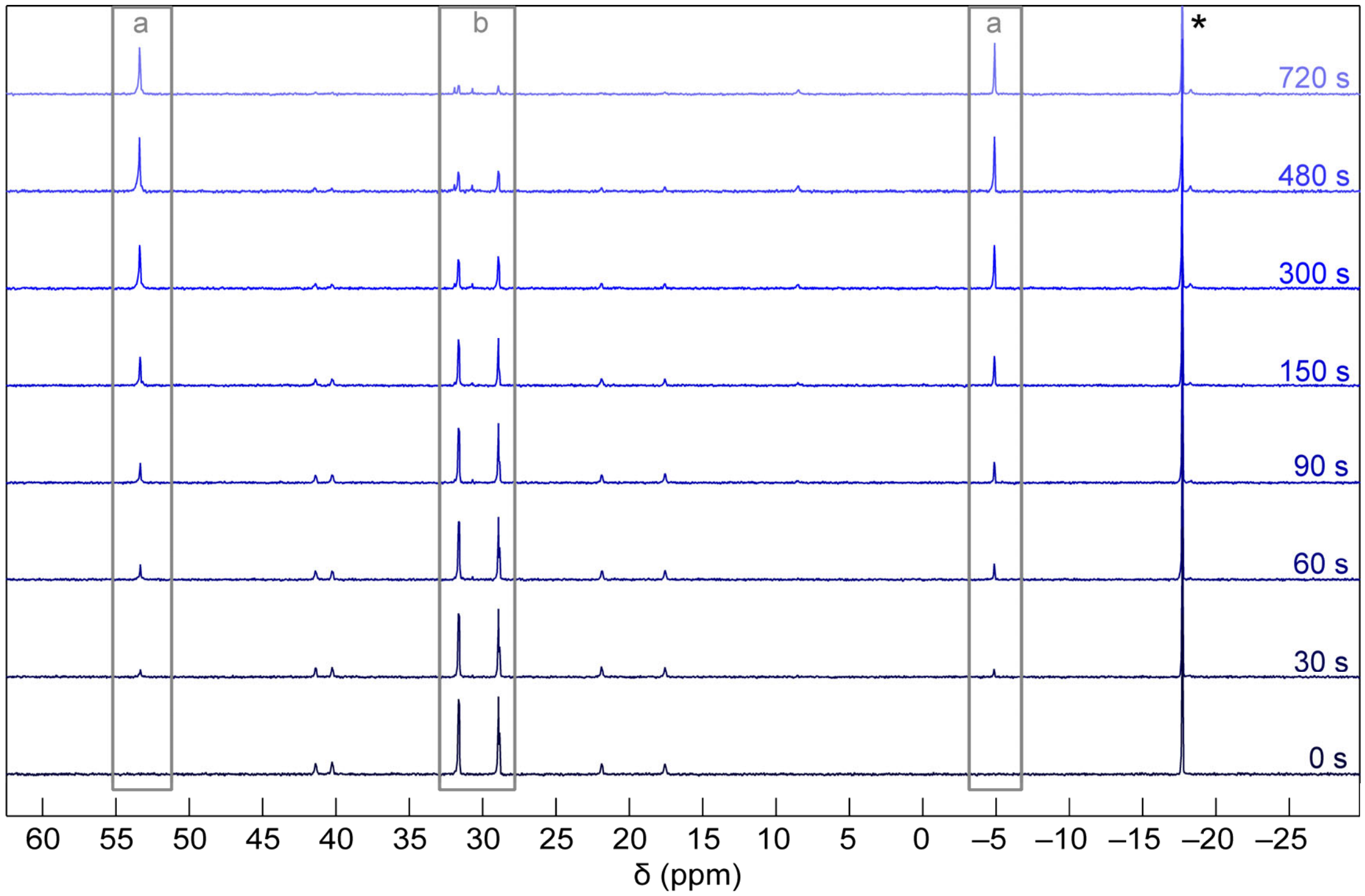


Figure S22. $^{31}P\{^{1}H\}$ NMR monitored photolysis ($\lambda_{exc}$ = 440 nm LED) of 2 (20 mM) in $CH_2Cl_2$ from 0 to 720 s. Regions "a" show the growth of Pt(dppeO)($CO_3$). Region "b" shows the decay of 2. Asterisk (*) corresponds to triphenyl phosphate internal standard.

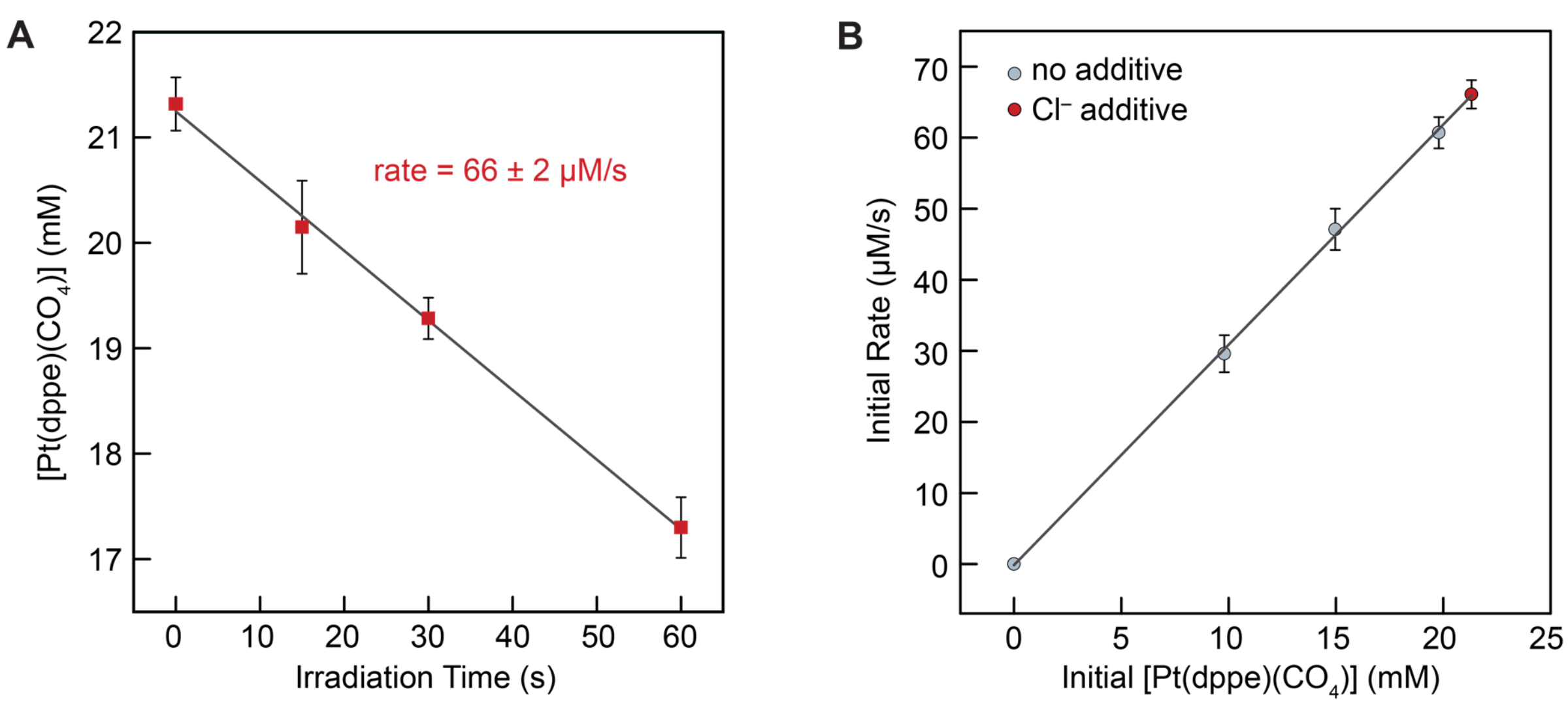


Figure S23. Photolysis ($\lambda_{exc}$ = 440 nm LED) of 2 in $CH_2Cl_2$. (A) Initial rate for the disappearance of 2 when photolyzed in the presence of a large excess of PPN[Cl] (6 equiv). (B) Plot of initial rate of 2 consumption as a function of initial [2], where the red data point is the initial rate measured in panel A plotted with measured initial rates in the absence of $Cl^-$. For (A) and (B), each data point represents the average of three measurements on three different samples.

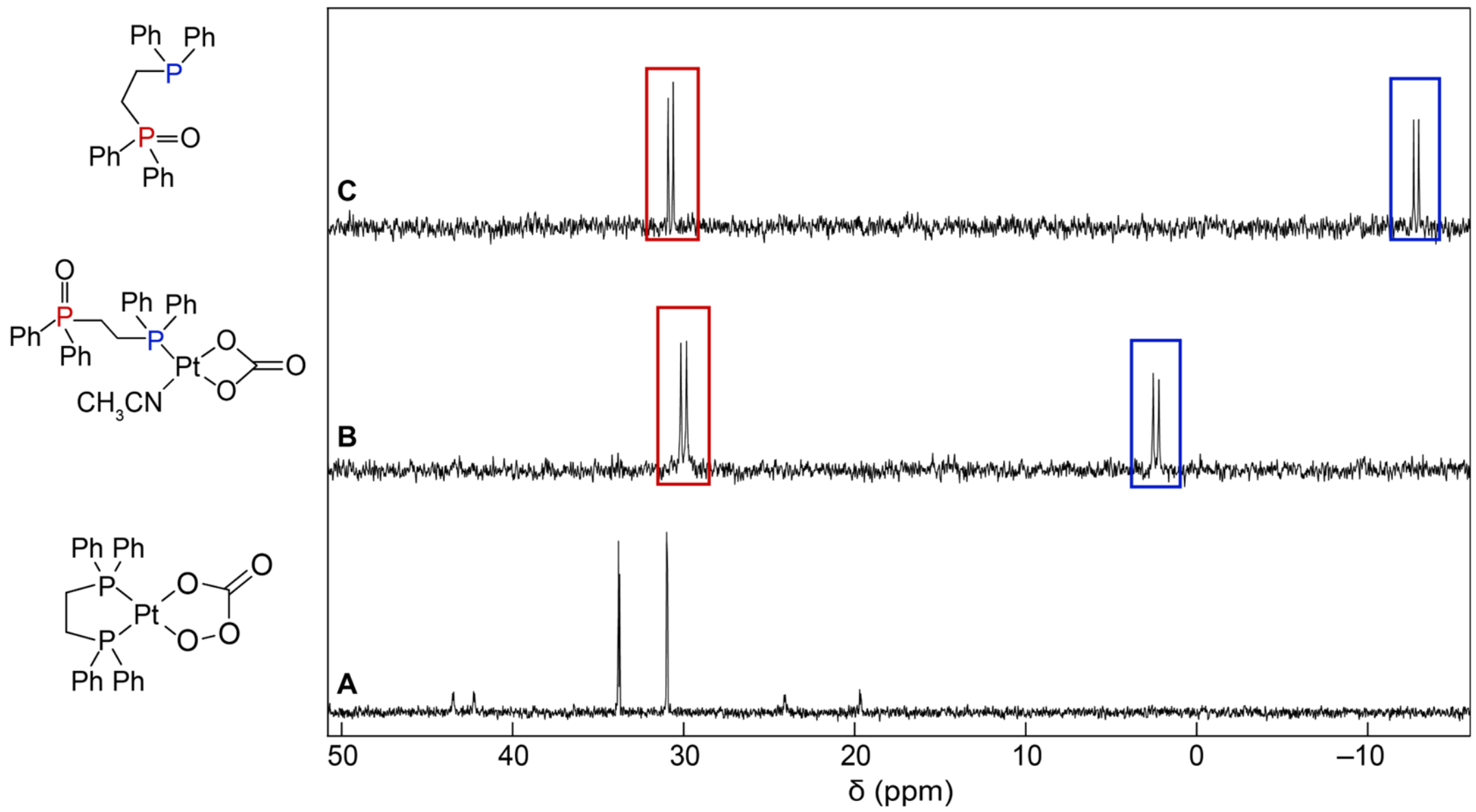


**Figure S24.** ${}^{31}P\{{}^{1}H\}$ NMR spectra in $CH_3CN$. Corresponding structures are shown to the left of each spectrum with unique ${}^{31}P$ nuclei denoted in red (P=O) and blue (P). (**A**) **2** (5 mM). (**B**) Pt(dppeO)($CH_3CN$)($CO_3$) formed via complete photolysis of **2**. (**C**) dppeO for comparison with Pt(dppeO)($CH_3CN$)($CO_3$).

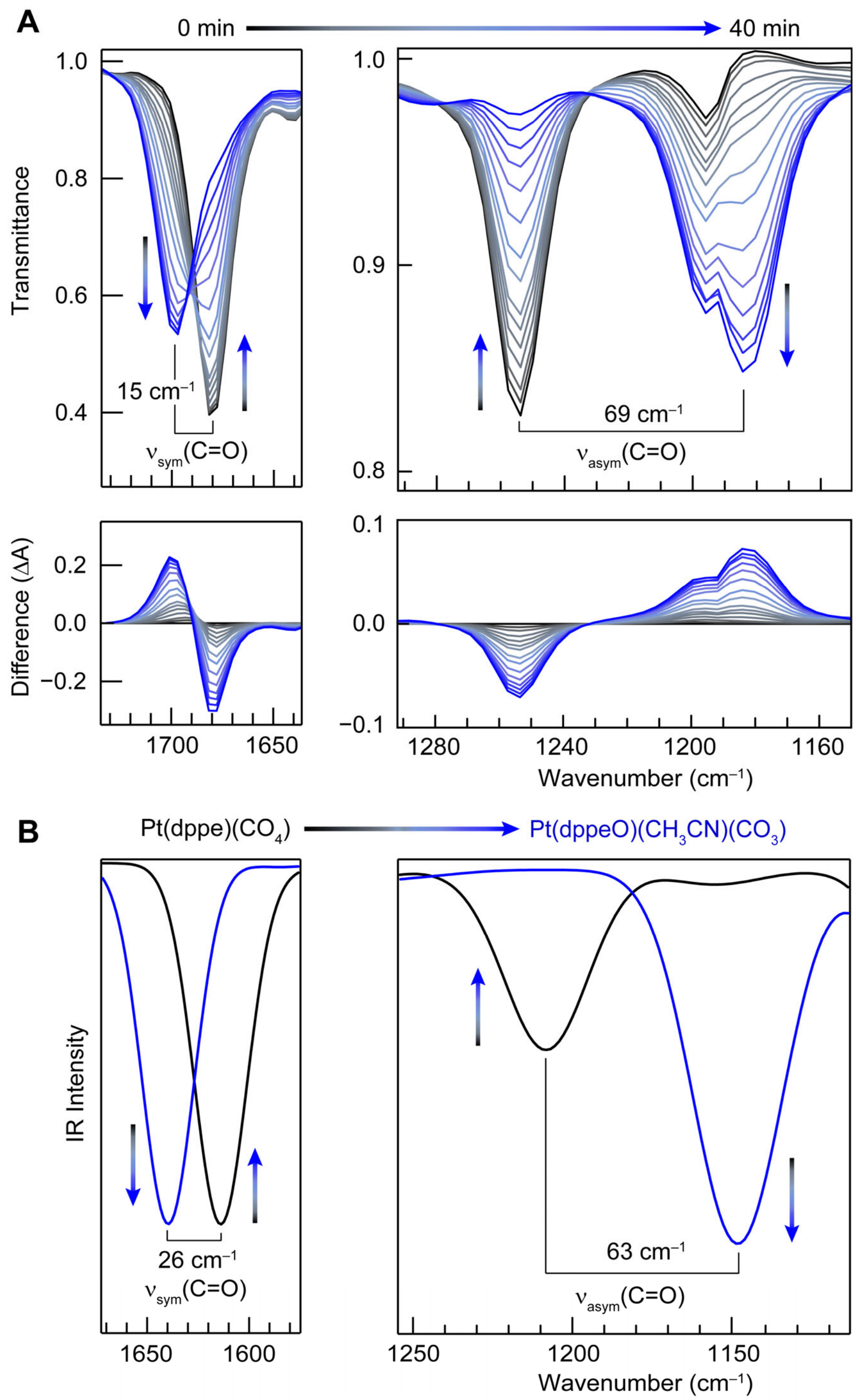


**Figure S25.** (**A**) FT-IR monitored photolysis ($\lambda_{exc}$ = 440 nm LED) of **2** (5 mM) in $CD_3CN$ (top) and difference spectra (bottom) from 0 to 40 min. (**B**) Calculated IR spectrum for DFT-optimized geometry of **2** (black, **Table S10**) and Pt(dppeO)($CH_3CN$)($CO_3$) photoproduct (blue, **Table S11**) with $CH_3CN$ implicit solvation. Spectra are normalized to the most intense peak.

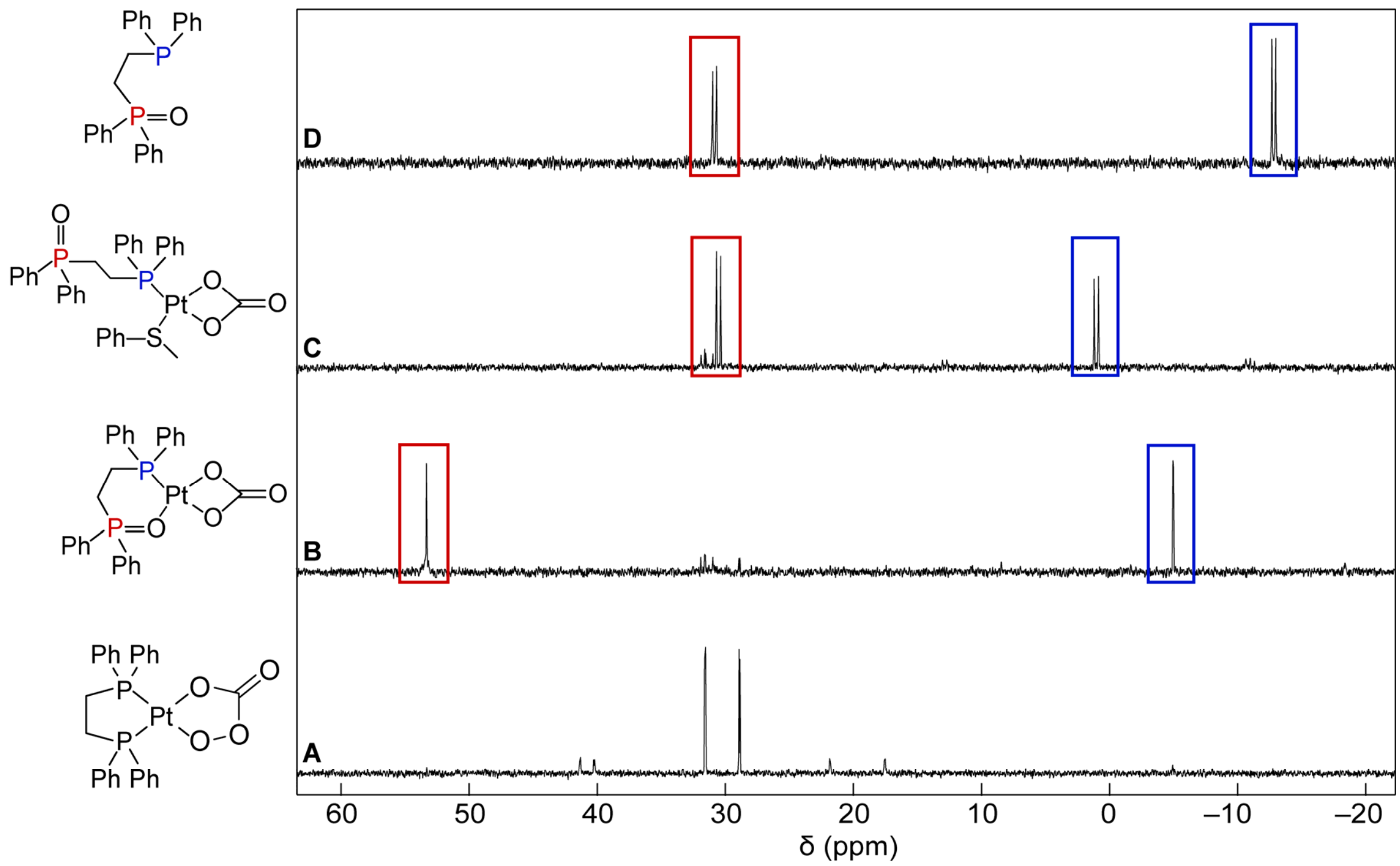


**Figure S26.** $^{31}P\{^{1}H\}$ NMR spectra in $CH_2Cl_2$. Corresponding structures are shown to the left of each spectrum with unique $^{31}P$ nuclei denoted in red (P=O) and blue (P). (**A**) **2** (10 mM). (**B**) Pt(dppeO)($CO_3$) formed via photolysis of **2**. (**C**) Pt(dppeO)(PhSMe)($CO_3$) formed by treatment of Pt(dppeO)($CO_3$) with 5 equiv of thioanisole. (**D**) dppeO for comparison with Pt(dppeO)(PhSMe)($CO_3$).

## E. Density Functional Theory Calculations

### E.1. Compound 1 and Related Calculations

**Table S6.** Coordinates for the DFT-optimized geometry of **1** with $CH_2Cl_2$ implicit solvation.

| Atom | Coordinates (Å) | | | Atom | Coordinates (Å) | | |
|---|---|---|---|---|---|---|---|
| Number | x | y | z | Number | x | y | z |
| Pt0 | 6.393674 | 5.373207 | 18.79566 | C37 | 3.930656 | 4.234179 | 23.00449 |
| P1 | 6.874199 | 3.643082 | 20.16424 | H38 | 3.060937 | 4.897082 | 23.13007 |
| P2 | 4.432732 | 4.628727 | 17.88871 | C39 | 4.701203 | 4.312780 | 21.83102 |
| O3 | 8.098823 | 6.310150 | 19.37572 | H40 | 4.442684 | 5.043973 | 21.05046 |
| C4 | 7.232726 | 7.926120 | 17.77836 | C41 | 2.99232 | 5.624081 | 18.44816 |
| O5 | 8.248652 | 7.604123 | 18.64331 | C42 | 3.215216 | 6.873718 | 19.06850 |
| O6 | 6.242104 | 7.079616 | 17.64684 | H43 | 4.245439 | 7.222064 | 19.23553 |
| O7 | 7.342301 | 9.003900 | 17.18427 | C44 | 2.130098 | 7.671419 | 19.46940 |
| C8 | 8.536170 | 3.887526 | 20.91247 | H45 | 2.318735 | 8.641847 | 19.95373 |
| C9 | 8.672899 | 4.893734 | 21.89757 | C46 | 0.812628 | 7.230599 | 19.25637 |
| H10 | 7.799985 | 5.488780 | 22.20481 | H47 | −0.037140 | 7.854674 | 19.57383 |
| C11 | 9.919214 | 5.133276 | 22.49647 | C48 | 0.582200 | 5.987622 | 18.63988 |
| H12 | 10.01269 | 5.917962 | 23.26304 | H49 | −0.447310 | 5.635448 | 18.47154 |
| C13 | 11.04222 | 4.375031 | 22.11890 | C50 | 1.663464 | 5.185775 | 18.23842 |
| H14 | 12.01910 | 4.562698 | 22.59100 | H51 | 1.466200 | 4.212925 | 17.76464 |
| C15 | 10.91242 | 3.375125 | 21.14010 | C52 | 3.845555 | 2.894049 | 18.04007 |
| H16 | 11.78540 | 2.773315 | 20.84353 | C53 | 4.030940 | 1.969928 | 16.98862 |
| C17 | 9.666223 | 3.130762 | 20.53671 | H54 | 4.543777 | 2.275056 | 16.06538 |
| H18 | 9.581121 | 2.338491 | 19.77950 | C55 | 3.546049 | 0.655910 | 17.10564 |
| C19 | 6.988777 | 2.019733 | 19.32460 | H56 | 3.692063 | −0.051080 | 16.27449 |
| C20 | 7.652078 | 1.995494 | 18.07497 | C57 | 2.870588 | 0.250099 | 18.26918 |
| H21 | 8.011029 | 2.936203 | 17.62889 | H58 | 2.483136 | −0.776980 | 18.35423 |
| C22 | 7.859858 | 0.779808 | 17.40436 | C59 | 2.688363 | 1.163240 | 19.32286 |
| H23 | 8.384873 | 0.773868 | 16.43678 | H60 | 2.157969 | 0.856291 | 20.23748 |
| C24 | 7.393119 | −0.422520 | 17.96530 | C61 | 3.173653 | 2.475949 | 19.21196 |
| H25 | 7.553365 | −1.376250 | 17.43904 | H62 | 3.007630 | 3.181111 | 20.03872 |
| C26 | 6.712954 | −0.401400 | 19.19488 | C63 | 4.538533 | 4.898478 | 16.07155 |
| H27 | 6.332774 | −1.337080 | 19.63302 | C64 | 3.436353 | 5.341063 | 15.30907 |
| C28 | 6.510802 | 0.812444 | 19.87529 | H65 | 2.477463 | 5.571052 | 15.79525 |
| H29 | 5.973810 | 0.807121 | 20.83398 | C66 | 3.559435 | 5.503177 | 13.91837 |
| C30 | 5.823091 | 3.475794 | 21.65627 | H67 | 2.694787 | 5.855566 | 13.33488 |
| C31 | 6.183022 | 2.577661 | 22.68968 | C68 | 4.778334 | 5.223147 | 13.27647 |
| H32 | 7.083862 | 1.952695 | 22.59659 | H69 | 4.871646 | 5.353820 | 12.18717 |
| C33 | 5.405438 | 2.494885 | 23.85536 | C70 | 5.880932 | 4.785124 | 14.03118 |
| H34 | 5.691178 | 1.789353 | 24.65073 | H71 | 6.841433 | 4.571456 | 13.53743 |
| C35 | 4.276188 | 3.319849 | 24.01354 | C72 | 5.764701 | 4.629485 | 15.42214 |
| H36 | 3.673361 | 3.257701 | 24.93280 | H73 | 6.633792 | 4.303940 | 16.01386 |

**Table S7.** Coordinates for the DFT-optimized geometry of **1** with $CH_3CN$ implicit solvation.

| Atom Number | Coordinates (Å) x | y | z | Atom Number | Coordinates (Å) x | y | z |
|---|---|---|---|---|---|---|---|
| Pt0 | 6.391863 | 5.372667 | 18.79433 | C37 | 3.929144 | 4.234849 | 23.00656 |
| P1 | 6.871037 | 3.642857 | 20.16465 | H38 | 3.05742 | 4.895162 | 23.13174 |
| P2 | 4.431408 | 4.623563 | 17.88883 | C39 | 4.698096 | 4.313618 | 21.83207 |
| O3 | 8.098871 | 6.315410 | 19.37413 | H40 | 4.435220 | 5.042346 | 21.05085 |
| C4 | 7.232499 | 7.926329 | 17.77116 | C41 | 2.992591 | 5.619718 | 18.44861 |
| O5 | 8.248168 | 7.607949 | 18.63698 | C42 | 3.213840 | 6.857151 | 19.09337 |
| O6 | 6.242813 | 7.082404 | 17.64046 | H43 | 4.243719 | 7.196208 | 19.28100 |
| O7 | 7.345391 | 9.004461 | 17.17414 | C44 | 2.127796 | 7.652619 | 19.49646 |
| C8 | 8.534758 | 3.893036 | 20.90795 | H45 | 2.314741 | 8.612907 | 20.00128 |
| C9 | 8.667630 | 4.883145 | 21.90993 | C46 | 0.810984 | 7.221323 | 19.26064 |
| H10 | 7.790154 | 5.462002 | 22.23498 | H47 | −0.039710 | 7.843230 | 19.57985 |
| C11 | 9.915541 | 5.125850 | 22.50463 | C48 | 0.582177 | 5.990099 | 18.62015 |
| H12 | 10.00560 | 5.896683 | 23.2856 | H49 | −0.446840 | 5.645227 | 18.43477 |
| C13 | 11.04409 | 4.386542 | 22.10609 | C50 | 1.664336 | 5.190303 | 18.21715 |
| H14 | 12.02195 | 4.575574 | 22.57563 | H51 | 1.468252 | 4.226234 | 17.72527 |
| C15 | 10.91820 | 3.402881 | 21.11025 | C52 | 3.843286 | 2.889977 | 18.04044 |
| H16 | 11.79530 | 2.815329 | 20.79765 | C53 | 4.029112 | 1.964261 | 16.99041 |
| C17 | 9.670484 | 3.155534 | 20.51103 | H54 | 4.545790 | 2.265819 | 16.06818 |
| H18 | 9.589048 | 2.375071 | 19.7412 | C55 | 3.539350 | 0.652013 | 17.10771 |
| C19 | 6.985451 | 2.019710 | 19.32597 | H56 | 3.685440 | −0.056530 | 16.27789 |
| C20 | 7.644216 | 1.993389 | 18.07397 | C57 | 2.859058 | 0.250012 | 18.26989 |
| H21 | 7.999937 | 2.933065 | 17.62323 | H58 | 2.468167 | −0.775740 | 18.35514 |
| C22 | 7.851910 | 0.776182 | 17.40601 | C59 | 2.676305 | 1.165094 | 19.32190 |
| H23 | 8.372960 | 0.768623 | 16.43632 | H60 | 2.142041 | 0.861032 | 20.23521 |
| C24 | 7.389821 | −0.425470 | 17.97231 | C61 | 3.165948 | 2.476179 | 19.21082 |
| H25 | 7.549894 | −1.380360 | 17.44813 | H62 | 2.998957 | 3.183030 | 20.03596 |
| C26 | 6.714304 | −0.402330 | 19.20454 | C63 | 4.543666 | 4.895424 | 16.07236 |
| H27 | 6.337788 | −1.337480 | 19.64693 | C64 | 3.452365 | 5.364580 | 15.30993 |
| C28 | 6.511902 | 0.813009 | 19.88225 | H65 | 2.497446 | 5.612759 | 15.79491 |
| H29 | 5.978601 | 0.808704 | 20.84298 | C66 | 3.580883 | 5.528859 | 13.91975 |
| C30 | 5.821954 | 3.479176 | 21.65745 | H67 | 2.724359 | 5.900970 | 13.33651 |
| C31 | 6.185464 | 2.583168 | 22.69167 | C68 | 4.794230 | 5.224717 | 13.27826 |
| H32 | 7.086860 | 1.959069 | 22.59848 | H69 | 4.891425 | 5.356074 | 12.18937 |
| C33 | 5.409440 | 2.500505 | 23.85841 | C70 | 5.886225 | 4.760318 | 14.03296 |
| H34 | 5.697575 | 1.796356 | 24.65411 | H71 | 6.842105 | 4.526152 | 13.53947 |
| C35 | 4.278355 | 3.323017 | 24.01667 | C72 | 5.764875 | 4.602449 | 15.42329 |
| H36 | 3.676508 | 3.260438 | 24.93654 | H73 | 6.626153 | 4.254528 | 16.01361 |

**Table S8.** Coordinates for the DFT-optimized geometry of $Pt(PPh_3)(CH_3CN)(CO_3)$ with $CH_3CN$ implicit solvation.

| Atom Number | Coordinates (Å) x | y | z | Atom Number | Coordinates (Å) x | y | z |
|---|---|---|---|---|---|---|---|
| Pt0 | 17.77706 | 6.138994 | 5.291161 | C23 | 21.10941 | 3.708401 | 1.024470 |
| H1 | 19.75510 | 6.365932 | 7.718177 | H24 | 22.85222 | 5.009478 | 0.891570 |
| P2 | 19.98729 | 6.126922 | 4.833729 | C25 | 21.93756 | 4.761908 | 1.452108 |
| O3 | 15.74625 | 6.626915 | 5.520414 | C26 | 20.99919 | 5.428807 | 6.188747 |
| O4 | 17.25843 | 7.970026 | 4.632246 | H27 | 22.25195 | 6.332832 | 2.916442 |
| O5 | 15.09916 | 8.695821 | 4.789931 | C28 | 22.52431 | 4.435675 | 8.343845 |
| H6 | 21.11740 | 5.498688 | 9.625208 | C29 | 22.12320 | 4.610407 | 5.945300 |
| H7 | 23.75243 | 3.474787 | 6.822939 | H30 | 22.41126 | 4.351724 | 4.915955 |
| C8 | 22.87980 | 4.115897 | 7.021934 | C31 | 20.62042 | 7.817729 | 4.548708 |
| H9 | 17.51007 | 2.016465 | 8.160360 | C32 | 15.95354 | 7.826919 | 4.971753 |
| H10 | 18.77600 | 1.468255 | 6.995425 | C33 | 20.63866 | 5.738725 | 7.520985 |
| H11 | 17.02860 | 1.32964 | 6.561225 | C34 | 20.42696 | 5.197803 | 3.320496 |
| N12 | 17.84995 | 4.340421 | 6.041596 | C35 | 20.15481 | 8.526301 | 3.416019 |
| C13 | 17.78189 | 1.949692 | 7.087631 | C36 | 21.52840 | 8.438089 | 5.433554 |
| C14 | 17.82223 | 3.275796 | 6.506153 | C37 | 20.59984 | 9.834672 | 3.172674 |
| C15 | 21.40330 | 5.248896 | 8.591853 | C38 | 21.96665 | 9.750936 | 5.185805 |
| H16 | 23.11920 | 4.046103 | 9.184478 | C39 | 21.50526 | 10.44996 | 4.057423 |
| C17 | 21.60032 | 5.506807 | 2.594276 | H40 | 19.44639 | 8.053222 | 2.719516 |
| H18 | 18.67320 | 3.906627 | 3.433496 | H41 | 20.23594 | 10.37758 | 2.286726 |
| C19 | 19.59624 | 4.14221 | 2.883349 | H42 | 21.90061 | 7.898264 | 6.316166 |
| H20 | 19.28392 | 2.581805 | 1.405683 | H43 | 21.85200 | 11.47706 | 3.864639 |
| C21 | 19.93940 | 3.400298 | 1.740738 | H44 | 22.67775 | 10.22574 | 5.879351 |
| H22 | 21.37455 | 3.129485 | 0.126205 | | | | |

## E.2. Compound 2 and Related Calculations

**Table S9.** Coordinates for the DFT-optimized geometry of **2** with $CH_2Cl_2$ implicit solvation.

| Atom Number | Coordinates (Å) x | y | z | Atom Number | Coordinates (Å) x | y | z |
|---|---|---|---|---|---|---|---|
| Pt0 | 5.152997 | 3.284714 | 4.760859 | H29 | 2.403874 | 3.689096 | 8.318344 |
| O1 | 3.434310 | 2.208279 | 4.844314 | C30 | 5.838000 | 5.930718 | 6.639219 |
| O2 | 3.368775 | 1.250950 | 3.699952 | H31 | 5.407451 | 6.674203 | 5.939040 |
| O3 | 5.383115 | 2.156855 | 3.046992 | H32 | 5.857171 | 6.386046 | 7.648449 |
| O4 | 4.361307 | 0.520346 | 1.859084 | C33 | 7.231077 | 5.483134 | 6.182439 |
| C5 | 4.418304 | 1.301637 | 2.815242 | H34 | 7.696788 | 4.821366 | 6.941009 |
| P6 | 4.728701 | 4.454226 | 6.620827 | H35 | 7.904510 | 6.346366 | 6.015901 |
| P7 | 7.056166 | 4.488784 | 4.631253 | C36 | 8.613338 | 3.553878 | 4.437943 |
| C8 | 5.068157 | 3.557222 | 8.174691 | C37 | 8.641293 | 2.485574 | 3.511411 |
| C9 | 5.410171 | 2.188304 | 8.133600 | H38 | 7.730403 | 2.219366 | 2.952979 |
| H10 | 5.455920 | 1.672259 | 7.161993 | C39 | 9.824687 | 1.756409 | 3.313891 |
| C11 | 5.690465 | 1.494435 | 9.323452 | H40 | 9.836773 | 0.925159 | 2.592127 |
| H12 | 5.956168 | 0.427005 | 9.282434 | C41 | 10.98594 | 2.080720 | 4.038867 |
| C13 | 5.633227 | 2.162879 | 10.55818 | H42 | 11.91159 | 1.504726 | 3.884795 |
| H14 | 5.855238 | 1.619751 | 11.48991 | C43 | 10.96159 | 3.139202 | 4.963118 |
| C15 | 5.290857 | 3.527475 | 10.60526 | H44 | 11.86679 | 3.396709 | 5.534521 |
| H16 | 5.242845 | 4.052739 | 11.57162 | C45 | 9.781441 | 3.876376 | 5.164799 |
| C17 | 5.006929 | 4.224730 | 9.420512 | H46 | 9.785687 | 4.702817 | 5.890150 |
| H18 | 4.730935 | 5.289474 | 9.473427 | C47 | 7.063181 | 5.711463 | 3.264484 |
| C19 | 3.013163 | 5.062673 | 6.732805 | C48 | 8.243092 | 6.417512 | 2.936351 |
| C20 | 2.581735 | 6.074979 | 5.843321 | H49 | 9.181239 | 6.215110 | 3.476131 |
| H21 | 3.280990 | 6.516691 | 5.116961 | C50 | 8.226233 | 7.377052 | 1.911933 |
| C22 | 1.251452 | 6.520434 | 5.874032 | H51 | 9.148913 | 7.922747 | 1.660565 |
| H23 | 0.927137 | 7.313621 | 5.183015 | C52 | 7.036789 | 7.636458 | 1.204927 |
| C24 | 0.335791 | 5.951469 | 6.778512 | H53 | 7.028554 | 8.386542 | 0.398866 |
| H25 | −0.708310 | 6.300216 | 6.798154 | C54 | 5.862529 | 6.933680 | 1.524350 |
| C26 | 0.754940 | 4.933256 | 7.651458 | H55 | 4.931399 | 7.129230 | 0.970501 |
| H27 | 0.041261 | 4.479046 | 8.355936 | C56 | 5.873784 | 5.972912 | 2.550809 |
| C28 | 2.087768 | 4.487448 | 7.631245 | H57 | 4.958801 | 5.412943 | 2.801514 |

**Table S10.** Coordinates for the DFT-optimized geometry of **2** with $CH_3CN$ implicit solvation.

| Atom Number | Coordinates (Å) x | y | z | Atom Number | Coordinates (Å) x | y | z |
|---|---|---|---|---|---|---|---|
| Pt0 | 5.155752 | 3.287008 | 4.764026 | H29 | 2.398588 | 3.666748 | 8.292251 |
| P1 | 3.430219 | 2.212340 | 4.845215 | H30 | 5.841956 | 5.934841 | 6.639854 |
| P2 | 3.360376 | 1.258445 | 3.697158 | C31 | 5.413100 | 6.677969 | 5.938393 |
| O3 | 5.377588 | 2.156047 | 3.043669 | H32 | 5.859948 | 6.389896 | 7.649140 |
| O4 | 4.344591 | 0.528302 | 1.853097 | H33 | 7.234946 | 5.484887 | 6.185955 |
| O5 | 4.409504 | 1.308474 | 2.812312 | C34 | 7.699206 | 4.823791 | 6.945906 |
| C6 | 4.733475 | 4.459428 | 6.623663 | C35 | 7.909570 | 6.346822 | 6.018071 |
| C7 | 7.060854 | 4.490270 | 4.636304 | H36 | 8.614867 | 3.551089 | 4.441662 |
| H8 | 5.071099 | 3.562251 | 8.177047 | C37 | 8.646748 | 2.495225 | 3.500954 |
| C9 | 5.441638 | 2.200724 | 8.137698 | H38 | 7.740336 | 2.241105 | 2.930050 |
| H10 | 5.510320 | 1.687369 | 7.165998 | C39 | 9.828447 | 1.763301 | 3.303628 |
| C11 | 5.722590 | 1.510678 | 9.329658 | H40 | 9.843766 | 0.941911 | 2.570659 |
| H12 | 6.011232 | 0.449123 | 9.290435 | C41 | 10.98485 | 2.073179 | 4.042852 |
| C13 | 5.637084 | 2.175911 | 10.56459 | H42 | 11.90949 | 1.495521 | 3.888903 |
| H14 | 5.859349 | 1.635911 | 11.49809 | C43 | 10.95693 | 3.119822 | 4.980395 |
| C15 | 5.266771 | 3.533327 | 10.60964 | H44 | 11.85844 | 3.366793 | 5.562146 |
| H16 | 5.197521 | 4.056103 | 11.57604 | C45 | 9.778164 | 3.859366 | 5.182179 |
| C17 | 4.982624 | 4.226974 | 9.422821 | C46 | 9.780120 | 4.677260 | 5.916981 |
| C18 | 4.686320 | 5.286347 | 9.473498 | H47 | 7.063505 | 5.707881 | 3.265707 |
| H19 | 3.015347 | 5.061332 | 6.727376 | C48 | 8.252612 | 6.381114 | 2.903349 |
| C20 | 2.587589 | 6.083935 | 5.847729 | H49 | 9.199473 | 6.154628 | 3.417581 |
| H21 | 3.291103 | 6.539659 | 5.134376 | C50 | 8.232308 | 7.340564 | 1.878703 |
| C22 | 1.255165 | 6.523800 | 5.873443 | H51 | 9.162000 | 7.860847 | 1.600706 |
| H23 | 0.933947 | 7.325710 | 5.191085 | C52 | 7.030496 | 7.632916 | 1.206347 |
| C24 | 0.334281 | 5.939796 | 6.763010 | H53 | 7.019614 | 8.383065 | 0.400363 |
| H25 | −0.711000 | 6.285209 | 6.779395 | C54 | 5.846532 | 6.963770 | 1.561017 |
| C26 | 0.750204 | 4.911968 | 7.626454 | H55 | 4.905157 | 7.186503 | 1.035428 |
| H27 | 0.032954 | 4.447261 | 8.320419 | C56 | 5.861096 | 6.002981 | 2.587354 |
| C28 | 2.084844 | 4.471257 | 7.611207 | H57 | 4.937840 | 5.470662 | 2.866484 |

**Table S11.** Coordinates for the DFT-optimized geometry of Pt(dppeO)($CH_3CN$)($CO_3$) with $CH_3CN$ implicit solvation.

| Atom Number | Coordinates (Å) x | y | z | Atom Number | Coordinates (Å) x | y | z |
|---|---|---|---|---|---|---|---|
| Pt0 | 17.70189 | 5.946763 | 5.286048 | C32 | 15.82038 | 7.602726 | 5.246513 |
| P1 | 20.53183 | 9.952895 | 2.635079 | O33 | 22.03845 | 9.944273 | 2.527904 |
| P2 | 19.90785 | 6.093404 | 4.858899 | C34 | 20.50061 | 5.095244 | 3.450656 |
| O3 | 15.65824 | 6.327551 | 5.604783 | C35 | 19.57961 | 4.394039 | 2.642941 |
| O4 | 17.11743 | 7.838981 | 4.920911 | H36 | 18.50709 | 4.424502 | 2.889616 |
| O5 | 14.93969 | 8.462799 | 5.207923 | C37 | 20.02948 | 3.666417 | 1.527285 |
| C6 | 19.93029 | 11.19936 | 3.822403 | H38 | 19.30456 | 3.121578 | 0.903254 |
| C7 | 18.72413 | 11.06087 | 4.546107 | C39 | 21.39842 | 3.637299 | 1.211340 |
| H8 | 18.10050 | 10.16030 | 4.435739 | H40 | 21.75043 | 3.067029 | 0.337682 |
| C9 | 18.31417 | 12.07551 | 5.429189 | C41 | 22.32080 | 4.338496 | 2.010910 |
| H10 | 17.37534 | 11.95946 | 5.992480 | H42 | 23.39360 | 4.318900 | 1.764445 |
| C11 | 19.10033 | 13.22925 | 5.596436 | C43 | 21.87750 | 5.066191 | 3.126245 |
| H12 | 18.77579 | 14.02111 | 6.289439 | H44 | 22.60928 | 5.608527 | 3.744750 |
| C13 | 20.30486 | 13.36857 | 4.882569 | C45 | 20.92603 | 5.617724 | 6.300470 |
| H14 | 20.92579 | 14.26813 | 5.015652 | C46 | 21.49445 | 4.327521 | 6.395005 |
| C15 | 20.72048 | 12.35796 | 4.000568 | H47 | 21.38078 | 3.608753 | 5.570026 |
| H16 | 21.66717 | 12.46028 | 3.448057 | C48 | 22.21583 | 3.955971 | 7.542542 |
| C17 | 19.73284 | 10.32256 | 1.035773 | H49 | 22.65799 | 2.949514 | 7.603486 |
| C18 | 20.54720 | 10.37897 | −0.115590 | C50 | 22.37543 | 4.864039 | 8.603682 |
| H19 | 21.63148 | 10.21707 | −0.018410 | H51 | 22.94418 | 4.571649 | 9.499904 |
| C20 | 19.97462 | 10.64214 | −1.372310 | C52 | 21.80473 | 6.146829 | 8.518202 |
| H21 | 20.61523 | 10.68618 | −2.266900 | H53 | 21.92343 | 6.862244 | 9.346524 |
| C22 | 18.58845 | 10.84892 | −1.485420 | C54 | 21.07824 | 6.523076 | 7.376807 |
| H23 | 18.14040 | 11.05543 | −2.469940 | H55 | 20.62861 | 7.526468 | 7.334442 |
| C24 | 17.77282 | 10.79529 | −0.339450 | H56 | 20.09886 | 7.654734 | 2.291845 |
| H25 | 16.68760 | 10.96049 | −0.425120 | H57 | 18.73347 | 8.396717 | 3.169701 |
| C26 | 18.34094 | 10.53522 | 0.918400 | N58 | 17.86376 | 4.059421 | 5.750667 |
| H27 | 17.69404 | 10.50354 | 1.809053 | C59 | 17.89658 | 2.936466 | 6.046899 |
| C28 | 19.83947 | 8.330795 | 3.133680 | C60 | 17.93182 | 1.536538 | 6.417256 |
| H29 | 20.09984 | 8.456180 | 5.307210 | H61 | 17.69191 | 0.906512 | 5.537183 |
| H30 | 21.53145 | 7.827552 | 4.437972 | H62 | 18.94210 | 1.271594 | 6.788289 |
| C31 | 20.42204 | 7.820595 | 4.458313 | H63 | 17.18921 | 1.340419 | 7.216633 |

**Table S12.** Coordinates for the DFT-optimized geometry of Pt(dmpe)($CO_4$) with $CH_3CN$ implicit solvation.

| Atom Number | Coordinates (Å) x | y | z | Atom Number | Coordinates (Å) x | y | z |
|---|---|---|---|---|---|---|---|
| Pt0 | 5.164665 | 3.288584 | 4.773953 | C15 | 7.256555 | 5.484897 | 6.181016 |
| O1 | 3.463051 | 2.163247 | 4.846761 | C16 | 8.583332 | 3.572233 | 4.391867 |
| O2 | 3.435951 | 1.202469 | 3.700138 | C17 | 7.028292 | 5.727177 | 3.265088 |
| O3 | 5.462922 | 2.112794 | 3.089902 | H18 | 2.320624 | 4.299487 | 6.730529 |
| O4 | 4.481368 | 0.451539 | 1.900239 | H19 | 2.928101 | 5.722678 | 7.663666 |
| C5 | 4.512568 | 1.25155 | 2.847086 | H20 | 2.840113 | 5.788816 | 5.855519 |
| P6 | 4.729481 | 4.474272 | 6.617701 | H21 | 4.300439 | 2.653221 | 8.156645 |
| P7 | 7.04392 | 4.514105 | 4.626849 | H22 | 6.019255 | 3.185124 | 8.224351 |
| C8 | 4.971322 | 3.533871 | 8.159344 | H23 | 4.736742 | 4.175733 | 9.031793 |
| H9 | 5.918801 | 6.302993 | 7.736237 | H24 | 8.705820 | 2.846960 | 5.218192 |
| H10 | 5.438895 | 6.705494 | 6.057151 | H25 | 8.528544 | 3.022979 | 3.432170 |
| H11 | 7.923505 | 6.350965 | 5.996972 | H26 | 9.449397 | 4.263378 | 4.368977 |
| C12 | 5.877268 | 5.913906 | 6.698986 | H27 | 6.183932 | 6.429808 | 3.398468 |
| H13 | 7.763642 | 4.816164 | 6.906573 | H28 | 7.982613 | 6.290530 | 3.248427 |
| C14 | 3.041873 | 5.139605 | 6.728152 | H29 | 6.899231 | 5.191784 | 2.304661 |

**Table S13.** $S_n$ from TD-DFT of Pt(dmpe)($CO_4$).

| Excited State (n) | Wavelength (nm) | Oscillator Strength | Excited State (n) | Wavelength (nm) | Oscillator Strength |
|---|---|---|---|---|---|
| 1 | 470.4 | 0.000115 | 16 | 261.0 | 0.000231 |
| 2 | 385.9 | 0.008715 | 17 | 260.4 | 0.008453 |
| 3 | 364.7 | 0.036485 | 18 | 259.6 | 0.012400 |
| 4 | 342.9 | 0.001445 | 19 | 259.4 | 0.012549 |
| 5 | 332.3 | 7.98E−05 | 20 | 257.8 | 0.001806 |
| 6 | 329.0 | 0.000276 | 21 | 254.4 | 0.008096 |
| 7 | 303.3 | 0.000663 | 22 | 252.1 | 0.004215 |
| 8 | 298.3 | 0.004033 | 23 | 248.6 | 0.002052 |
| 9 | 295.4 | 0.002101 | 24 | 246.1 | 6.73E−05 |
| 10 | 293.0 | 0.000578 | 25 | 239.8 | 0.021800 |
| 11 | 283.7 | 0.000195 | 26 | 239.3 | 0.000867 |
| 12 | 279.0 | 0.021703 | 27 | 236.2 | 0.048093 |
| 13 | 275.7 | 7.67E−05 | 28 | 234.3 | 0.022029 |
| 14 | 267.4 | 0.004992 | 29 | 232.1 | 0.015178 |
| 15 | 263.2 | 0.008148 | 30 | 231.5 | 0.000864 |

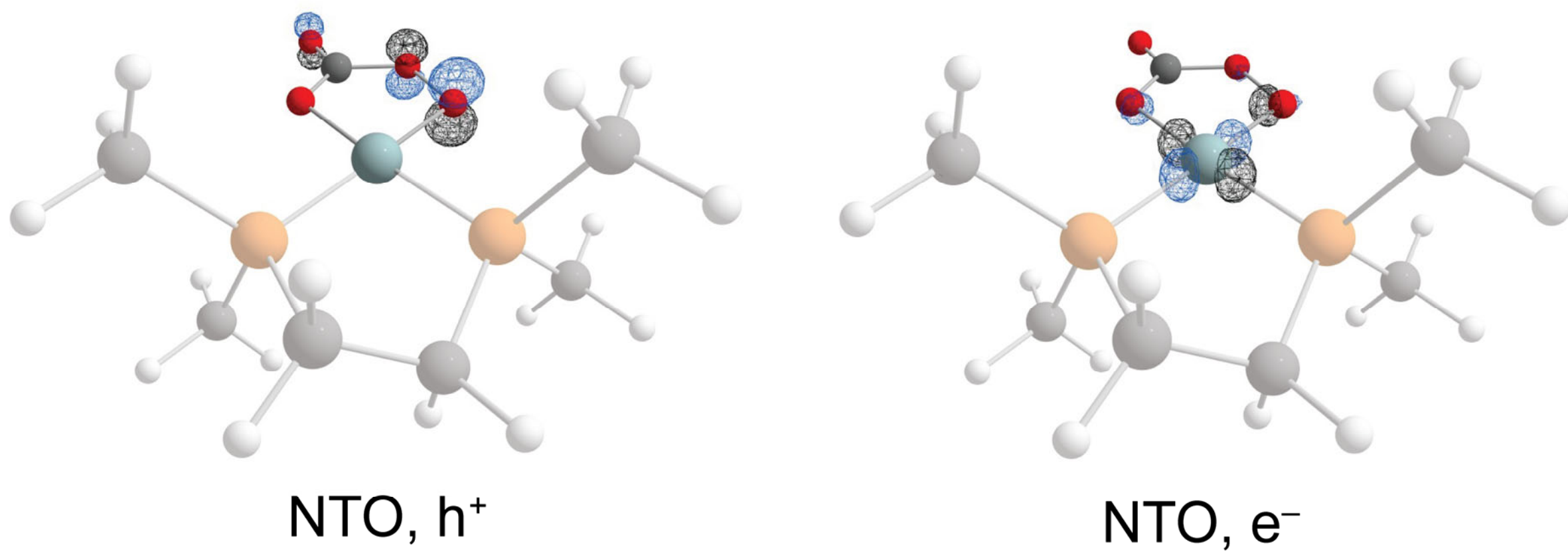


**Figure S27.** (A) Natural transition orbitals (NTOs) of the hole (left) and electron (right) of the lowest energy singlet excited state, $S_1$, from TD-DFT of Pt(dmpe)($CO_4$), showing ππ* antibonding character for the unoccupied ($h^+$) NTO of the peroxy subunit and antibonding character for the occupied ($e^-$) NTO of the $d_{x^2-y^2}$ metal-ligand subunit. NTOs are plotted with an isovalue of 0.14.

## F. References


[31] K. O. Kirlikovali, J. C. Axtell, A. Gonzalez, A. C. Phung, S. I. Khan, A. M. Spokoyny, "Luminescent Metal Complexes Featuring Photophysically Innocent Boron Cluster Ligands." *Chem. Sci.* **2016**, *7*, 5132–5138.

[32] M. A. Andrews, G. L. Gould, W. T. Klooster, K. S. Koenig, E. J. Voss, "Syntheses, Spectra, and Structures of (Diphosphine)platinum(II) Carbonate Complexes." *Inorg. Chem.* **1996**, *35*, 5478–5483.

[33] G. M. Sheldrick, SADABS, Bruker Analytical X-ray Systems (2014).

[34] G. M. Sheldrick, SHELXT - Integrated Space-Group and Crystal-Structure Determination. *Acta Crystallogr. A* **2015**, *71*, 3–8.

[35] G. M. Sheldrick, SHELXL (University of Göttingen, 2014).

[36] O. V. Dolomanov, L. J. Bourhis, R. J. Gildea, J. A. K. Howard, H. Puschmann, "OLEX2: A Complete Structure Solution, Refinement and Analysis Program." *J. Appl. Cryst.* **2009**, *42*, 339–341.

[37] F. Neese, "Software Update: The ORCA Program System, ver. 5.0." *WIRES Comput. Molec. Sci.* **2022**, *12*, e1606.

[38] F. Neese, "The SHARK Integral Generation and Digestion System." *J. Comput. Chem.* **2023**, *44*, 381–396.

[39] F. Neese, "An Improvement of the Resolution of the Identity Approximation for the Formation of the Coulomb Matrix." *J. Comput. Chem.* **2003**, *24*, 1740–1747.

[40] J. P. Perdew, "Density-Functional Approximation for the Correlation Energy of the Inhomogeneous Electron Gas." *Phys. Rev. B* **1986**, *33*, 8822–8824.

[41] A. D. Becke, "Completely Numerical Calculations on Diatomic Molecules in the Local-Density Approximation." *Phys. Rev. A* **1986**, *33*, 2786–2788.

[42] A. D. Becke, "Density-Functional Exchange-Energy Approximation with Correct Asymptotic Behavior." *Phys. Rev. A* **1988**, *38*, 3098–3100.

[43] F. Weigend, R. Ahlrichs, "Balanced Basis Sets of Split Valence, Triple Zeta Valence and Quadruple Zeta Valence Quality for H to Rn: Design and Assessment of Accuracy." *Phys. Chem. Chem. Phys.* **2005**, *7*, 3297–3305.

[44] E. van Lenthe, J. G. Snijders, E. J. Baerends, "The Zero-Order Regular Approximation for Relativistic Effects: The Effect of Spin–Orbit Coupling in Closed Shell Molecules." *J. Chem. Phys.* **1996**, *105*, 6505–6516.

[45] M. Garcia-Ratés, F. Neese, "Effect of the Solute Cavity on the Solvation Energy and its Derivatives within the Framework of the Gaussian Charge Scheme." *J. Comput. Chem.* **2020**, *41*, 922–939.

[46] Chemcraft - Graphical Software for Visualization of Quantum Chemistry Computations, ver. 1.8, build 682.
